# CD3ɛ Nanobody‐Engineered Extracellular Vesicles Driving In Vivo Generation of TCE‐secreting CAR‐Ts for Solid Tumor Therapy With Memory Response and Minimal Immunogenicity

**DOI:** 10.1002/advs.202519440

**Published:** 2026-02-03

**Authors:** Shi‐Wei Huang, Yu‐Chuan Lin, Chih‐Ming Pan, Yeh Chen, Ming‐You Shie, Cheng‐Yu Chen, Kai‐Wen Kan, Yi‐Wen Chen, Ming‐Chao Liu, Chung‐Chun Wu, Yu‐Ting Chiang, Huai‐Ping Ho, Chen‐Yu Lin, Pei‐Ying Lin, Yu‐Han Huang, Steffany Rusli, Wan‐Yu Mao, Pei‐Wen Huang, Sin‐Ting Wang, Wan‐Chen Tsai, Ya‐Hsu Chiu, Ting‐Hsun Lin, Wan‐Ling Chiang, Che‐Kai Chang, Zi‐Lun Lai, Mei‐Chih Chen, Shao‐Chih Chiu, Der‐Yang Cho

**Affiliations:** ^1^ Translational Cell Therapy Center Department of Medical Research China Medical University Hospital Taichung Taiwan; ^2^ Institute of Biomedical Science National Chung‐Hsing University Taichung Taiwan; ^3^ Institute of New Drug Development China Medical University Taichung Taiwan; ^4^ Department of Research and Development Shine‐On BioMedical Co. Ltd. Taichung Taiwan; ^5^ Department of Food Science and Biotechnology National Chung‐Hsing University Taichung Taiwan; ^6^ Research & Development Center for x‐Dimensional Extracellular Vesicles Department of Medical Research China Medical University Hospital Taichung Taiwan; ^7^ Department of Manufacturing Production & Manufacturing Division Ever Supreme Bio Technology Taichung Taiwan; ^8^ Shine Out Bio Medical International Co. Ltd. Taichung Taiwan; ^9^ Department of Laboratory Medicine China Medical University Hospital Taichung Taiwan; ^10^ Graduate Institute of Biomedical Sciences China Medical University Taichung Taiwan; ^11^ Department of Neurosurgery China Medical University Hospital Taichung Taiwan

**Keywords:** CD3, chimeric antigen receptor, extracellular vesicles, in vivo, T cell memory

## Abstract

In vivo generation of chimeric antigen receptor‐T (CAR‐T) cells offers an innovative approach to CAR‐T therapy; however, current in vivo CAR‐T technologies rely on synthetic carriers or viral particles, which raise immunogenicity and safety concerns in clinical applications. Extracellular vesicles (EVs) are cell‐derived natural nano‐platforms with improved biocompatibility and the potential to deliver the transgene for in vivo CAR‐T generation. In this study, we pioneered a CD3ε nanobody (Nb)‐CD63 chimeric construct and stably expressed it on HEK‐293T cell‐derived EVs to produce CD3ε‐targeting EVs, which were further loaded with Nb‐CAR.TCE (Nb‐CAR with secretable bispecific T‐cell engager) transgene through electroporation. The CD3ε‐Nb EVs selectively delivered Nb‐CAR.TCE transgene into CD3^+^ cells in vivo and exerted robust antitumor activity against various solid tumors. The CD3‐targeting property of CD3ε‐Nb EVs combined with the characteristics of Nb‐CAR.TCE construct may enhance memory CAR‐T proportion, prolong anti‐tumor immunity, and strengthen resilience against tumor antigen rechallenge. Notably, the CD3ε‐Nb EVs exhibited minimal immunogenicity risks compared to lipid‐based and lentiviral carriers, despite their comparable anti‐tumor activity. Taken together, the CD3‐targeting EVs could drive the in vivo generation of bispecific CAR‐T cells to effectively eliminate cancers and improve memory response with minimal immunogenicity.

## Graphical abstract

HEK‐293T‐derived CD3ε Nb‐engineered EVs to generate dual‐targeting CAR‐T cells directly in vivo. These EVs selectively deliver CAR.BiTE transgenes into T cells and reprogramed to HLA‐G/PD‐L1‐targeting effector cells with enhanced memory and persistence. This strategy effectively eliminates solid tumors in mice with minimal toxicity or immunogenicity, offering a safer, scalable alternative to traditional ex vivo CAR‐T manufacturing.

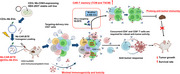

**Abbreviations**
BiTE: bispecific T cell engager
CAR: chimeric antigen receptor
CDX: cell line‐derived xenograft
CRC: colorectal cancer
CRS: cytokine release syndrome
DAP‐12: DNAX‐activating protein 12
ELISA: enzyme‐linked immunosorbent assay
EVs: extracellular vesicles
FACS: fluorescence‐activated cell sorting
GBM: glioblastoma
γδT: gamma‐delta T
GvHD: graft versus host disease
HLA: human leukocyte antigen
HPF: high‐power fields
ICD: intracellular domain
ICP: immune checkpoint protein
IFNAR1: interferon‐alpha/beta receptor 1
IVIS: in vivo imaging system
LNPs: lipid nanoparticles
LVs: lentivirus
MAIT: mucosal‐associated invariant T
Nb: nanobody
NK: natural killer
NLuc: nano‐luciferase
NSCLC: non‐small‐cell lung cancer
NSG: NOD/SCID gamma
P: porcine teschovirus‐1 2A
PBMC: peripheral blood mononuclear cells
PD‐1: programmed death‐1
PD‐L1: programmed death‐ligand 1
PI: propidium iodide
scFv: single‐chain variable fragment
SPR: surface Plasmon Resonance
TAA: tumor‐associated antigens
TCR: T cell receptor
TCM: T central memory
TEM: T effector memory
TME: tumor microenvironment
Treg: regulatory T
TSCM: T stem cell memory
TTE: terminal effector memory
TU: transducing unit
TUNEL: terminal deoxynucleotidyl transferase dUTP nick end labeling

## 1 Introduction

Chimeric antigen receptor (CAR) T‐cell therapy has exhibited remarkable clinical responses in B‐cell malignancies, opening a new landscape in engineered T cells as a therapeutic modality [1]. In addition to hematological cancers, a growing number of clinical trials are leveraging CAR‐T therapies to treat solid tumors and autoimmune diseases [2, 3]. So far, all available CAR‐T cell products are personalized medicines that require collecting T cells from the patient's blood, and treatment can begin only after genetic manipulation and ex vivo T cell expansion [4]. Although accelerated manufacturing processes have advanced [5], their feasibility is limited, particularly for patients with rapidly progressive diseases. Consequently, the rise of off‐the‐shelf allogeneic CAR‐T therapies presents key advantages, such as immediate access to standardized cell products [6, 7]. We have successfully established a nanobody‐based allogeneic CAR.BiTE‐γδT therapy, currently undergoing a phase I/IIa clinical trial (NCT06150885) [8], but concerns about alloimmune responses and the risk of life‐threatening graft‐versus‐host disease (GvHD) may require careful monitoring and management. Additionally, donor heterogeneity may pose challenges in clinical practice. In vivo generation of CAR‐T cells represents a promising breakthrough for overcoming the difficulties associated with ex vivo cell manufacturing and has been successfully demonstrated in preclinical models [9]. Compared to off‐the‐shelf allogeneic CAR‐T therapies, in vivo‐generated autologous CAR‐T cells are compatible with the patient's immune system, thus mitigating the risk of GvHD and unfavorable allograft rejection [9, 10].

To date, the generation of in vivo CAR‐T cells has relied on genetic engineering using viral vectors or synthetic biomaterials such as lipid‐based carriers and implantable bioinstructive scaffolds [9, 10, 11, 12]. The potential of in vivo CAR‐T therapies has been demonstrated in preclinical settings for hematological cancers and cardiac injury. However, utilizing viral particles or artificial biomaterials raises safety concerns, including genotoxicity, immunogenicity, and invasiveness [9, 10, 11, 12, 27, 28, 29]. In addition, scaffold implantation for in vivo CAR‐T generation, while innovative, still encounters challenges similar to those of conventional ex vivo methods regarding the availability and management of autologous T cells [13, 14]. Accordingly, developing an innovative transgene delivery system that exhibits minimal invasiveness, high biocompatibility, and favorable tolerance in human subjects is imperative. Such a system should mitigate concerns of genotoxicity and immunogenicity, facilitating the in vivo generation of CAR‐T cells against solid tumors and potentially enabling more rapid treatment initiation.

Extracellular vesicles (EVs) are natural nanoparticles derived from cells and enclosed by a lipid bilayer. They transport cellular materials such as proteins, RNA, and DNA from donor cells to recipient cells [15]. EVs have been applied in the diagnosis, therapeutic, and targeted delivery [16, 17, 18]. The flexibility of engineering targeting moieties for redirection toward specific cells, and their capacity to be loaded with therapeutic components, such as small‐molecule drugs or nucleic acids, makes them an attractive drug‐delivery platform [19, 20]. In addition, EV‐based therapies have been demonstrated to have tolerable toxicity and therapeutic benefits in clinical studies [19, 21]. Notably, the characteristics of the donor cells are reflected in the cargo and functions of EVs [16, 17], which may affect the therapeutic efficiency and targeting specificity of engineered EVs. Therefore, characterization and standardization of the donor cells and EV cargo are critical for developing EV‐based therapeutics.

Cell line‐derived EVs, with standardized and straightforward sources, have demonstrated beneficial capacities in clinical practice [22]. Moreover, EVs have been used as in vivo delivery platforms for nucleic acids to enable genetic manipulation [23]. However, the size of nucleic acids limits the loading efficiency and capacity [24]. Conventional CAR‐T is encoded by a large vector that reflects the complexity of the CAR‐T functional domains. Therefore, successful EV‐based platforms have not yet been developed to deliver DNA for CAR‐T engineering.

In the present study, we employed the HEK‐293T cell line to establish a stable clone expressing CD3ε Nb fusion‐CD63 chimeric proteins for continuous production of CD3‐targeting EVs. Through electroporation, the CD3‐targeting EVs were further loaded with linearized Nb‐CAR.TCE DNA, encoding a nanobody (Nb)‐based secretable bispecific T cell engager (BiTE) following a bicistronic CAR, which consists of a bi‐epitopic Nb for targeting a neoexpressed immune checkpoint (ICP) HLA‐G on tumor cells. A Tyk‐binding motif derived from IFNAR1 was inserted into the CAR intracellular domain (ICD) to improve persistence, and a secretable bivalent Nb‐BiTE was used to block PD‐1/PD‐L1 axis and recruit bystander effector cells against PD‐L1‐expressing tumor cells [8] (illustrated in Figure S1A). The antitumor activity of the EV‐driven in vivo‐generated dual ICP‐targeting CAR‐T and the subsequent modulation of memory CAR‐T were evaluated in both in vitro and in vivo solid tumor models. This groundbreaking EV‐driven in vivo CAR‐T therapy initiative could usher in a new era of cancer treatment.

## 2 Materials and Methods

### 2.1 Reagents and Antibodies

Puromycin, G418, PNGase F, and luciferin were obtained from Sigma‐Aldrich. Nano‐Glo Fluorofurimazine In Vivo Substrate (FFz) was purchased from Promega. Antibody against β‐actin (C4) was purchased from Santa Cruz Biotechnology. HLA‐G (MEM‐G/2), p‐Syl/ZAP70 (Zap70 Y319‐A3) were purchased from Thermo Fisher Scientific. CD3 (HIT3a), CD4 (SK3), CD8α (SK1), CD56 (HCD56), CD66b (6/40c), CD14 (61D3), TCRγδ (B1), and TCRαβ (IP26) specific antibodies were obtained from BioLegend. Antibodies against CD27 (M‐T271), CD45RA (HI100), HER2 (Trastuzumab297.rMAb), CD86 (2331 (FUN‐1)), and HLA‐DR (G46‐6) were obtained from BD Pharmingen. Human CD63 (ARC51703), mouse CD63 (EPR21151), human CD81 (A5270), mouse CD81 (SN206‐01), human/mouse CD9 (ARC0330), human/mouse TSG101 (A1692), VSV‐G (#81454), Foxp3 (NB100‐39002), mouse CD31 (EPR17260‐263), α‐SMA (#19245), p‐STAT2(Y690) (D3P2P), and p‐Tyk2 (Tyr1054/1055) (D7T8A) antibodies were purchased from Abclonal, Abcam, Novus Biologicals, or Cell Signaling Technology. The anti‐VHH antibodies were obtained from Jackson ImmunoResearch (128‐005‐230, 128‐005‐232) and GenScript (A02014), respectively. Recombinant human HLA‐G, interleukin 2 (IL‐2), IL‐4, and TGF‐β were obtained from Creative Biomart and R&D Systems, respectively.

### 2.2 Cell Lines and PBMC Donors

The human colon cancer cell lines COLO 205 and Luc‐expressing COLO 205, human lung tumor cell lines A549, A549 PD‐L1 KO, A549 HLA‐G KO, A549 HLA‐G/PD‐L1 DKO, and H1975, human pancreatic carcinoma cell lines AsPC‐1, Panc‐1 and Luc‐expressing Panc‐1, and glioblastoma cell line GBM8901 were cultured in RPMI‐1640 medium in the presence with or without 4–8 µg/mL puromycin. The human glioblastoma cell line DBTRG‐05MG, mouse glioblastoma cell line GL261, and HLA‐G‐overexpressing GL261 were maintained in DMEM. The human colon cancer cell line HCT‐116 was cultured in Mycoy's 5A medium. All the media were supplemented with 10% FBS (Hyclone). The human embryonic kidney cell line HEK‐293T was cultured in FreeStyle 293 Expression Medium supplemented with 10% Exosome‐Depleted FBS (Thermo Fisher Scientific).

PBMCs derived from each healthy donor (age from 20 to 50) were collected through the Translation Cell Therapy Center (CMUH110‐REC2‐001) and isolated from whole blood using density gradient centrifugation with Ficoll‐Paque Plus (GE Healthcare Life Sciences) with approval from the Research Ethics Committee of the China Medical University Hospital (CMUH), Taichung, Taiwan (CMUH112‐REC3‐186).

### 2.3 Construction of CD3ε Nb‐CD63, CD3ε Nb‐Mouse CD63, CD63‐NLuc, CD3ε Nb‐CD63‐NLuc‐Expressing Vectors, Nb‐CAR.TCE, Nb‐CAR(WT).TCE, Nb‐CAR, Nb‐CAR(WT) and Nb‐BiTE Transgene

HLA‐G, PD‐L1, and CD3ε‐specific Nbs were generated using the HuSdL Human Single Domain Antibody Library (Creative Biolabs) as described in a previous study [8]. Regarding the CD3ε Nb‐CD63 construction, full‐length CD3ε Nb was inserted into CD63 at a.a. 135 (P08962, UniProt), and the chimeric protein was linked with a six‐repeated His‐tag and an AausFP1. For the generation of CD3ε Nb‐mouse CD63 construct, both ends of full‐length CD3ε Nb were connected with a GGGGS linker, which was inserted into the loop between the third and fourth transmembrane domains of mouse CD63 at a.a. 180 (P41731, UniProt). For the CD63‐NLuc construct, Oplophorus‐luciferin 2‐monooxygenase catalytic subunit (Q9GV45, UniProt) was fused to the C‐terminal of full‐length CD63 with a GGGGS linker. While the CD3ε Nb‐CD63‐NLuc construct consisted of full‐length CD3ε Nb, which was inserted into CD63 at a.a. 135, the chimeric construct was fused with a GGGGS linker and followed by full‐length Oplophorus‐luciferin 2‐monooxygenase catalytic subunit. All of these chimeras were cloned into a pEXO plasmid.

Construction of the Nb‐CAR.TCE construct was described previously [8]. Briefly, the bi‐epitopic Nb‐CAR consisted of tandem HLA‐G Nbs followed by the CD8 hinge/transmembrane domain (amino acids 144–203, P01732, UniProt), modified 4‐1BB cytosolic domain (amino acids 214–255 (Q07011, UniProt) with an inserted Tyk‐binding motif from IFNAR1 (amino acids 458–520, P17181, UniProt) at amino acid 230, was designed to increase T‐cell persistence), CD3ξ cytosolic domain (amino acids 52–164, P20963, UniProt) inserted with a ITAM from DAP‐12 (amino acids 62–64 + 80–108 (O43914, UniProt), aimed to additional enhance cytotoxic killing activity) at amino acid 159. This was followed by fusion with a full‐length self‐cleavage peptide P2A, followed by a Nb‐BiTE which consisted of two PD‐L1 Nbs ligated with a GGGGS linker and subsequently linked with CD3ε Nb and a WPRE element. By comparing with Nb‐CAR.TCE construct, the Nb‐CAR(WT).TCE was constructed without the insertion of the IFNAR1‐derived Tyk binding motif; Nb‐CAR transgene was absent of the P2A and Nb‐BiTE elements; Nb‐CAR(WT) was generated without IFNAR1‐derived Tyk binding motif, P2A, and Nb‐BiTE moieties; and the Nb‐BiTE construct only contained the Nb‐BiTE moiety consisting of the PD‐L1 and CD3ε Nbs without Nb‐CAR. These constructs were cloned into the pcDNA3.1 (Addgene) backbone with an EF‐1α promoter at the EcoRI/XbaI cloning site. Subsequently, these plasmids were subjected to the synthesis of linearized transgenes by polymerase chain reaction (PCR) through the following conditions: 35 cycles at 98°C for 20 s, 72°C for 30 s, and 72°C for 3.5 min) using HiFi KAPA DNA polymerase (Roche). The primer pairs were as follows: forward, 5′‐GCTCCGGTGCCCGTCAGTGG‐3′, and reverse, 5′‐GGTACCAGGCGGGGAGGCG‐3′. All PCR products were checked by 1% agarose gel electrophoresis, visualized with ethidium bromide, and purified through gel slice using PureLink Quick Gel Extraction and PCR Purification Combo Kit (Thermo Fisher Scientific).

### 2.4 Generation of Stable Clones Overexpress CD3ε Nb‐CD63, CD3ε Nb‐Mouse CD63, CD63‐NLuc, CD3ε Nb‐CD63‐NLuc, HLA‐G Knockdown, HLA‐G KO, PD‐L1 KO, and HLA‐G/PD‐L1 DKO

To generate CD3ε Nb‐CD63, CD3ε Nb‐mouse CD63, CD63‐NLuc, and CD3ε Nb‐CD63‐NLuc‐overexpressing cells, pExo plasmids encoding CD3ε Nb‐CD63, CD3ε Nb‐mouse CD63, CD63‐NLuc or CD3ε Nb‐CD63‐NLuc chimeric protein were transfected into HEK‐293T or NIH/3T3 cells using Lipofectamine 3000 (L3000015, Invitrogen). To obtain stable HLA‐G knockdown cells, the HLA‐G (sc‐42920‐V) shRNA lentiviral particles (Santa Cruz) were transfected into COLO 205 cells using polybrene (8 µg/mL). The CRISPR and TALEN gene editing toolkit (Thermo Fisher Scientific) was used to establish HLA‐G KO, PD‐L1 KO, and silencing HLA‐G/PD‐L1 DKO cells. Subsequently, stable single‐cell clones were selected using puromycin (1–4 µg/mL for HEK‐293T and NIH/3T3 cells, 2–8 µg/mL for COLO 205) for ≥20 days, followed by the selection of single clone‐derived sub‐cell lines based on the total and membrane‐bound expression levels of VHH, CD63, and HLA‐G determined through immunoblotting and flow cytometry, respectively.

### 2.5 Fabrication and Characterization of Unmodified EVs and CD3ε Nb‐Expressing EVs Derived From HEK‐293 cells and NIH/3T3 Cells

The parental and chimeric CD3ε Nb‐CD63 stable expressing HEK‐293T cells, and the parental and chimeric CD3ε Nb‐mouse CD63 stable expressing NIH/3T3 cells (5 × 10^8^) were loaded in Corning CELLSTACK culture chamber with 5000 mL of FreeStyle 293 Expression Medium (Thermo Fisher Scientific) and DMEM supplemented with 10% Exosome‐Depleted FBS, respectively. After culturing for 3 days, the supernatants were centrifuged at 2000 g for 15 min to remove cellular debris and then filtered with a 0.2 µm filter. The collections were concentrated by tangential flow filtration (MAP.03‐plus TFF System, Lefo Science). Supernatants from CD3ε Nb‐CD63 chimeric protein‐expressing HEK‐293T stable cells or from CD3ε Nb‐mouse CD63‐stably expressing NIH/3T3 cells were purified by an Anti‐Camelid VHH‐Affinity Resin (GenScript) slowly. After washing with Tris‐buffered saline (50 mm Tris‐HCl, 150 mm NaCl, pH 7.4), the bound CD3ε Nb‐expressing EVs were eluted with an acid elution buffer (0.1 m glycine, pH 2.5). The eluted CD3ε Nb‐EV or CD3ε Nb‐mEV supernatants were filtrated through a 30 kDa cut‐off membrane and resuspended in PBS. For unmodified, NLuc and CD3ε‐Nb NLuc EVs, the supernatants from parental or transfected HEK‐293T and NIH/3T3 cells were filtered through a 30 kDa cut‐off membrane and resuspended in PBS.

The EV samples were then characterized by exosomal biomarker analysis through immunoblotting or dot plots. In brief, 20 µg EV samples were treated with PNGase F (5 U/mL) for 30 min and then subjected to immunoblotting using specific antibodies to detect the expression levels of VHH, CD63, CD81, CD9, TSG 101, and β‐actin. The purity of CD3ε Nb‐expressing EVs was further assessed by nano‐flow cytometry with a VHH antibody, while the unmodified EVs served as a background control.

Transmission electron microscopy (TEM) imaging was carried out to identify EVs. Briefly, EVs (with or without Nb‐CAR.TCE transgene payload) were fixed with 1% glutaraldehyde at 4°C overnight. After washing, the EVs were loaded onto formvar carbon‐coated grids and then negatively stained with 2% aqueous phosphotungstic acid solution at 25°C for 1 min. The ultrastructure of these EVs was observed by TEM (JEOL JEM‐1400, Tokyo, Japan) at 100 000×.

### 2.6 NTA Analysis

The harvested and engineered EVs were subjected to Nanoparticle Tracking Analysis (NTA) by ZetaView (Particle Metrix GmbH) to determine their concentration, size distribution, and zeta potential. In brief, the ZetaView NTA system was equipped with a 488 nm laser. The camera level was automatically adjusted, and the analysis detection sensitivity was 85. Settings included position at 11, frame rate at 30, shutter at 100, conductivity at 15 000 muS/cm, and temperature at 25°C for analyzing concentration and size distribution. All the settings for zeta potential were the same as particle concentration and size analysis, while the shutter value was set at 70. EV samples were diluted 8000‐fold in filtered PBS to obtain optimal concentrations (25–80 particles per frame). Two cycles of recordings were carried out for each EV preparation. ZetaView software (Ver 8.05.12 SP1) was used to analyze the recorded data.

### 2.7 CD3ε Nb and Nb‐BiTE Recombinant Protein Production

Codon‐optimized pcDNA3.1‐encoded CD3ε Nb and Nb‐BiTE for recombinant protein was manufactured by Leadgene Biomedical Inc. using a CHO‐K1 expressing system. The quality of the purified CD3ε Nb and Nb‐BiTE was determined by SDS‐PAGE electrophoresis and SPR assay.

### 2.8 Detection of the CD3ε Nb on Engineered EVs by Mass Spectrometry

In brief, CD3ε Nb‐EVs and recombinant CD3ε Nb proteins were trypsinized in 7.5 mL of 1% CHAPS (Millipore Sigma) for 1 h at 4°C, then subjected to high‐resolution/high‐accuracy liquid chromatograph/mass spectrometer (LC–MS/MS) (Lumos Fusion, Thermo Fisher Scientific). MS/MS was operated at resolution settings of 60 000 and 30 000. Only charge states 1, 2, and 3 were allowed. The isolation window was chosen as 1.6 Thomson, and the collision energy was set at 30%. The maximum injection time was set at 100–3500 ms with an automatic gain control of 50 000. The collected MS data were processed using Byonic software (version 2.7.84, Protein Metrics) through a custom‐built computer server equipped with four Intel Xeon E5‐4620 8‐core CPUs operating at 2.2 GHz and 512 GB physical memory (Exxact Corporation). The results of CD3ε‐Nb EV were searched against a database comprising the MS/MS sequencing data and the amino acid sequence of CD3ε Nb.

### 2.9 Surface Plasmon Resonance (SPR) Analysis

SPR experiments were performed using a Biacore T200 (Biacore‐GE Healthcare, Piscataway, NJ) with a CM5 sensor chip. The CM5 chip surface was activated using a standard EDC/NHS amine coupling protocol, consisting of a 1:1 mixture of 0.4 m EDC (1‐ethyl‐3‐(3‐dimethylaminopropyl) carbodiimide) and 0.1 m NHS (N‐hydroxysuccinimide). EV was diluted in 10 mm sodium acetate buffer (pH 4.5) and injected over the activated surface until the desired immobilization level (5000–8000 RU) was achieved. The remaining active esters were subsequently blocked with 1 m ethanolamine. Recombinant CD3ε proteins or antibodies (700, 350, 175, 87.5, 43.8, and 21.9 nm) were diluted in running buffer and injected over the EV‐coated flow cell to reach a final capture level of 300–700 RU. Kinetic analysis was performed by injecting serial dilutions of the indicated analytes over both the reference and EV‐captured flow cells at a constant flow rate of 30–50 µL/min. Binding responses were recorded in real time, and sensor grams were reference‐subtracted prior to analysis. After each binding cycle, the sensor surface was regenerated using a brief injection of low‐pH glycine buffer (pH 2–3). The data were analyzed by using the kinetic constants to determine the *K*_D_ values.

### 2.10 Electroporation With Nb‐CAR.TCE Transgene into EVs and Determination of the Encapsulated and Genomic DNA‐Integrated Transgene by Real‐Time Quantitative PCR and Capillary Electrophoresis

Nb‐CAR.TCE transgene was loaded into EVs by electroporation using the 4D‐Nucleofector platform (Lonza) according to the manufacturer's instructions. Briefly, the unmodified or CD3ε‐targeting EVs were resuspended in the P3 Primary Cell 4D‐Nucleofector Solution and the S1 Supplement buffer system at a ratio of 4.5: 1, then mixed with the Nb‐CAR.TCE transgene‐containing endotoxin‐free water at a ratio of 3 × 10^8^ EV particles: 2 µg DNA per 10 µL volume. The EVs/transgene‐containing solution was added into the 100 µL Nucleocuvette Vessel or the 1 mL Nucleocuvette Cartridge, and then the electroporation process was started using the program code as primary P3 cells/CM137. After recovering at 4°C for 2 h, the EVs/transgene‐containing solution was incubated with 1000 IU/mL DNase at 37°C for 15 min and immediately filtered through a 30‐kDa cut‐off membrane to remove unencapsulated Nb‐CAR.TCE transgene. The Nb‐CAR.TCE transgene‐loaded EVs were resuspended in PBS, and the amounts of encapsulated Nb‐CAR.TCE transgene was quantified by real‐time PCR analysis, while the synthesized cDNAs were mixed with 2X SYBR Green PCR Master Mix (Applied Biosystems) and a pair of gene‐specific primers (forward: 5′‐CACCACACCAGCTCCTAGAC‐3′, and reverse: 5′‐AGTCCTCTTGTATGCACGGC‐3′). Regarding detecting the presence of Nb‐CAR.TCE transgene in genomic DNA obtained from PBMCs and xenografted COLO 205 tumor samples. The genomic DNA was extracted through Genomic DNA Extraction Kits (Thermo Fisher Scientific) according to the user instructions. The isolated genomic DNA (25 pg each sample) was mixed with primers specific to Nb‐CDAR.BiTE (forward: 5′‐CGGCGACGTGGAGGAG‐3′, and reverse: 5′‐AGCTCAGCAGCTGCATTCT‐3′) and reporter sequence: CCCCGGACCAATGTAC. The mixtures were subjected to real‐time PCR quantification using an ABI 7300 Real‐Time PCR System (Applied Biosystems). All reactions were performed in quadruplets. The serial dilutions of Nb‐CAR.TCE transgene products were used as the standards to quantify the encapsulated and genomic‐integrated transgene in each sample using the comparative CT method. The formation of Nb‐CAR.TCE transgene aggregates were measured by capillary electrophoresis using the Agilent 4200 TapeStation System. Briefly, Nb‐CAR.TCE transgene‐loaded EVs (5 × 10^7^ particles in 1–2 µL) were injected into the capillary without heating up for capillary electrophoresis. The electropherogram and the peak area of Nb‐CAR.TCE in EVs and the average peak area were determined and compared to those of the control (Nb‐CAR.TCE transgene only).

### 2.11 Azidation of CD3ε Nb

Azide‐PEG4‐NHS (Thermo Fisher Scientific) (10 mm, 4 equiv.) and recombinant CD3ε Nb (1 mg/mL, 1 equiv.) were incubated in 20 mm Na2HPO4/NaH2PO4 buffer (pH 8.5) at 25°C for 1 h and then at 4°C overnight. Free azide‐PEG4‐NHS was removed by filtration with a 3 kDa cut‐off membrane and then reconstituted with PBS at 1 mg/mL protein concentration determined by the BCA method.

### 2.12 Generation of CD3ε Nb‐Conjugated Liposomes and LNPs

Nb‐CAR.TCE transgene was encapsulated into liposomes using Lipofectamine 3000 (Thermo Fisher Scientific) or LNPs using GenVoy‐ILM T cell kit (Precision NanoSystems), combining DBCO‐PEG‐DSPE (1%). Briefly, the Nb‐CAR.TCE transgene was diluted in 10× formulation buffer and RNase‐free water at 1 mg/mL. Seventy‐two microliters of dilution buffer, 24 µL Nb‐CAR.TCE dilution, 24 µL lipid mix (provided with the kit), and 1.2 µL DBCO‐PEG‐DSPE (Sigma‐Aldrich) were microfluidically mixed using setting #5 of the NanoAssemblr Spark nanoparticle formulation system (Precision NanoSystems). The formulated Nb‐CAR.TCE DNA‐liposomes (or LNPs) were then incubated with azidated CD3ε Nb for 2 h at 4°C in 20 mm malate buffer (pH 4.0), then filtered through a 100 kDa cut‐off membrane to remove unconjugated Nbs and reconstituted in PBS. For characterization, the size diameter of the generated Nb‐CAR.TCE transgene‐liposomes or LNPs were determined by NTA analysis using ZetaView. For the determination of the Nb‐CAR.TCE encapsulation efficacy, the Nb‐CAR.TCE transgene‐liposomes/LNPs (3 × 10^8^ particles) were mixed with 0.1% Triton X‐100‐containing PBS and determined by qPCR analysis using specific primers. The conjugated efficiency of CD3ε Nb on liposomes and LNPs was measured by flow cytometry analysis and dot plot using anti‐VHH antibody.

### 2.13 Fluorescence‐Activated Cell Sorting (FACS) Analysis for Nb‐CAR Expression and Memory Status

Briefly, the samples (isolated splenocytes from mice or EVs‐treated PBMCs, CD3^+^, CD4^+,^ and CD8^+^ cells) were stained with fluorophore‐labeled antibodies against VHH, CD3, CD4, CD8, CD56, CD66b, CD14, CD19, CD27, CD45RA, CD95, TCRγδ, and TCRαβ, respectively, for 45 min on ice. After washing twice using 1% BSA containing PBS, the expression levels of Nb‐CAR on CD4^+^ T (CD3^+^/CD4^+^), CD8^+^ T (CD3^+^/CD8^+^), NK cells (CD56^+^), monocytes (CD3^‒^/CD14^+^), B cells (CD3^‒^/CD19^+^), neutrophils (CD3^‒^/CD66b^+^) were determined by flow cytometry (SONY, SA3800), and intracellular cytokine staining was performed to stain the presence of BiTE in CD4^+^ and CD8^+^ T cells. The gating strategy for determining the TCM (T central memory), TEM (T effector memory), naive, TTE (Terminal effector memory), and TSCM (T stem cell memory) status on Nb‐CAR‐positive CD4^+^, CD8^+^, or CD4^+^/CD8^+^ DP‐T cells was as follows: VHH^+^ cells were the first included, followed by CD4^+^ or CD8^+^. CD27^+^/CD45RA^‒^ cells were identified as TCM cells; CD27^−^/CD45RA^−^ cells as TEM cells; CD27^+^/CD45RA^+^ cells as naive T cells; and the CD27^+^/CD45RA^+^/CD95^+^ cells were recognized as TSCM cells; and the CD27^+^/CD45RA^+^/CD103^+^ cells were recognized as TRM (tissue‐resident memory) cells. TRM in MAIT cells were gated from CD45RA^+^/CD103^+^ cells on VHH^+^/CD3^+^/CD161^+^/TCR Vα7.2^+^ cells.

### 2.14 Isolation, Depletion, and/or Expansion of CD3^+^, CD4^+^, CD8^+^, CD4^+^CD8^+^ DP, Treg, and MAIT Cells From PBMCs and Tumor Tissues

Regarding the isolation of human CD3^+^, CD4^+,^ and CD8^+^ cells from PBMCs, EasySep Human CD3^+^, CD4^+,^ and CD8^+^ T Cell Isolation Kits (#17951, #17952, and #17953, respectively; STEMCELL Technologies) were performed according to the manufacturer's instructions. Briefly, 5 × 10^7^ PBMCs were resuspended in 2 mL PBS with 1% FBS and then subjected to a 50 µL Isolation Cocktail incubated at 25°C for 5 min, followed by mixing with 50 µL of magnetic particles. The cells were then placed into the EasySep Magnet (STEMCELL Technologies) and stood for 3 min. Finally, the enriched cell suspensions were collected into new tubes. For depletion of CD4^+^ and CD8^+^ cells within PBMCs, EasySep Human CD4^+^ and CD8^+^ T Cell Positive Selection Kits (#17851 and #17853, respectively). STEMCELL Technologies were performed according to the manufacturer's instructions, respectively. In brief, PBMCs (1 × 10^8^ in 2 mL PBS with 1% FBS) were incubated with 50 µL CD4^+^ or CD8^+^ tetrameric antibody complexes for 5 min before adding 100 µL magnetic particles. After incubation for 3 min, the CD3, CD4, or CD8‐depleted suspensions were transferred into new tubes. These steps were repeated two more times. The frequencies of cell populations were examined by flow cytometry using antibodies against CD3, CD4, CD8, CD14, CD19, CD56, CD66b, and TCRγδ, respectively. Finally, the isolated CD3^+^, CD4^+,^ or CD8^+^ cells, or the CD3, CD4, or CD8‐depleted PBMCs were subjected to subsequent experiments.

For the expansion of αβT cells, CD3^+^ cells isolated from PBMCs were incubated with ImmunoCult Human CD3/CD28 T Cell Activator kit (STEMCELL Technologies, #100‐0785) at the ratio as 1 × 10^6^ cells to 25 µL of CD3/CD28 T Cell Activator reagent supplement with IL‐2 (50 IU/mL) and IL‐15 (2 ng/mL) according to the instructions. After 14 days, the purity of αβT cells was measured by flow cytometry analysis using CD3, TCRαβ, and TCRγδ‐specific antibodies.

The isolation of human CD3^+^ TILs from COLO 205 and SKOV3 tumors was performed by Tumor Dissociation Kit (Miltenyi Biotec, #130‐095‐929) according to the user's instructions. Briefly, the tumor samples (2–4 mm) were added to the gentleMACS C Tube (Miltenyi Biotec) containing the enzyme mix (provided by the Tumor Dissociation Kit). Then the tubes were placed on the gentleMACS Dissociator (Miltenyi Biotec) with the program code 37C_h_TDK_1 for 50 min. The cell suspensions were then filtered through a 70 µm cell strainer and incubated with 5 mL RBC lysis buffer. After washing, the cell pellets were subjected to subsequent experiments.

For the isolation of Treg cells, the Cloudz Human Treg Expansion Kit was performed (R&D Systems, #CLD006) according to the user manual. Brief, PBMC‐derived CD4+ cells (5 × 10^5^) were mixed with 75 µL of 1× Cloudz Treg CD3/CD28 reagent and refreshed with media every 3 days for a total of 9 days. The frequencies of Treg cells were examined by flow cytometry using antibodies against CD4, CD25, and Foxp3, respectively. Finally, the enriched Treg cells were subjected to subsequent experiments.

For the isolation of CD4^+^CD8^+^ DP cells, PBMC‐derived CD4^+^ cells (1 × 10^7^) were incubated with 50 µL CD8^+^ tetrameric antibody complexes from the EasySep Human CD8^+^ T Cell Positive Selection Kits for 5 min. The CD4^+^CD8^+^ DP cell frequency was measured by flow cytometry using antibodies against CD4 and CD8 and then subjected to subsequent experiments.

MAIT cells were isolated from PBMCs by using streptavidin‐conjugated magnetic beads and biotin‐conjugated anti‐human CD161 and TCR Vα7.2 antibodies (Biolegend, clone HP‐3G10 and 3C10, respectively). In brief, isolated CD3^+^ cells from PBMCs (1 × 10^7^) were incubated with CD161 and TCR Vα7.2 antibodies (50 µL each, 1:50 dilution) for 15 min, then these cells were incubated with 50 µL of streptavidin‐conjugated magnetic beads. These cells were isolated after 5 min incubation using EasySep Magnet, and the frequency of MAIT cells was determined by flow cytometry using specific antibodies against CD3, CD161, and TCR Vα7.2. The MAIT cells were then expanded by using the ImmunoCult Human CD3/CD28 T Cell Activator kit, and the expanded cells were subjected to subsequent experiments.

### 2.15 CD3ε‐Nb Lentiviral Particle Production and Transduction

The Nb‐CAR.TCE plasmid, pVSV‐G or pCD3ε Nb‐VSG‐G, and psPAX2 were transfected into HEK293T cells at a ratio of 5:3:1 using Lipofectamine 3000 (Invitrogen). After 48 h, the supernatant was collected and concentrated through a 100 kDa cut‐off membrane, then reconstituted in PBS. The viral particles were determined by NTA analysis, VHH and VSG‐G expression levels were measured by dot plot using specific antibodies, and the binding affinity to recombinant protein CD3ε was determined by ELISA‐based assay.

Subsequently, the primary αβT cells (1 × 10^6^) were resuspended in Opti‐MEM (500 µL) and then added to the virus‐containing medium with 10 µg/mL protamine sulfate in a total volume of 1 mL. The following day, the medium was replaced with X‐VIVO15 medium supplement with 10% FBS. After three days, the transduction rate was determined by flow cytometry using specific antibodies against CD3, TCRαβ, and VHH. The transducing units (TU) of CD3ε‐Nb LVs in primary T cells were determined by the VHH‐expressing cell counts and subjected to an animal study.

### 2.16 ELISA Assays, Multiplex Cytokine Analysis, and ELISA‐Based Mouse IgM/IgG Measurement

The levels of human IL‐6 (#HS600C), mouse IL‐6 (#M6000B), human IFN‐γ (#DIF50C), mouse C1q (#MCD930), mouse IL‐6, (#MFNAS0), mouse IFN‐α (#MIF00), mouse IFN‐γ (#MIF00), human TNF‐α (#DTA00D), mouse TNF‐α (#MTA00B), human IL‐1β (#HSLB00D), and mouse IL‐1β (#MHSLB00) were measured, along with the multiplex cytokine array set (which includes human CCL3/MIP‐1α, CCL4/MIP‐1β, CD25/IL‐2Rα, GM‐CSF, IFN‐γ, IL‐1β, IL‐2, IL‐4, IL‐5, IL‐6, IL‐8/CXCL8, IL‐10, IL‐12/IL‐23 p40, IL‐17/IL‐17A, and TNF‐α), in the serum samples and coculture supernatants using ELISA kits (R&D Systems) and ABplex Human Cytokine 15‐Plex Assay Kit (RK05202, Abclonal), following the manufacturer's instructions.

Regarding the quantification of the contents of mouse serum IgM and IgG against EV, LV, liposome, and LNP components, 1 × 10^9^ particles of CD3ε Nb or NLuc‐expressing EVs, LVs, liposomes, and LNPs were coated on each well of a 96‐well plate for 2 h. After washing and blocking with 2% BSA‐containing PBST, mouse serum (dilution: 10^3^–10^9^) was added overnight at 4°C. These wells were incubated the next day with HRP‐conjugated anti‐mouse IgM or IgG (1:5000) for 2 h. After washing, 50 µL TMB substrate was added to each well, and the absorbance was measured by an ELISA reader. The purified mouse IgM (Thermo Fisher Scientific, Cat: 39‐50470‐65) and IgG (Thermo Fisher Scientific, Cat: 39‐50400‐65) were used as standards for calculating the amounts of IgM and IgG in the mouse serum.

### 2.17 Dot Plotting

EVs or LVs (2 × 10^9^ particles), liposomes or LNPs (1 × 10^9^ particles) were adjusted in 20 µL with PBS each and spotted on a nitrocellulose (NC) membrane (Bio‐Rad). The membrane was dried at 37°C in the oven before the blocking step. After blocking, the blots were incubated with primary antibodies at 4°C overnight, then stained with secondary antibodies for 2 h at room temperature. After washing, the blots were incubated with the enhanced chemiluminescence reagent (Millipore) and analyzed using ChemiDoc Imaging Systems (Bio‐Rad Hercules).

### 2.18 LIVE/DEAD Cell‐Mediated Cytotoxicity Assay

The induced cytotoxicity in the cocultured tumor cells was determined using the LIVE/DEAD Cell‐Mediated Cytotoxicity Assay kit (Thermo Fisher Scientific) according to the user instructions. Briefly, tumor cells were pre‐stained using green‐fluorescent calcein‐AM for 15 min at 37°C. After washing, target tumor cells were co‐cultured with or without the indicated ratios of effector cells for 72 h, followed by staining with red‐fluorescent propidium iodide (2 µg/mL). Specific lysis of target cells was determined using green‐fluorescent^+^/red‐fluorescent^+^ cells through flow cytometry analysis and normalized to the untreated control.

### 2.19 Mice

All in vivo experiments were performed on 6–8‐week‐old NSG‐comparable (female, 20–22 g), 7–8‐week‐old C3H and C57BL/6 (female, 22–25 g) mice, which were purchased from the National Laboratory Animal Center in Taiwan or BioLASCO Taiwan Co. Ltd. All animal experimental protocols were approved by the Committee for Animal Experiments in China Medical University Hospital (CMUIACUC‐2023‐092), and mice were housed at the same animal facility under specific pathogen‐free conditions according to the animal care guidelines from the Committee.

### 2.20 Cell Line‐Derived Xenograft PBMC‐Humanized NSG (CDX‐huPBMC‐NSG) and Cell‐Line Derived Murine Glioblastoma‐Immunocompetent Mouse Model

COLO 205‐luc cells (1 × 10^6^ in 100 µL PBS) were intraperitoneally (I.P.) injected into female NSG mice to establish a colorectal cancer (CRC) tumor model. For the orthotopic lung tumor model, H1975‐luc cells were resuspended in Matrigel (1 × 10^6^ cells/20 µL), followed by rapid percutaneous injection into the upper margin of the sixth intercostal rib on the right anterior axillary at a 5 mm depth. For the orthotopic ovarian CDX model, SKOV3‐luc cells were resuspended in Matrigel (1 × 10^6^ cells/15 µL) and then injected into the left peritoneal ovary. HLA‐G‐overexpressing GL261‐luc cells (2 × 10^5^ in 2 µL PBS) were injected intracranially for the orthotopic brain tumor model.

In the CDX‐huPBMC‐NSG model, after tumor cell implantation for 7 days, all mice were transplanted with parental, CD4^−^, CD8^‐,^ or CD4/CD8‐depleted PBMCs, or isolated CD4^+^, CD8^+^, or CD4^+^/CD8^+^ (1:1) cells (5 × 10^6^ in 100 µL PBS) through tail vein injection. Before the EV, liposome, or LNP treatments, all mice were randomized before allocation to experimental groups. On the next day, mice were treated with Nb‐CAR.TCE transgene (or Nb‐CAR(WT).TCE, Nb‐CAR, Nb‐CAR(WT), Nb‐BiTE) or HER2‐CAR transgene‐loaded unmodified EVs, CD3ε‐Nb EVs, CD3ε Nb‐conjugated liposomes or LNPs, or CD3ε‐Nb LVs (1 mg/kg of encapsulated transgene/100 µL PBS for each mouse) once a week for 4 weeks. Tumor growth was monitored by IVIS Spectrum In Vivo Imaging System (PerkinElmer) using the bioluminescent channel, and the number of mouse deaths determined the survival rate. The sample size was chosen based on sufficiency for statistical analysis to calculate specificity and sensitivity. We estimated that statistically meaningful differences between groups of mice would require at least 5 mice per experimental group and per independent experiment. Investigators were blinded during data collection for each mouse's tumor size and survival rates.

### 2.21 Toxicity Evaluation in EV‐Treated Mice

Female C3H mice (*n* = 3 or 4) were injected weekly with CD3ε‐Nb EVs (2, 6, or 20 × 10^10^ particles in 100 µL PBS) via the tail vein for 4 weeks. The conventional toxicity signs, including body weight, activity, and diarrhoea scores [25, 26], were monitored twice a week until the indicated day (35 days after the first infusion of EVs).

### 2.22 Biodistributions of NLuc and CD3ε‐Nb NLuc EVs

On day 7 after intraperitoneal implantation of COLO 205‐luc cells (1 × 10^6 ^cells), mice (*n* = 4) were tail vein infused with huPBMCs (5 × 10^6^). Seven days later, mice were tail vein infused with NLuc EVs or CD3ε‐Nb NLuc EVs (5 × 10^9^) particles. After 5 min, these mice were injected with 100 µL of Nano‐GloFluorofurimazine In Vivo Substrate (Promega) through the tail vein. After 15 min of infusion, the mice were sacrificed, and the presence of infused EVs in xenografted COLO 205‐luc tumors, brain, lung, heart, liver, kidney, spleen, pancreas, ovary, stomach, intestine, and colon was detected by the IVIS system through detecting the bioluminescent signals. The bioluminescence in each organ was normalized to its weight (per gram).

### 2.23 Mitochondrial Respiration Measurement

To determine the mitochondrial respiration in PBMCs after treatment with or without different Nb‐CAR‐based constructs, their oxygen consumption rate (OCR) and ATP production were determined by Seahorse XFe24 Extracellular Flux Analyzer (Agilent Technologies). Briefly, PBMCs were incubated with or without CD3ε‐Nb EVs, which were loaded with Nb‐CAR.TCE, Nb‐CAR(WT).TCE, Nb‐CAR, Nb‐CAR(WT), or Nb‐BiTE transgene, in a ratio of 1 × 10^6^ cells: 3 × 10^8 ^EV particles. After 3 days, we seeded 8 × 10^5^ cells/100 µL per well onto 24‐well microplates pre‐coated with Cell‐Tak Cell and Tissue Adhesive (BD Biosciences). After centrifugation at 500 × *g* for 15 min, the pre‐warmed Seahorse assay medium (400 µL) was added to each well and incubated at 37°C without CO_2_ for 1 h. Afterward, the plate was loaded into the Seahorse XFe24 Extracellular Flux Analyzer. The metabolic profile was determined using a sequential injection protocol. Baseline OCR was recorded over an initial 17‐minute period. Next, 1 µm Oligomycin was added to each well to block complex V, and measurements were recorded. After 25.5 min, 0.5 µm FCCP was added to each reaction to permeabilize the mitochondrial inner membranes to uncouple electron transportation from the oxidative phosphorylation system for another 25.5 min. Finally, each well was injected with 0.5 µm Rot/Antimycin A to inhibit complex III and thereby halt both electron flow and oxygen consumption. All experiments were performed in triplicate. Data analysis and ATP production calculations were conducted using the Seahorse XFe24 software (Agilent Technologies) according to the manufacturer's instructions.

### 2.24 Statistical Analyses

Animal experiments, including tumor growth rate, body weight, activity, and diarrhea scores, were presented as mean ± SEM and analyzed using one‐way ANOVA (Analysis of Variance) to compare the means across multiple groups. The survival rate was analyzed using the Kaplan–Meier estimate and log‐rank test. Statistical significance was set at a *p‐*value of 0.05. All in vitro experiments were independently repeated at least three times. The sample size in each treatment group in the in vivo experiments was at least five, and no sample was excluded. The data were analyzed using a Student's *t‐*test or paired Student's *t‐*test using SigmaPlot 14.0 or Prism 9.0.0. Statistical differences in variables, including cell frequencies, cell killing, and ELISA assay data, were assessed using Student's *t*‐test and paired Student's *t*‐test.

## 3 Results

### 3.1 Generation and Characterization of the CD3ε Nb‐Expressing EVs

**FIGURE 1:**
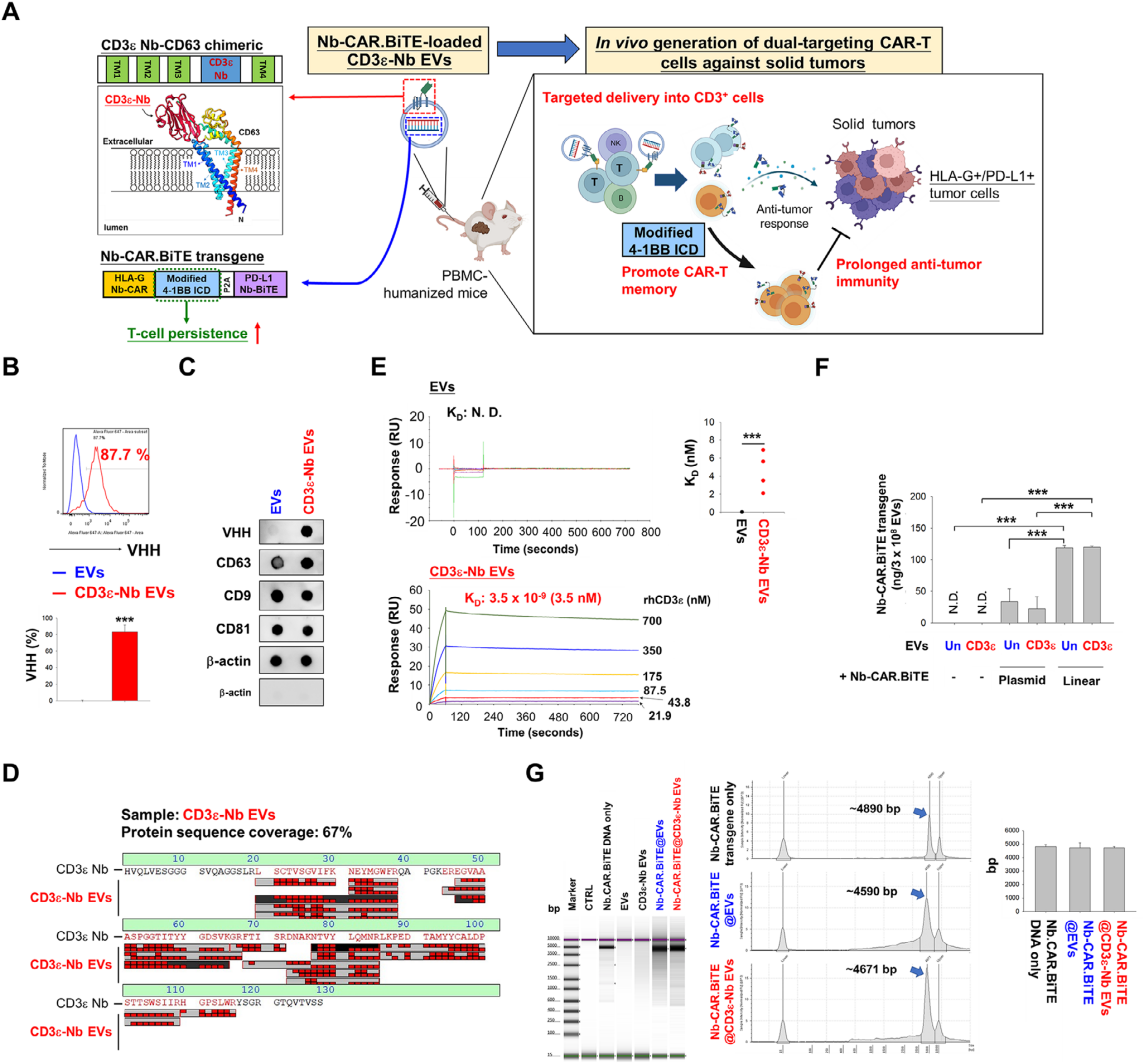
The characterization of the Nb‐CAR.TCE transgene‐loaded CD3ε Nb‐expressing EVs. (A) The diagrams show the construct of the CD3ε Nb‐CD63 chimeric protein. CD3ε Nb was inserted into the loop between the third and fourth transmembrane domains for CD63 (upper panel) and exposed outside the exosome membrane by modeling using the AlphaFold Protein Structure Database. This CD3ε Nb‐CD63 chimeric protein‐expressing EVs, derived from HEK‐293T cells, are subsequently loaded with linearized Nb‐CAR.TCE transgene. The Nb‐CAR.TCE transgene consists of a bicistronic HLA‐G‐targeted HLA‐G Nb‐CAR and a secretable PD‐L1‐targeted Nb‐BiTE (bottom left panel). These engineered EVs can deliver Nb‐CAR.TCE transgene into CD3‐positive T cells. The modified 4‐1BB ICD may promote memory CAR‐T formation and result in prolonged anti‐tumor activity in colorectal and lung cancer CDX‐PBMC huNGS mouse models (Right panel). (B) The purity of isolated CD3ε Nb‐expressing EVs. CD3ε Nb‐expressing EVs were harvested from the supernatants of CD3ε Nb‐CD63 stably expressing HEK‐293T cells by anti‐camelid VHH‐affinity resin. Then the expression levels of CD3ε Nb moiety on these EVs were determined by flow cytometry using a VHH‐specific antibody. (C,D) The presence of the CD3ε Nb components on the engineered EVs was validated by (C) detecting the expression levels of CD3ε Nb moiety and the EV markers through dot blotting using specific antibodies against VHH, CD63, CD9, CD81, TSG101, and β‐actin; and by (D) LC‐MS using recombinant CD3ε Nb protein as the reference spectrum. (E) Binding affinity of CD3ε‐Nb EVs. Unmodified and CD3ε‐Nb EVs (1 × 10^11^ particles) were coated on CM5 chips, and their binding affinity to recombinant CD3ε protein (700, 350, 175, 87.5, 43.8, 21.9 nm) was determined by SPR binding affinity assay. (F) HEK‐293 cell‐derived EVs were able to encapsulate the CAR transgene through electroporation. Unmodified and CD3ε Nb‐expressing EVs were respectively electroporated with Nb‐CAR.TCE‐linear or plasmid transgene at a ratio of 3 × 10^8^ EVs: 2 µg DNA using LONZA 4D‐Nucleofector. The EVs were then incubated with DNase (1000 IU) for 15 min, and the incorporated Nb‐CAR.TCE transgene was quantified by qPCR analysis using specific primers. (G) Absence of DNA aggregate formation in EVs electroporated with Nb‐CAR.TCE transgenes. Unmodified EVs and CD3ε‐Nb EVs loaded with or without Nb‐CAR.TCE transgenes were injected into the capillary (5 × 10^7^ particles in 1 µL) without heating for capillary electrophoresis using the Agilent 4200 TapeStation System. The electropherogram and the peak area of Nb‐CAR.TCE in EVs (left panel), as well as the average peak area (right panel), were determined and compared to those of the control (Nb‐CAR.TCE transgene only). Data are presented as mean ± SD, ***p < 0.001.

To establish a CD3 targeting EV platform for CAR transgene delivery, we developed a CD3ε Nb‐CD63 chimeric construct, which consists of the EV tetraspanin protein CD63, with a CD3ε Nb inserted into the extracellular loop between the third and fourth transmembrane domains (Figure 1A). This fusion construct was transfected into HEK‐293T cells, followed by screening to establish a stable clone. The CD3ε Nb‐expressing EVs generated by the stable clone were harvested from supernatants by a tangential flow filtration (TFF) system and purified through a VHH‐affinity resin column (Figure 1B). Both TFF and affinity purification are required to obtain high purity and adequate concentration of CD3ε‐Nb EVs appropriate for further transgene electroporation. Subsequently, EVs were loaded with Nb‐CAR.TCE transgene through electroporation and then isolated by 30‐kDa membrane filtration following depletion of unencapsulated transgene through DNase incubation (Figure S1B).

The VHH‐CD63 fusion protein was detected in the CD3ε‐Nb EV lysates. In contrast, the EV‐conserved proteins CD9, CD81, and TSG101 were comparable to unmodified EVs (Figure 1C). Notably, LC‐MS spectrum analysis identified CD3ε‐Nb derived peptide sequences in the digests of CD3ε‐Nb EVs (Figure 1D). The binding affinity of CD3ε‐Nb EVs to recombinant CD3ε protein was approximately 2.1 nm *K*_D_, as determined by surface plasmon resonance (SPR), while the binding affinity of unmodified EVs was undetectable (Figure 1E). Moreover, the small size of the linear Nb‐CAR.TCE transgene (∼4500 bp), which has higher transfection efficiency compared to the larger‐sized plasmid vector (∼9000 bp) (Figure 1F), was loaded into EVs through electroporation. As a result, both unmodified EVs and CD3ε‐Nb EVs were efficiently loaded with the linear transgenes at approximately 110 ng/3 × 10^8^ EVs (Figure 1F), with minimal detection of encapsulated DNA aggregation (Figure 1G). Furthermore, particle size (Figure S2A) and ultrastructure of EVs (Figure S2B) remained consistent after the loading process. In summary, we established HEK‐293T cell line‐derived CD3ε Nb‐engineered EVs carrying the dual‐targeting Nb‐CAR.TCE transgene (abbreviated as Nb‐CAR.TCE@CD3ε Nb EVs) through electroporation.

### 3.2 Selective Delivery of Nb‐CAR.TCE Transgene Into CD3^+^ Cells by CD3ε‐Nb EVs In Vitro and In Vivo

The targeted delivery of the Nb‐CAR.TCE transgene through the CD3ε‐Nb EVs platform was further evaluated. On days 3–7 following Nb‐CAR.TCE@CD3ε‐Nb EVs administration to PBMCs for transfection, the expression levels of Nb‐CAR on CD3^+^ cells were around 21.8%–24.9%, whereas the expression on CD3‐negative (CD3^−^) cells was limited. In contrast, the transfection efficiency mediated by unmodified EVs (EVs) was approximately 8% in CD3^+^ and 11% in CD3^−^ cells (Figure S3A). Next, the transfection efficiency and specificity of CD3ε‐Nb EVs for specific immune cell subtypes were further determined in human whole blood and PBMC‐humanized NOD scid gamma (PBMC‐huNSG) mice bearing COLO 205 cell line‐derived xenografts (COLO 205‐CDX‐huNSG mice for short), respectively. The results indicated that compared to unmodified EVs, CD3ε‐Nb EVs enhanced the transfection efficiency of the Nb‐CAR transgene into CD3^+^ subsets (CD4^+^, CD8^+^, and TCRγδ^+^ cells). In contrast, a reduction in transfection in CD3^−^ subsets (CD56^+^, CD14^+^, CD19^+^, and CD66b^+^ cells) was observed both in vitro and in vivo (Figures S3B and S4A,B).

Moreover, the transfection efficiency of Nb‐CAR.TCE transgene mediated by CD3ε‐Nb EVs was compared to that mediated by CD3ε‐Nb conjugated‐liposomes (CD3ε‐Nb liposomes), CD3ε‐Nb LNPs, and CD3ε‐Nb‐expressing lentiviral particles (CD3ε‐Nb LVs) (Figures S5, S6A, and S6B). Notably, comparable CD3‐targeting transfection efficiencies of Nb‐CAR.TCE transgene was observed between CD3ε‐Nb liposomes, CD3ε‐Nb LNPs, CD3ε‐Nb LVs, and CD3ε‐Nb EVs in a CDX‐huNSG mouse model (Figure S4C–F). Taken together, we propose that EVs show promise as a platform for transgene delivery, with CD3ε Nb‐engineered EVs enabling the selective delivery of the CAR transgene into CD3^+^ immune cells.

### 3.3 Effective Antitumor Activity of Nb‐CAR.TCE@CD3ε‐Nb EVs In Vitr*o* and In Vivo

The anticancer activity of immune cells after targeted delivery of Nb‐CAR.TCE transgene via CD3ε‐Nb EVs was evaluated. Solid tumor cell lines, including colorectal cancer (CRC) HCT‐116 and COLO 205 cell lines, pancreatic cancer AsPC‐1 and Panc‐1 cell lines, glioblastoma (GBM) GBM8901 and DBTRG‐05MG cell lines, and non‐small cell lung cancer (NSCLC) H1975 and A549 cell lines, were used as the in vitro models. The expression levels of the Nb‐CAR specific target, HLA‐G, and the Nb‐BiTE specific target, PD‐L1, on tumor cells were evaluated (Figure S7A). Nb‐CAR.TCE transgene transfection via CD3ε‐Nb EVs significantly enhanced the cytolytic capability of PBMCs against multiple solid tumor cell lines, surpassing the efficacy observed with unmodified EVs (Figure S7B). In contrast, HLA‐G KO or PD‐L1 KO reduced the cytotoxicity in A549 cells, while HLA‐G/PD‐L1 DKO diminished the cytotoxic killing ability enhanced by Nb‐CAR.TCE@CD3ε‐Nb EVs (Figure S7C,D). These findings were consistent with our previous study [8].

**FIGURE 2:**
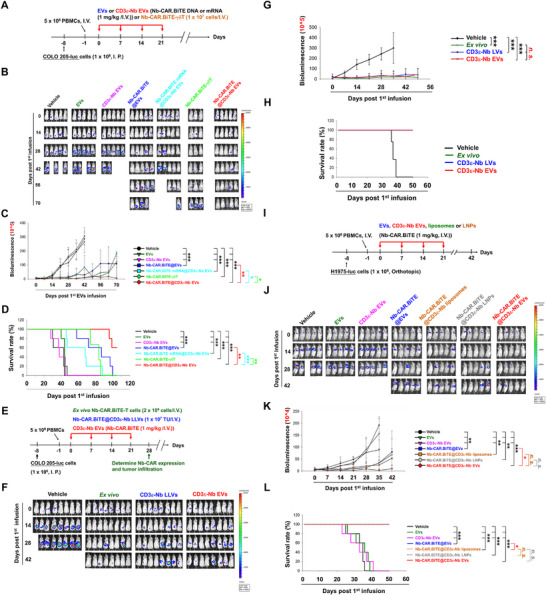
The anti‐tumor activity of Nb‐CAR.TCE@CD3ε‐Nb EVs in vivo. (A–D) Nb‐CAR.TCE@CD3ε‐Nb EVs elicited superior anti‐tumor activity against colon cancer in vivo. (A) Schematic representation of the protocol for an in vivo anti‐tumor efficacy study of Nb‐CAR.TCE@CD3ε‐Nb EVs. On day 7 after intraperitoneal implantation of luciferase‐expressing COLO 205 cells (1 × 10^6^ cells), mice (*n* = 5) were infused through the tail vein with huPBMCs (5 × 10^6^). The next day, mice were tail vein infused with or without unmodified or CD3ε‐Nb EVs encapsulated with or without 1 mg of Nb‐CAR.TCE transgene once a week for four weeks. (E–L) The comparison of anti‐tumor effect among Nb‐CAR.TCE‐loaded CD3ε‐Nb EVs, liposomes, LNPs, and LVs, and ex vivo manufactured Nb‐CAR.TCE‐T cells in vivo. (E,I) Schematic representation of the protocol for in vivo anti‐tumor efficacy of CD3ε‐Nb EVs, liposomes, LNPs, and LVs loading with Nb‐CAR.TCE transgene, and ex vivo manufactured Nb‐CAR.TCE‐T cells. After 7 days of orthotopic implantation with luciferase‐expressing H1975 or COLO 205 cells (1 × 10^6^ cells), the mice (*n* = 5 and 8, respectively) were tail vein injected with huPBMCs (5 × 10^6^). The next day, the mice were infused with or without unmodified or CD3ε‐Nb EVs, CD3ε Nb‐conjugated liposomes, or LNPs encapsulated with 1 mg/kg of Nb‐CAR.TCE or Nb‐BiTE transgene, Nb‐CAR.TCE‐T cells (2 × 10^6^ cells) or Nb‐CAR.TCE@CD3ε‐Nb LVs (1 × 10^7^ TU) once a week for four weeks. The tumor growth was monitored weekly by the IVIS system through detecting the bioluminescent signals (B,C,F,G,J,K), and the survival rate of these mice was recorded (D,H,L). The in vivo tumor growth data were presented as mean ± SEM and analyzed using one‐way ANOVA for comparing means across multiple groups. The survival rate was analyzed based on the Kaplan–Meier method and log‐rank test. Statistical significance was set at *p*‐value < 0.05. *p < 0.05, **p < 0.01, ***p < 0.001.

Subsequently, CDX‐huNSG mice were employed to evaluate the antitumor efficacy of the in vivo CAR‐T platform, following the literature [9]. The experimental protocol is detailed in Figure 2A,E,I. Compared to Nb‐CAR.TCE@EVs, Nb‐CAR.TCE mRNA@ CD3ε‐Nb EVs (CD3ε‐Nb EVs encapsulated with mRNA), and ex vivo Nb‐CAR.TCE‐γδT, infusion of Nb‐CAR.TCE@CD3ε‐Nb EVs exhibited the most effective tumor growth suppression and prolonged the survival of mice inoculated with COLO 205 cancer cells (Figure 2B–D). The comparison of anti‐tumor activities of Nb‐CAR.TCE@CD3ε‐Nb EVs to those of the conventional ex vivo Nb‐CAR.TCE‐T cells and lentiviral vector (LV)‐based in vivo Nb‐CAR.TCE‐T cells were also conducted in COLO 205 CDX‐huNSG mice, showing comparable anti‐tumor effects (Figure 2E–H). In addition, the dose‐response of Nb‐CAR.TCE@CD3ε‐Nb EVs was evaluated in a COLO205 tumor‐bearing PBMC‐humanized NSG mouse model (Figure S8).

**FIGURE 3:**
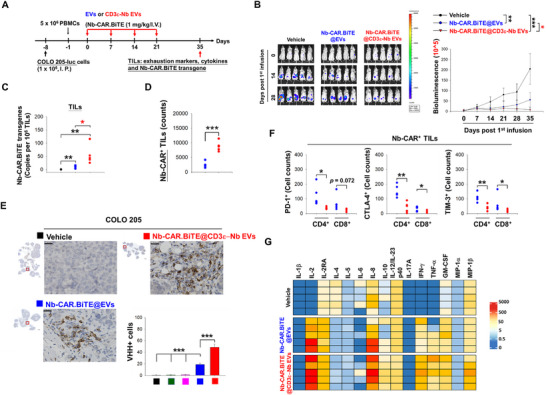
CD3ε‐targeting delivery of Nb‐CAR.TCE transgene promoted T‐cell tumor infiltration and persistence in vivo. (A) Schematic representation of the protocol for evaluating the persistence of CD3ε‐Nb EV‐mediated in vivo generated Nb‐CAR.TCE‐T cells. Seven days after intraperitoneal implantation of luciferase‐expressing COLO 205 cells (1 × 10^6^ cells), mice (*n* = 5) were infused with huPBMCs (5 × 10^6^) through the tail vein. The next day, mice were treated with unmodified or CD3ε‐Nb EVs encapsulated with 1 mg of Nb‐CAR.TCE transgene via tail vein injection weekly for four weeks. (B) The tumor growth was monitored weekly by detecting bioluminescent signals through the IVIS system. All mice were sacrificed on day 35 post the first EV infusion. The TILs were collected, and (C) detected the presence of Nb‐CAR.TCE transgene in their genomic DNA by qPCR using specific Taqman primers. (D,E) The tumor‐infiltrated VHH‐expressing cells were counted and analyzed by flow cytometry (D) and IHC staining (E) using specific antibodies against CD3 and VHH. (F) The frequencies of PD‐1, CTLA‐4, and TIM‐3‐expressing Nb‐CAR‐positive TILs were measured by flow cytometry analysis using specific antibodies. (G) Or the isolated TILs (*n* = 5) were challenged with recombinant HLA‐G (1 µg/mL) for 24 h, and the secreted cytokines were detected by ABplex Human Cytokine 15‐Plex Assay Kit according the user instructions. The in vivo tumor growth data were presented as mean ± SEM and analyzed using one‐way ANOVA to compare means across multiple groups. Statistical significance is set at *p*‐value < 0.05. *p < 0.05, **p < 0.01, ***p < 0.001.

Moreover, CD3ε‐Nb EVs‐mediated in vivo CAR T (Nb‐CAR.TCE@CD3ε‐Nb EVs) showed comparable anti‐tumor activities to CD3ε Nb‐conjugated liposomes‐mediated (Nb‐CAR.TCE@CD3ε‐Nb liposomes) and CD3ε Nb‐conjugated LNPs‐mediated (Nb‐CAR.TCE@CD3ε‐Nb LNPs) in vivo CAR T therapy in H1975 CDX‐huNSG mice (Figure 2I–L), which was associated with similar tumor‐infiltration of Nb‐CAR‐expressing cells (Figure S8). Notably, compared with unmodified EVs, CD3ε‐Nb EV‐mediated in vivo CAR T therapy increased the content of Nb‐CAR.TCE transgenes in the genomic DNA oof TILs (Figure 3A–C), enhanced the amounts of tumor‐infiltrated VHH‐expressing cells, and reduced the number of α‐SMA+ (CAFs), Foxp3+ (Treg), and CD31+ (endothelium) cells, which were associated with a decrease in exhaustion markers PD‐1, CTLA‐4, and TIM‐3, and can induce the secretion of antitumor‐related polyfunctional cytokines in response to HLA‐G antigen challenging (Figure 3D–G; Figures S9 and S10). Furthermore, we demonstrated that the CD3ε‐Nb EVs can serve as a CD3‐targeted platform for delivering transgenes to edit T cells in vivo, successfully generating HER2 CAR‐T cells, which led to significant tumor suppression in the SKOV3 CDX‐huNSG mouse model (Figure S11).

### 3.4 Synergistic Mediation of Antitumor Efficiency by CD4^+^ and CD8^+^ Cells in Nb‐CAR.TCE@CD3ε‐Nb EVs Treatment

Based on the experimental findings, CD3ε‐Nb EVs selectively transfected the Nb‐CAR.TCE transgene into CD3^+^ cells, encompassing both CD4^+^ and CD8^+^ T cell subsets (Figures S3 and S4), thereby resulting in potent CAR‐T cytotoxicity against solid tumor cells both in vitro and in vivo (Figure 2; Figure S7). We subsequently identified the contribution of T cell subsets to the in vivo CAR‐T cytotoxicity driven by CD3ε‐Nb EVs.

The proportions of T cell subsets within PBMCs after depletion or isolation of CD3^+^, CD4^+^, and/or CD8^+^ cells were determined (Figure S12A,B). Depleting both CD4^+^ and CD8^+^ cells in PBMCs significantly diminished the enhanced antitumor activity induced by Nb‐CAR.TCE@CD3ε‐Nb EVs in a COLO 205‐CDX‐huNSG mouse model (Figure S13A–D). Following the depletion of CD3^+^ cells in PBMCs, the Nb‐CAR.TCE@CD3ε‐Nb EVs induced antitumor efficacy was significantly diminished, with mild attenuation observed in the individual CD4‐depletion and CD8‐depletion groups. Conversely, Nb‐CAR.TCE@CD3ε‐Nb EVs treatment exhibited the most excellent antitumor efficacy, effectively suppressing COLO 205 tumor growth and extending survival in NSG mice infused with intact PBMCs. Furthermore, Nb‐CAR.TCE@CD3ε‐Nb EVs infusions following engraftment with intact PBMCs demonstrated a significantly greater efficiency than simultaneous with CD4^+^ and CD8^+^ T cells, while both were superior to the implantation of CD4^+^ or CD8^+^ T cells alone (Figure S12E–H).

### 3.5 Association Between Memory CAR‐T Cells and Sustained Antitumor Immunity Elicited by Nb‐CAR.TCE@CD3ε‐Nb EVs

**FIGURE 4:**
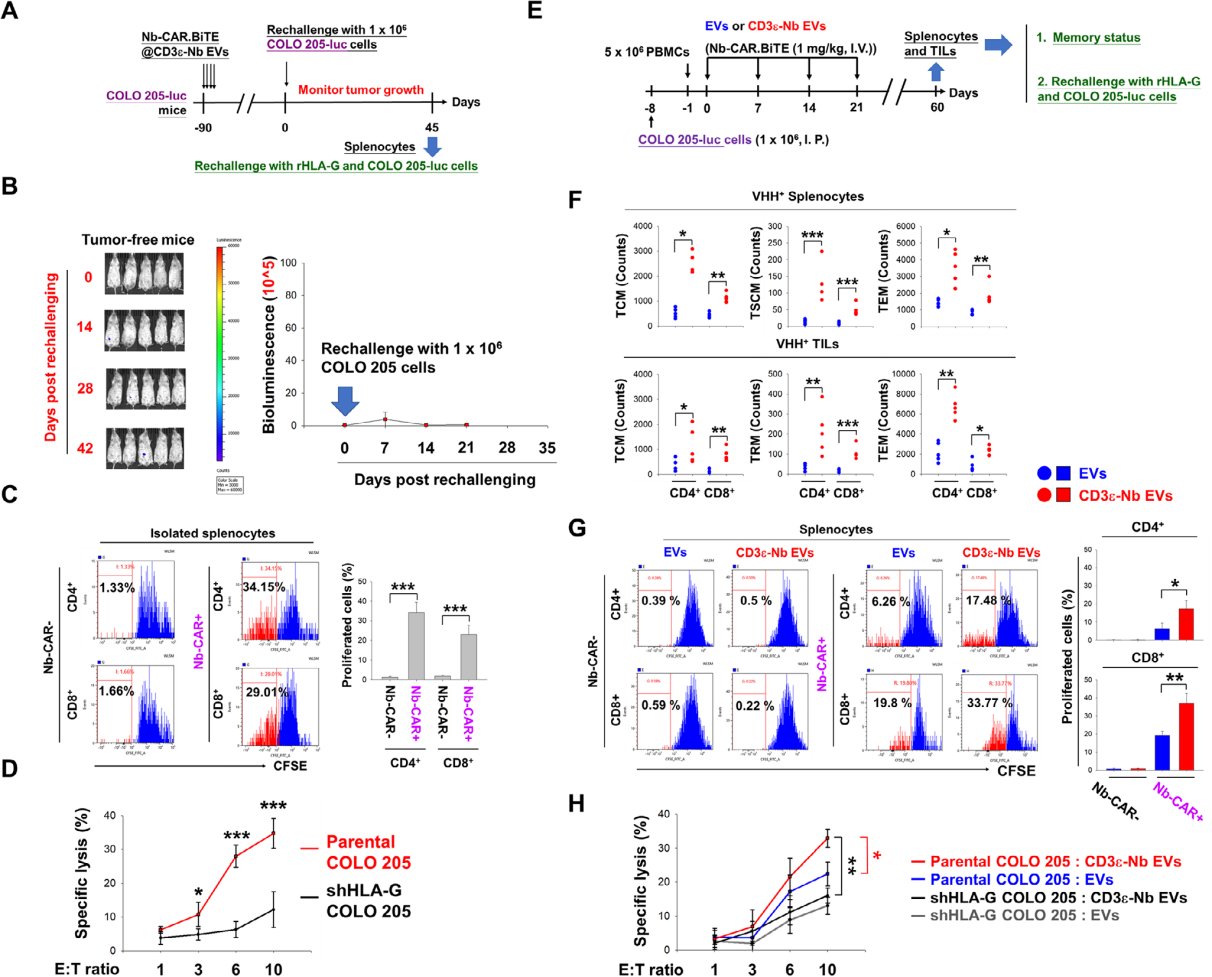
Targeted delivery of Nb‐CAR.TCE transgene by CD3ε‐Nb EVs increased the proportion of memory CAR‐T cells and elicited long‐term antitumor activity. (A–D) Nb‐CAR.TCE@CD3ε‐Nb EVs promoted long‐term tumor surveillance in vivo. (A) The schematic diagram illustrates the in vivo experimental protocol. COLO 205‐Luc‐CDX‐PBMC‐humanized mice that received Nb‐CAR.TCE@CD3ε‐Nb EVs treatment and achieved tumor‐free outcomes (*n* = 5) were rechallenged with COLO 205‐Luc cell implants (1 × 10^6^ cells/I.P.) on day 90 after the initial injection of EVs. Afterward, the tumor growth rate was monitored (B). After being rechallenged with COLO 205‐luc cells for 45 days, all mice were sacrificed, and their splenocytes (2 × 10^5^) were isolated, followed by (C) stimulation with recombinant HLA‐G protein (1 µg/mL) for 7 days. Then the expansion of CD4^+^ and CD8^+^ splenocytes with Nb‐CAR‐positive or negative expression was measured by CFSE staining using flow cytometry analysis, or (D) cocultured with parental or HLA‐G‐knockdown (shHLA‐G) COLO 205‐luc cells with the E: T ratios of 1:1, 3:1, 6:1, and 10:1 for 72 h. The induced cytotoxicity was then determined by LIVE/DEAD Cell‐Mediated Cytotoxicity Assay using flow cytometry analysis. (E–H) Targeting‐delivery of Nb‐CAR.TCE transgene by CD3ε‐Nb EVs increased the memory CAR‐T fraction and responded to tumor antigen rechallenge. (E) Representative protocol for investigating the memory T proportion and antigen reactivity of in vivo Nb‐CAR.TCE‐T cells generated by CD3ε‐targeting EV‐mediated transgene delivery. Seven days after intraperitoneal injection with 1 × 10^6^ COLO 205‐luc cells, mice were injected with 5 × 10^6^ PBMCs via the tail vein. Subsequently, they were infused with either unmodified or CD3ε‐Nb EVs encapsulated with Nb‐CAR.TCE transgene (1 mg/kg) on the following day, repeating once a week for four weeks. On day 60 following the initial infusion of EVs, mice were sacrificed, and their (F) TILs and splenocytes were subjected to analysis for the cell counts and phenotypic markers of naïve, TCM, TSCM, TRM, TEM and TTE in Nb‐CAR‐expressing CD4^+^ and CD8^+^ cells by flow cytometry analysis using specific antibodies against VHH, CD4, CD8, CD27, CD45RA, CD95 and CD103 (gating strategy is presented in Figure S6C,D); or the splenocytes were stimulated with 1 µg/ml recombinant HLA‐G for 7 days, and then subjected to cell proliferation assay, which was determined by CFSE staining and flow cytometry analysis (G); or co‐incubated with either parental or shHLA‐G COLO 205‐luc cells as E: T ratios of 1:1, 3:1, 6:1, and 10:1 for 72 h. The induced cytolysis was then analyzed using the LIVE/DEAD Cell‐Mediated Cytotoxicity Assay (H). Data are presented as mean ± SD and analyzed by Student's *t*‐tests and Paired *t*‐tests. Statistical significance is set at *p*‐value < 0.05. *p < 0.05, **p < 0.01, ***p < 0.001.

Complete elimination of implanted tumors was observed in certain COLO 205 and H1975 tumor‐bearing mice after treatment with Nb‐CAR.TCE@CD3ε‐Nb EVs (Figure 2B,C,K,L; Figure S12B,C,F,G). To evaluate whether the treatment of Nb‐CAR.TCE@CD3ε‐Nb EVs induced long‐term anti‐tumor immunity, mice whose tumors had been eliminated were re‐implanted with the same tumor cells (Figure 4A). Notably, the rechallenged COLO 205 cells were still eradicated in these mice without additional treatment (Figure 4B). Furthermore, Nb‐CAR‐expressing CD4^+^ and CD8^+^ splenocytes, isolated from these mice, responded to recombinant HLA‐G, resulting in clonal expansion (Figure 4C). Additionally, these Nb‐CAR‐expressing splenocytes demonstrated higher cytotoxicity against parental COLO 205 cells than against HLA‐G‐knockdown cells (Figure 4D).

To further investigate whether the CD3ε‐targeting EV platform influences memory CAR‐T proportion, tumor‐infiltrated leukocytes (TILs) and splenocytes were isolated from the COLO 205‐CDX‐huNSG mice treated with Nb‐CAR.TCE@CD3ε‐Nb EVs and Nb‐CAR.TCE@EVs, respectively (Figure 4E). Specifically, increased cell counts of T central memory (TCM; CD27^+^/CD45RA^‒^) and T stem cell memory (TSCM; CD27^+^/CD45RA^+^/CD95^+^) cells; TCM and T tissue‐resident memory cells (TRM; CD27^+^/CD45RA^+^/CD103^+^) were demonstrated within Nb‐CAR‐expressing CD4^+^ and CD8^+^ splenocytes and TILs, respectively (Figure 4F; Figure S10C,D). Consistently, the proliferation of Nb‐CAR‐positive CD4^+^ and CD8^+^ splenocytes after stimulation with recombinant HLA‐G was significantly greater in Nb‐CAR.TCE@CD3ε‐Nb EVs treatment group (Figure 4G). Furthermore, splenocytes isolated from mice treated with Nb‐CAR.TCE@CD3ε‐Nb EVs exhibited significantly higher cytotoxicity against COLO 205 cells than those from mice treated with unmodified EVs (Figure 4H). Similarly, CD3ε‐targeted delivery of the HER2‐CAR transgene also increased the proportions of TCM and TSCM (or TRM) phenotypes in splenic cells and TILs in the SKOV3 CDX‐huNSG mouse model (Figure S11E).

Additionally, to evaluate the Nb‐CAR.TCE transgene delivery in other T‐cell subsets, we enriched regulatory T (Treg), CD4^+^CD8^+^ double‐positive (DP), and mucosal‐associated invariant T (MAIT) cells from PBMCs after Nb‐CAR.TCE@CD3ε‐Nb EVs treatment (Figures S14A, S15A, and S16A, respectively). The results showed that the treatment of Nb‐CAR.TCE@CD3ε‐Nb EVs successfully delivered the Nb‐CAR.TCE transgene into Treg, CD4^+^CD8^+^ DP, and MAIT cells (Figures S14B, S15B, and S16B, respectively), resulting in enhanced cytotoxicity to COLO205 cells (Figures S14C, S15C, and S16C). Compared to unmodified EVs, CD3ε‐Nb EVs further enhanced the effector phenotype (CD45RA^lo^Foxp3^lo^) and reduced the suppressive phenotype (CD45RA^lo^Foxp3^hi^) (Figure S14D), accompanied by a decrease in CD44, PD‐1, and CTLA‐4 levels, even after engaged with COLO 205 tumor cells (Figure S14E). Moreover, Nb‐CAR.TCE@CD3ε‐Nb EVs increased the TCM frequency in CD4^+^CD8^+^ DP cells (Figure S15D) and TRM frequency in MAIT cells (Figure S16D). After coculturing with COLO 205 cells, the proportion of memory phenotype in CD4^+^CD8^+^ DP (CD27^hi^CD45RA^lo^) and MAIT (CD45RA^hi^CD103^hi^) subtypes remained higher (Figures S15D and S16D, respectively) in the Nb‐CAR.TCE@CD3ε‐Nb EVs treatment group, while the frequency of exhausted cells (PD‐1 and/or CTLA‐4‐positive) was lower than in Nb‐CAR.TCE@ EVs treatment group(Figures S15E and S16E).

In summary, CD3ε‐Nb EVs enhance the delivery of the Nb‐CAR.TCE transgene into T cells, supporting the development and persistence of TCM and TSCM subsets. This helps prevent T cell exhaustion, increases cytokine polyfunctionality, and shifts T cells, including rare subsets, toward effector phenotypes, resulting in a stronger anti‐tumor response even upon antigen rechallenge.

### 3.6 Nb‐CAR.TCE Construct Features Promote the Formation and Persistence of TCM and TSCM Subsets

**FIGURE 5:**
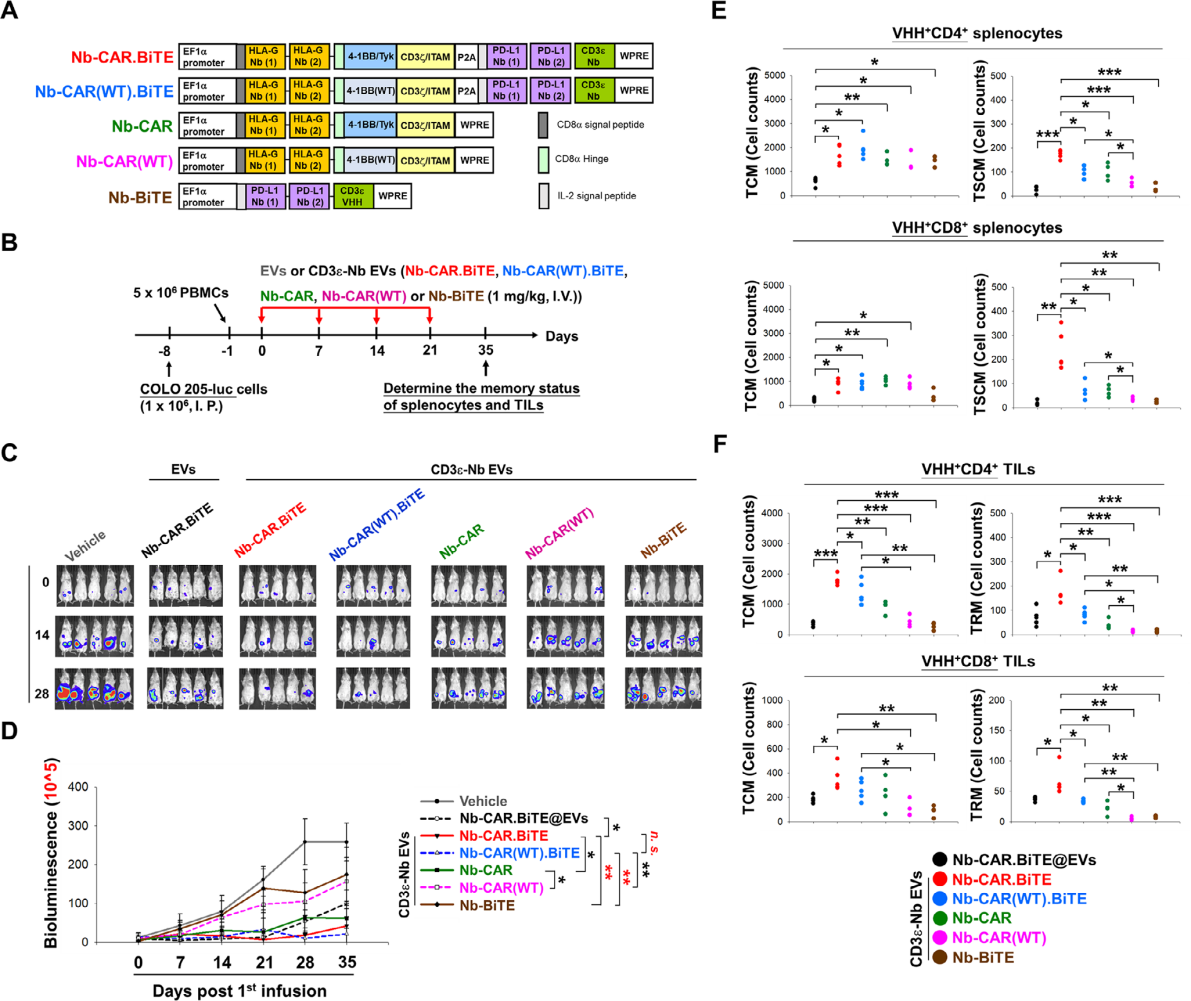
The characteristics of Nb‐CAR.TCE transgene contributed to the increased proportions of memory CAR‐T cells. (A) Schematic representation of different Nb‐CAR constructs with or without Nb‐BiTE and modified ICD. The bi‐epitopic Nb‐CAR comprises two tandem extracellular HLA‐G‐targeting Nbs fused with a CD8α hinge, followed by a modified 4‐1BB (4‐1BB/Tyk) or a wild‐type 4‐1BB (WT) ICD, and a modified CD3ξ ICD (CD3ξ/ITAM) linked to a self‐cleavage peptide P2A to separate the secretable bivalent Nb‐BiTE, which consists of two anti‐PD‐L1 Nbs linked to a CD3ɛ Nb. An EF‐1α promoter drives this construction and has a post‐transcriptional regulatory element (WPRE) at the 3′‐terminal. (B) Schematic representation of animal studies for evaluating the effect of different Nb‐CAR constructs with or without Nb‐BiTE and modified ICD on T cell memory status. Seven days after intraperitoneal implantation of luciferase‐expressing COLO 205 cells (1 × 10^6^ cells), all mice (*n* = 5) were transplanted with huPBMCs (5 × 10^6^) through tail vein injection. The next day, mice were injected with CD3ε‐Nb EVs, encapsulated with 1 mg/kg of Nb‐CAR.TCE, Nb‐CAR(WT).TCE, Nb‐CAR, Nb‐CAR(WT), or Nb‐BiTE transgenes, or injected with unmodified EVs encapsulated with 1 mg of Nb‐CAR.TCE via the tail vein once a week for four weeks. (C,D) The tumor growth was monitored weekly by detecting the bioluminescent signals with the IVIS system. (E,F) Fourteen days after the last infusion, mice were sacrificed, and the cell counts of TCM, TSCM, and TRM markers in VHH‐expressing CD4^+^ and CD8^+^ splenocytes (E) and TILs (F) were determined by flow cytometry analysis using specific antibodies against VHH, CD4, CD8, CD27, CD45RA, and CD103. The in vivo tumor growth data are presented as mean ± SEM and analyzed using one‐way ANOVA for comparing means across multiple groups. Data are presented as mean ± SD, *p < 0.05; **p < 0.05; ***p < 0.05. Student's *t‐*test and paired Student's *t*‐test.

It has been shown that 4‐1BB CAR constructs enhance TCM differentiation in CAR‐T cells [30, 31], and PD‐L1/PD‐1 axis blockade promotes memory T cell differentiation [32]. Additionally, the insertion of the Tyk2‐binding motif into the 4‐1BB ICD may extend transgene persistence and potentially influence T cell memory status [33, 34]. Accordingly, we assessed the effects of the Tyk2‐binding motif‐modified 4‐1BB ICD and the secretable Nb‐BiTE on TCM/TSCM differentiation in Nb‐CAR‐positive T cells (Figure 5A,B). The antitumor activity of these CAR transgene‐loaded CD3ε‐Nb EVs was monitored (Figure 5C,D). The targeted delivery of 4‐1BB‐based CAR by CD3ε‐Nb EVs increased the cell counts of both TCM and TSCM in Nb‐CAR‐expressing splenocytes and in both TCM and TRM in Nb‐CAR‐expressing TILs compared to unmodified EVs (Figure 5E,F). The incorporation of a Tyk2‐binding motif‐modified 4‐1BB ICD significantly enhanced the proportions of TSCM and TRM (red color vs. blue and green vs. pink; Figure 5E,F). The Nb‐BiTE motif further influenced TSCM and TRM differentiation in Nb‐CAR‐positive splenocytes and TRM in TILs, respectively (red vs. green and blue vs. pink; 5E and 5F).

It has been shown that a metabolic switch toward oxidative mitochondrial respiration is indispensable for CAR‐T cell memory formation [30]. In the present study, we found that the modified CAR ICD with a Tyk‐binding motif derived from IFNAR1 and the secretable PD‐L1‐targeted Nb‐BiTE may enhance CAR‐T‐cell memory. Additionally, we assessed downstream signaling pathways correlated with the modified CAR ICD and found that the presence of modified CAR ICD in the Nb‐CAR.TCE construct increased phosphorylation of Tyk2, STAT2, and Syk/ZAP70 in transfected CD3^+^ cells compared with the unmodified CAR ICD (Nb‐CAR(WT).TCE) group (Figure S17A), consistent with our previous study [8]. Furthermore, the modified CAR ICD enhanced mitochondrial OCR and ATP production compared with the unmodified CAR ICD group (Figure S17B,C).

Moreover, incorporating the secretable Nb‐BiTE significantly enhanced antitumor activity of Nb‐CAR both in vivo (Figure 5C,D; red vs. green and blue vs. pink) and in vitro (Figure S18A; red vs. green), while Nb‐BiTE was detectable in tumor extracts but barely detectable in serum (Figure S18A bottom panel and Figure S18B), consistent with our previous study (8). Notably, Nb‐BiTE was detected in the serum of the Nb‐BiTE@CD3ε‐Nb EVs treatment group (lacking the Nb‐CAR fragment), indicating that the Nb‐CAR endows immune cells with the ability to precisely target tumors, thereby allowing the secreted BiTE to concentrate on tumor lesions (Figure S18B, bottom panel).

In summary, the CD3ε‐targeting modification of EV may enhance the delivery of Nb‐CAR.TCE transgene into T cells, supporting the consequent differentiation of TCM and TSCM phenotypes associated with improved mitochondrial respiration, driven by the expression of the construct, which features a Tyk2‐binding motif within the 4‐1BB ICD and a secretable Nb‐BiTE moiety, thereby collectively promoting the formation and persistence of TCM and TSCM subsets.

### 3.7 Lower Immunogenic Risks of CD3ε‐Nb EVs Compared to Lipid‐Based Carriers for Transgene Delivery

**FIGURE 6:**
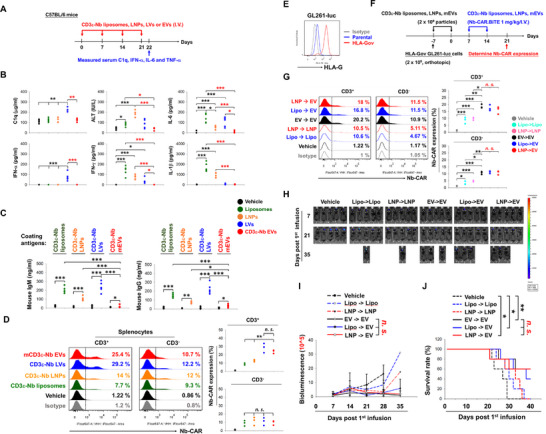
CD3ε‐Nb EVs showed higher transfection efficiency with minimal immunogenicity risk than lipid and viral‐carrier‐based approaches. (A) Schematic protocol for evaluating the immunological responses of CD3ε‐Nb mEVs, liposomes, LNPs, and LVs in an immunocompetent mouse model. C57BL/6 mice (*n* = 5) were treated with or without 2 × 10^9^ particles of CD3ε Nb‐conjugated liposomes, LNPs, NIH/3T3‐derived CD3ε Nb‐mouse CD63‐expressing EVs (CD3ε‐Nb mEVs), or CD3ε‐Nb LVs weekly for four weeks through tail vein injection. All mice were sacrificed, and their serum and splenocytes were collected. (B) The serum contents of C1q, ALT, IFN‐α, IL‐6, IFN‐γ, and IL‐1β were detected using ELISA kits according to the user instructions. (C) The presence of IgM and IgG against CD3ε Nb‐expressing liposomes, LNPs, LVs, or mEVs in the serum was determined by an ELISA‐based assay through coating with CD3ε‐Nb liposomes, LNPs, LVs, or mEVs. (D) The Nb‐CAR‐expressing splenocytes were determined by flow cytometry using specific antibodies against VHH and mouse CD3. (E–J) Minimal influence on EV‐mediated in viv*o* Nb‐CAR.TCE‐T generation by prior exposure of lipid‐based carriers. (E) The expression of HLA‐G on HLA‐G‐overexpressing GL261‐luc cells was measured by flow cytometry. (F) Representative protocol for investigating the effect of prior exposure with liposomes and LNPs on subsequent transgene delivery using CD3ε‐Nb mEVs in a tumor‐bearing syngeneic mouse model. C57BL/6 mice (*n* = 3 and 5) were orthotopically implanted with GL261 cells (2 × 10^5^ cells) and subsequently treated with 2 × 10^9^ particles of CD3ε Nb‐conjugated liposomes, LNPs, NIH/3T3‐derived CD3ε Nb‐mouse CD63‐expressing EVs (CD3ε‐Nb mEVs), or CD3ε‐Nb LVs weekly for two weeks through tail vein injection. The mice were then infused with CD3ε‐Nb liposomes, LNPs, or mEVs loaded with the Nb‐CAR.TCE transgene (1 mg/kg) in the following two weeks. (G) All mice were sacrificed on day 21 after the first infusion, and the expression of Nb‐CAR on splenocytes was measured by flow cytometry using VHH and mouse CD3‐specific antibodies (G). Or (H–J) the tumor growth was monitored weekly using the IVIS system to detect the bioluminescent signals (H,I), and the survival rates of these mice were documented (J). The in vivo tumor growth data were presented as mean ± SEM and analyzed using one‐way ANOVA to compare means across multiple groups. The survival rate was analyzed using the Kaplan–Meier method and log‐rank test. Statistical significance was set at *p*‐value < 0.05. *p < 0.05, **p < 0.01, ***p < 0.001. The results of the experiments were analyzed using one‐way ANOVA to compare means across multiple groups. Data are mean ± SD, *p < 0.05; ** *p* < 0.01; *** *p* < 0.001.

The in vivo biodistribution of the infused CD3ε‐Nb EVs was monitored in COLO 205 CDX‐PBMC‐huNSG mice. The results showed that the bioluminescent signal of the infused CD3ε‐Nb EVs, which expressed nanoluciferase‐chimeric CD63 protein (CD3ε‐Nb NLuc EVs), was more concentrated in the spleen, while the signal of control NLuc EVs was concentrated in the lungs (Figure S15A–D), corresponding to the increased human CD63‐positive cells in spleen and decreased in lung compared to NLuc EVs, while both were absent of apparent tissue damage especially in liver, lung and spleen (Figure S15E). To further clarify safety concerns regarding immunogenicity and safety risks associated with repeated infusions of viral and synthetic nanocarriers [9, 10, 11], a CD3ε‐Nb mouse‐CD63 chimeric construct was transfected into mouse fibroblast NIH/3T3 cells to produce mouse cell‐derived CD3ε‐Nb EVs (CD3ε‐Nb mEVs) (Figure S16). Compared with the CD3ε‐Nb liposomes, CD3ε‐Nb LNPs, and CD3ε‐Nb LVs, repeated infusions of the CD3ε‐Nb mEVs triggered minimal production of inflammatory cytokines, including C1q, IFN‐α, IL‐6, IFN‐γ, IL‐1β, and liver damage marker alanine transaminase (ALT), in a C57BL/6 immunocompetent mouse model (Figure 6A,B). These were consistent with the observations in H1975 CDX‐huNSG mice infused with HEK‐293T‐derived CD3ε‐Nb EVs (Figure S17A,B).

Notably, consecutive injections of CD3ε‐Nb EVs did not induce adverse effects in dose escalation experiments conducted in the immunocompetent mouse model (Figure S17C,D). Moreover, unlike liposome, LNP, and LV, consecutive injections of Nb‐CAR.TCE@CD3ε‐Nb mEVs did not induce substantial cytokine secretion in immunocompetent mice (Figure 6B). Furthermore, repeated injection with CD3ε‐Nb mEVs triggered lower antibody responses against EV components compared to liposome, LNP, and LV approaches (Figure 6C), which corresponded to the retention of transfection capacity into CD3‐positive cells (Figure 6D). Notably, prior exposure to CD3ε‐Nb liposomes or CD3ε‐Nb LNPs did not affect the subsequent transfection of Nb‐CAR.TCE transgene mediated by CD3ε‐Nb EVs, and showed an extended survival rate comparable to consecutive infusion with CD3ε‐Nb mEVs in HLA‐G‐overexpressing GL261‐luc (HLA‐Gov GL261‐luc) CDX C57BL/6 mouse model (Figure 6E‐J). Taken together, we propose that Nb‐engineered EVs are promising platforms for transgene delivery, which may retain transfection efficiency with lower inflammatory and antibody responses, and safety issues compared to lipid‐ and lentiviral‐based carriers.

## 4 Discussion

The in vivo generation of CAR‐T cells holds the potential to mitigate concerns regarding alloimmune responses and the risk of GvHD associated with conventional CAR‐T applications [9, 10, 11]. Recently, EVs have been considered a promising drug delivery platform due to their immunologically inert properties, which improve biocompatibility and bioavailability compared to viral and synthetic nanoparticles [35, 36, 37, 38, 39]. In this study, we engineered CD3ε Nb‐expressing EVs derived from the HEK‐293T cell line through genetic manipulation. Following encapsulation with the Nb‐CAR.TCE transgenes, these CD3ε Nb‐expressing EVs, which possess CD3ε targeting properties, were utilized to treat solid tumors by promoting in vivo Nb‐CAR.TCE‐T cell production. The findings in this study indicate that CD3ε‐Nb EVs could selectively transfect the Nb‐CAR.TCE transgene into CD3^+^ cells both in vitro and in vivo, demonstrating enhanced tumor‐infiltration and persistence, which may contribute to superior antitumor activity and sustained antitumor immunity against solid tumors. The EV‐mediated in vivo CAR‐T strategy exerted anti‐tumor activity comparable to that of conventional ex vivo manufactured CAR‐T, lipid carrier‐mediated, and LV‐based in vivo CAR‐T approaches. Notably, the utilization of CD3ε‐Nb EVs resulted in fewer inflammatory effects, reduced hepatotoxicity, and minimal immunogenic responses compared to lipid carrier and LV‐based in vivo CAR‐T therapy, enhancing its safety and potential for further clinical applications.

Repeated administrations of EVs have been found to elicit antibody responses, coinciding with reduced EV circulation time, which may hinder successful EV‐mediated cargo delivery into tissues [40, 41]. However, unlike other lipid‐based carriers, the CD3ε‐Nb EVs retained their transfection capability to deliver the Nb.CAR.BiTE transgene into CD3^+^ cells after repeated infusions in an allogeneic immunocompetent mouse model (Figure 6C,G). Most importantly, repeated administration of CD3ε‐Nb EVs did not elicit significant cytokine release (Figure 6B; Figure S21B), with lower antibody response to CD3ε‐Nb EV components, suggesting lower inflammatory and antigenic impacts than LNPs, liposomes, and LVs [35, 36, 42]. Moreover, the biodistribution of CD3ε‐Nb EVs showed a significant concentration in the spleen rather than the liver (Figure S19A–D). After repeated infusion, the serum ALT did not increase (Figure 6B), as well as the absence of obvious tissue damage signs (Figure S19E), implying low liver toxicity of CD3ε‐Nb EVs. In addition, prior exposure to liposomes and LNPs showed minimal influence on the transduction of Nb‐CAR.TCE transgene and anti‐tumor activity mediated by the subsequent infusion of Nb‐CAR.TCE@CD3ε‐Nb EVs, suggesting the feasibility of EV‐mediated CAR transgene delivery. Taken together, these observations support that Nb‐CAR.TCE@CD3ε‐Nb EVs offer therapeutic benefits with lower safety concerns, even prior exposure to lipid carrier‐based therapeutics. However, potential issues such as the induction of antibody responses, especially neutralizing antibodies (NAbs), leading to reduced EV cargo delivery, and adverse events such as cytokine storms, as well as the clinically relevant differences between CD3ε‐Nb EVs and viral/LNP carriers, must be closely monitored and carefully managed in clinical practice.

The lipid and protein compositions of EVs reflect their carrier and delivery capabilities [43, 44]. In this study, multiple steps ensured homogeneous EV products and functionality. However, engineering and purification processes for highly purified EV formulations may impair EV properties [45]. We have identified the lipid compositions, membrane proteins, and protein corona in EVs, finding that they remain highly consistent (>99%) regardless of genetic engineering and Nb‐CAR.TCE transgene loading (data not shown). Here, we emphasized a precise transgene‐delivery platform of EVs through engineering with CD3ɛ‐targeting Nb for in vivo CAR‐T therapy. We considered that the manufacturing process has little impact on the vehicle functionality of EVs. Nonetheless, we value these minor lipid and protein composition changes and continue exploring their impacts on EV properties.

Loading EVs with nucleic acid via electroporation may result in siRNA and miRNA aggregates [46, 47]. However, in this study, the Nb‐CAR.TCE DNA transgene (approximately 4700 bp) was preserved after EV encapsulation via electroporation, with no increase in the signal intensity of the higher bp peak (Figure 1G). Therefore, the likelihood of mistaking Nb‐CAR.TCE DNA transgene aggregates for EVs is low, consistent with previous findings [5, 47].

Nb‐CAR.TCE@CD3ε Nb‐EVs treatment following intact PMBCs humanization showed long‐term antitumor surveillance and formation of memory CAR‐T cells responsive to antigen rechallenge, sustaining antitumor immunity (Figure 4). Since HLA‐G is an inhibitory ICP and a TAA as well [48], the expansion of residual HLA‐G‐targeting CAR‐T cells might be due to direct recognition of the rechallenged antigen rather than by antigen trans‐presentation mechanisms. Moreover, compared to Nb‐CAR.TCE@EVs, Nb‐CAR.TCE@CD3ε Nb‐EVs significantly increased memory Nb‐CAR‐T frequencies in splenocytes and TILs (Figure 4 and Figure 5). The modified 4‐1BB‐based CAR construct incorporating an IFNAR1‐derived Tyk‐binding motif and a secretable Nb‐BiTE targeting PD‐L1 promoted TCM/TSCM (or TRM) phenotypes [30, 31, 32, 33, 34], which was associated with a metabolic shift toward enhanced mitochondrial respiration [30], resulting in greater amounts of tumor‐infiltrated Nb‐CAR^+^ T cells with memory phenotype and effector properties. Furthermore, CD3ε‐targeting EVs increased the frequency of transgene integration into the genome, which may further extend the persistence of Nb‐CAR.TCE‐T cells (Figure 3C). In addition, CD3ε‐targeting delivery of Nb‐CAR.TCE transgene also enhanced tumor‐infiltration capacity (Figure 3D,E), which was associated with a reduced occurrence of exhaustion phenotype in Nb‐CAR‐expressing TILs (Figure 3F), modulated immunosuppressive TME, and promoted cytokine polyfunctionality in response to antigen stimulation (Figure 3G). Therefore, we speculated that these elements may synergize and contribute to effective and prolonged immune surveillance against HLA‐G‐expressing tumor cells. However, the integration site of Nb‐CAR.TCE transgene and the risk of clonality in the target immune cells should be investigated before clinical applications.

The current EV preparation processes may not offer a significant advantage over CAR‐T cell products [5]. However, despite similar manufacturing duration, ready‐to‐use, off‐the‐shelf EV products for in vivo CAR‐T therapy can greatly benefit advanced cancer patients. Moreover, compared with synthetic carriers, lentiviral particles (Figure 6B; Figure S21B) and allogeneic CAR‐T cells, the EV‐driven in vivo generation of CAR‐T cells offers better biocompatibility and lower inflammatory risks, making it a promising cancer treatment option with low safety risks. On the other hand, there are critical issues that need to be overcome before further clinical application. For example, selecting stable clones with high EV yields and using suitable bioreactors and media will be crucial for scaling up EV production. Furthermore, circularizing the Nb‐CAR.TCE transgene and optimizing electroporation conditions may improve loading capacity, transgene stability, and expression efficiency in vivo [49, 50].

In conclusion, we developed CD3ε Nb‐CD63 chimeric protein‐expressing EVs, manufactured from a genetically engineered HEK‐293T stable clone, loaded with linear DNA transgenes through electroporation for in vivo generation of HLA‐G/PD‐L1 dual targeting Nb‐CAR.TCE‐T cells to treat solid tumors. The CD3‐targeted EVs could selectively deliver the CAR transgene into CD3^+^ immune cells and enhance memory T cell formation, resulting in long‐term anti‐tumor activity. The advancement of this CD3‐targeting delivery EV platform for in vivo CAR‐T therapy offers promise in addressing safety concerns and limitations associated with synthetic and viral particle platforms and holds potential for broader clinical applications in solid tumor treatment.

## Author Contributions

S.W.H., S.C.C., and D.Y.C. designed the study. S.W.H., Y.C.L., Y.C., C.M.P., Y.T.C., C.Y.L., P.Y.L., Y.H.H., S.R., W.Y.M., C.Y.C., K.W.K., W.L.C., C.K.C., P.W.H., Z.L.L., and S.T.W. conducted the experiments. S.W.H., C.M.P., Y.C.L., M.C.L., C.C.W., and S.C.C. acquired data. S.W.H., Y.C.L., C.M.P., S.T.W., M.Y.S., and Y.W.C. analyzed data. S.W.H., M.C.C., S.C.C., and D.Y.C. drafted and edited the manuscript.

## Funding

This study was supported by the Ministry of Science and Technology (SWH: MOST 112‐2314‐B‐039 ‐052 ‐, DYC: MOST 112‐2321‐B‐039 ‐008 ‐, SCC: MOST 112‐2314‐B‐039 ‐053 ‐, CMP: NSTC 112‐2320‐B‐039‐013‐), China Medical University Hospital (EXO‐113‐013 and DMR‐CARGDT‐01 to SWH and DYC, EXO‐112‐011 and EXO‐113‐014 to CMP), and the Ministry of Education (MOHW108‐TDU‐B‐212‐124024 to DYC and SCC, Higher Education Sprout Project).

## Ethics Statement

The acquisition of human peripheral blood mononuclear cell samples from donors and tissue sample studies was approved by the Research Ethics Committee of China Medical University & Hospital, Taichung, Taiwan (CMUH112‐REC3‐186). All conducted animal studies were approved by the local ethics committee of the Institutional Animal Care and Use Committee of China Medical University Hospital (CMUIACUC‐2023‐092).

## Consent

All PBMC donors confirmed the informed consent.

## Conflicts of Interest

Dr. Yu‐Chuan Lin is employed by Shine‐On BioMedical Co. Ltd., Taichung, Taiwan. Dr. Ming‐Chao Liu is employed by Ever Supreme Bio Technology, Taichung, Taiwan. Dr. Sin‐Ting Wang is employed by Shine Out Bio Technology Co. Ltd., Taichung, Taiwan. None of the authors receives any grants from Shine‐On BioMedical Co. Ltd., Ever Supreme Bio Technology, or Ever Health Bio Medical International Co. Ltd.

## Supporting information




**Supporting File**: advs74124‐sup‐0001‐SuppMat.docx.

## Data Availability

All data associated with this study are available in the main text or supplementary materials.
