# Supplementary material for: CD3ɛ Nanobody‐Engineered Extracellular Vesicles Driving In Vivo Generation of TCE‐secreting CAR‐Ts for Solid Tumor Therapy With Memory Response and Minimal Immunogenicity
Source: Adv Sci (Weinh). 2026 Feb 3;13(19):e19440. doi: 10.1002/advs.202519440 (PMC13045241; doi:10.1002/advs.202519440)
Supplement: Supplementary file 1 — Supporting File: advs74124‐sup‐0001‐SuppMat.docx. [file ADVS-13-e19440-s001.docx]

**Supplementary Figures**


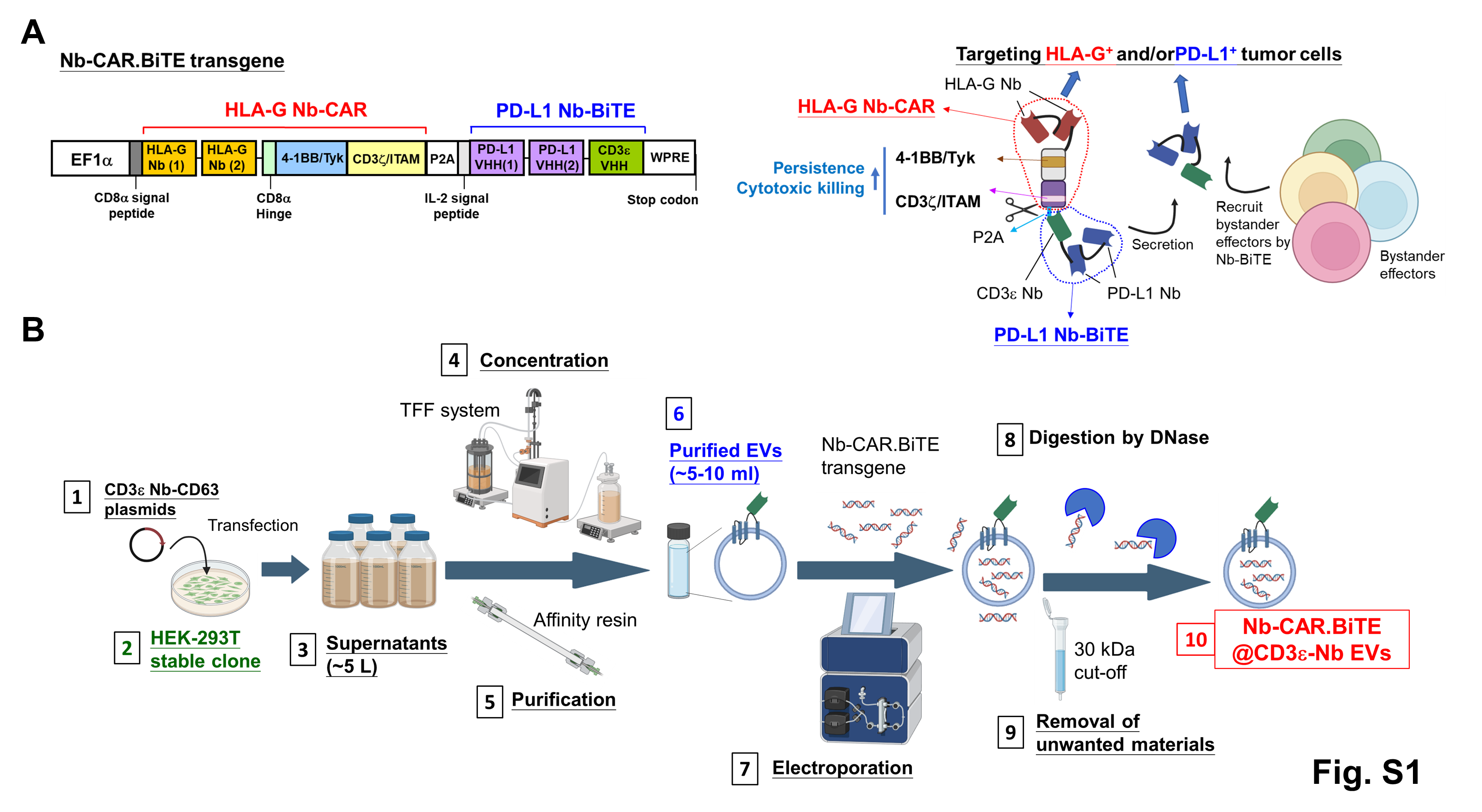


**Fig. S1** Representative diagram for the generation of Nb-CAR.BiTE@CD3ε-Nb EVs. **A** The bi-epitopic Nb-CAR comprises two tandem extracellular HLA-G-targeting Nbs fused with a CD8α hinge, followed by a modified 4-1BB transmembrane and a cytosolic domain inserted with a Tyk2-binding domain derived from IFNAR1(4-1BB/Tyk), and a modified CD3ξ intracellular domain integrated with an ITAM derived from DAP-12 (CD3ξ/ITAM) linked to a self-cleavage peptide P2A to separate the secretable bivalent Nb-BiTE. The Nb-BiTE consists of two anti-PD-L1 Nbs linked to a CD3ɛ Nb. This construct, driven by an EF-1α promoter, has a post-transcriptional regulatory element (WPRE) at the 3’-terminal (Left panel). The secreted Nb-BiTE may recruit and activate bystander effector cells against PD-L1-expressing tumor cells, collaborating with HLA-G Nb-CAR-T cells to attack HLA-G^+^ and or PD-L1^+^ tumor cells. The inserted IFNAR1-derived Tyk2-binding domain and DAP-12-derived ITAM are designed to increase T-cell persistence and enhance cytotoxic killing activity, respectively (Right panel). **B** Representative diagram of the manufacturing process for HEK-293 stable cell line-derived CD3ε-Nb EVs and Nb-CAR.BiTE transgene payload through electroporation. Briefly, the plasmid encoding the CD3ε Nb-CD63 chimeric protein was transfected into HEK-293T cells, followed by stable cell line selection. Supernatant from a stable cell line culture (approximately 5 liters) was harvested and subjected to EV isolation via a TFF system to concentrate EVs and deplete cell debris, and then purified to approximately 5-10 mL via Anti-Camelid VHH-Affinity Resin to obtain CD3ε Nb-expressing EVs (CD3ε-Nb EVs). The enriched EVs were then loaded with Nb-CAR.BiTE transgene via electroporation using a Lonza Amaxa 4D Nucleofector instrument. After digestion of the unencapsulated transgene by incubation with DNase, the Nb-CAR.BiTE transgene-encapsulated CD3ε-Nb EVs (Nb-CAR.BiTE@CD3ε-Nb EVs) were purified by filtration through a 30-kDa membrane to remove the unencapsulated transgene, residual electrolytic buffer, and DNase I.


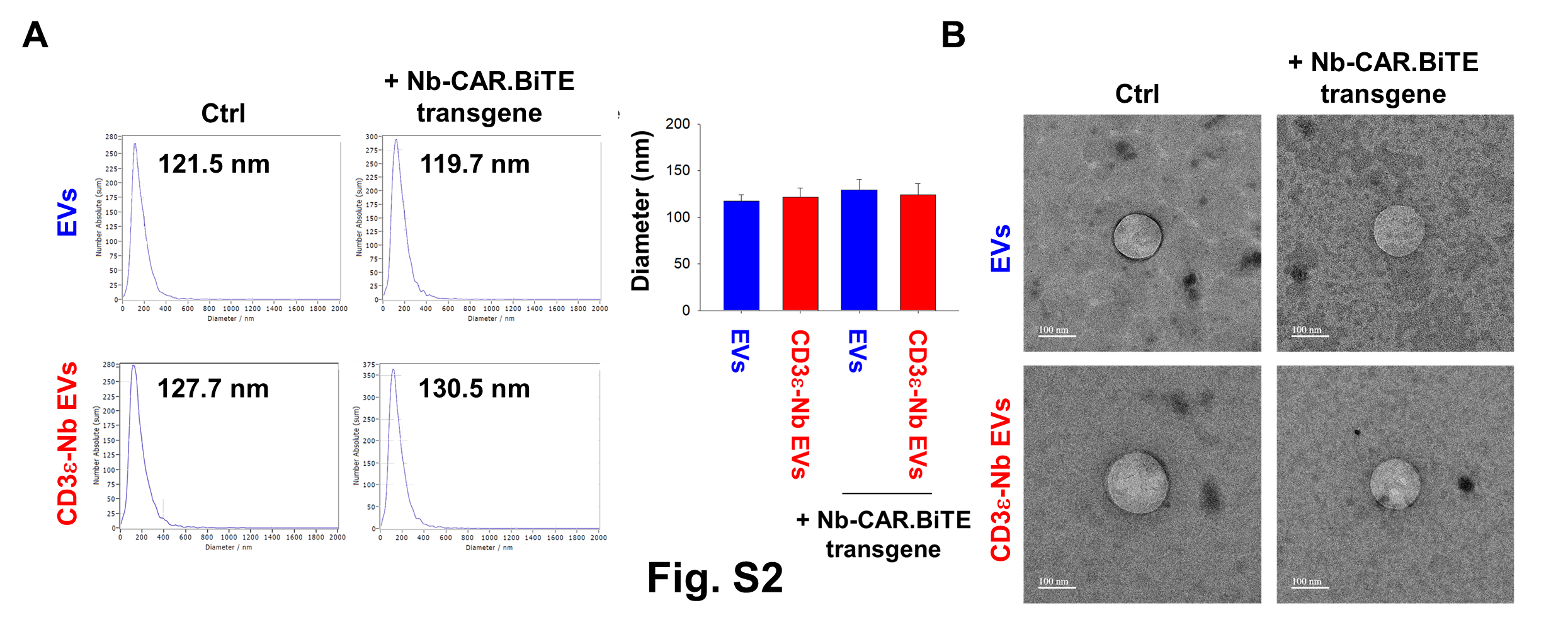


**Fig. S2** Nb-CAR.BiTE transgene-loaded CD3ε-Nb EVs preserve exosome ultrastructure and morphology. The particles and ultrastructure of unmodified and CD3ε Nb-expressing EVs loaded with or without Nb-CAR.BiTE transgenes were assessed by **(A)** NTA analysis and **(B)** TEM at 100,000X. Results are representative of three independent experiments. Data are mean ± SD, ****p* < 0.001.


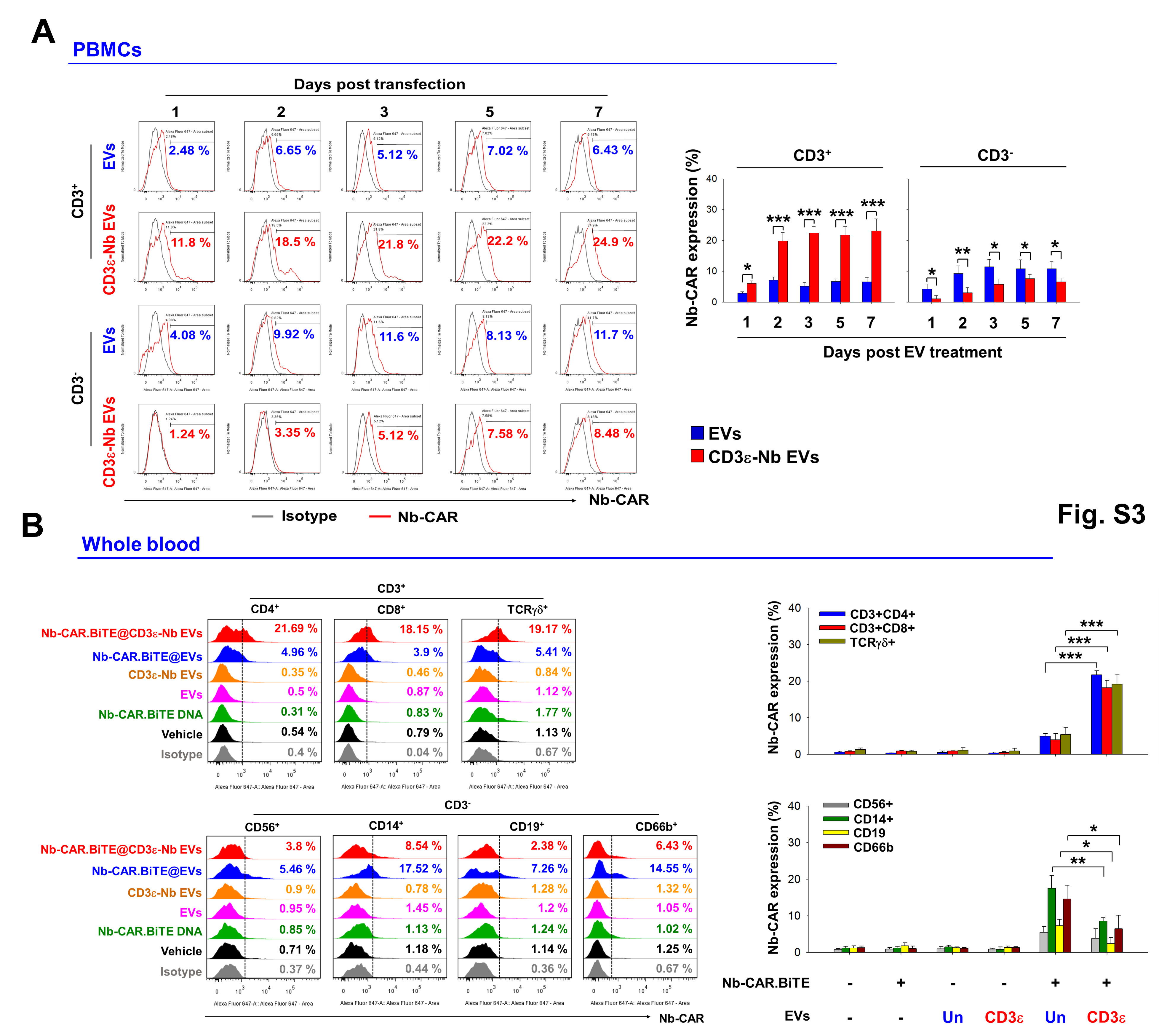


**Fig. S3.** CD3ε-Nb EVs selectively transfect Nb-CAR.BiTE transgene into CD3^+^ cells *in vitro*. **A, B** CD3ε-Nb EVs selectively deliver Nb-CAR.BiTE transgene into CD3^+^ cells *in vitro*. Unmodified EVs or CD3ε-Nb EVs (3 × 10^8^ particles) were electroporated with or without Nb-CAR.BiTE transgene (2 μg). These transgene-loaded EVs were added to 1 × 10^6^ PBMCs and incubated for **A** 1, 2, 3, 5, and 7 days. Afterward, the expression levels of Nb-CAR on CD3^+^ and CD3^-^ cells were determined by flow cytometry. **B** Human whole blood (2 ml) was treated with unmodified or CD3ε-Nb EVs (3 × 10^8^ particles) loaded with or without Nb-CAR.BiTE transgene, for 3 days. The frequency of Nb-CAR^+^ proportions in CD3^+^ populations (including CD4^+^, CD8^+,^ and TCRγδ^+^) and CD3^-^ populations (including CD56^+^, CD14^+^, CD19^+,^ and CD66b^+^) was analyzed by flow cytometry analysis. The results of the *in vitro* experiments are representative of four independent experiments. Data are mean ± SD, **p* < 0.05, ***p* < 0.01, ***p* < 0.001; Student’s t-test and paired Student’s t-test.


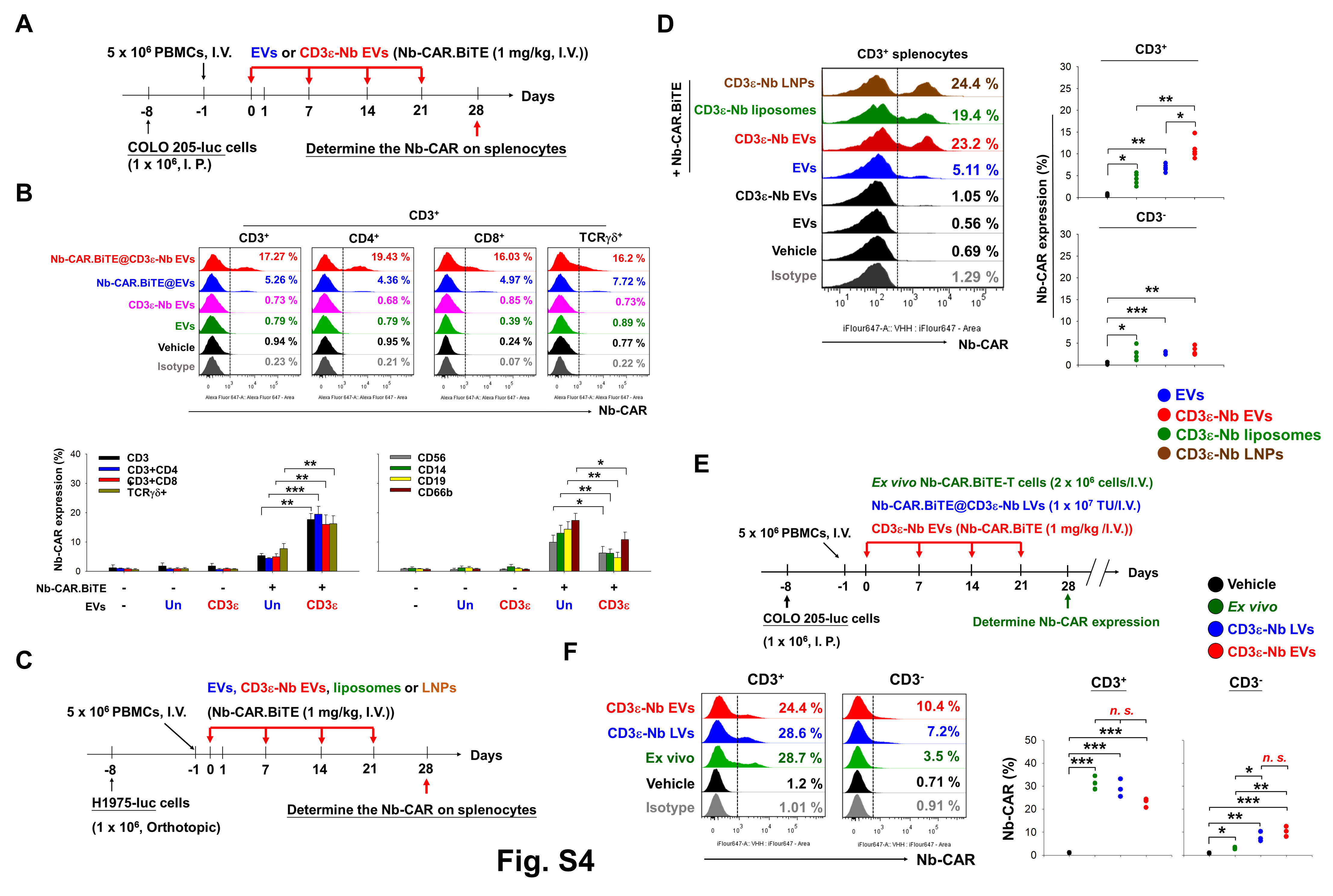


**Fig. S4** CD3ε-Nb EVs selectively transfect Nb-CAR.BiTE transgene into CD3^+^ cells *in vivo*. **A, B** CD3ε-Nb EVs selectively deliver Nb-CAR.BiTE transgene into CD3^+^ cells *in vivo*. **(A)** Representative protocol for determining the efficiency of *in vivo* generation of Nb-CAR.BiTE-T cells driven by CD3ε-Nb EVs. On Day 7 after intraparietal implantation of COLO 205-luc cells (1 × 10^6^ cells), mice (*n* = 5) were injected via the tail vein with human PBMCs (huPBMCs, 5 × 10^6^). The next day, mice were infused with unmodified or CD3ε-Nb EVs encapsulated with or without Nb-CAR.BiTE transgene (1 mg/kg/mouse) once a week for four weeks. **B** The expression levels of Nb-CAR on CD3^+^, CD4^+^, CD8^+^, TCRγδ^+^, CD56^+^, CD14^+^, CD19^+^, and CD66b^+^ cells were determined by flow cytometry. **C, D** CD3ε-Nb EVs exerted similar CD3-targeted transfection efficiency compared to CD3ε-Nb liposomes and CD3ε-Nb LNPs *in vivo*. (**C)** Schematic representation of the protocol for evaluating *in vivo* Nb-CAR.BiTE transgene delivery by CD3ε-Nb EVs, liposomes, and LNPs. After 7 days of orthotopic implantation with luciferase-expressing H1975 cells (1 × 10^6^ cells), the mice (*n* = 5) were tail vein injected with huPBMCs (5 × 10^6^). The next day, mice were infused with or without unmodified or CD3ε-Nb EVs, CD3ε Nb-conjugated liposomes, or LNPs, encapsulated with 1 mg/kg of Nb-CAR.BiTE or Nb-BiTE transgene, once a week for four weeks **(D)**. **E, F** CD3ε-Nb EVs exerted a similar capacity to generate Nb-CAR.BiTE-T cells *in vivo* compared to the LV-based strategy. **E** Schematic protocol for measuring the frequencies of Nb-CAR.BiTE-T cell after treatment with Nb-CAR.BiTE@CD3ε-Nb LVs, Nb-CAR.BiTE@CD3ε-Nb EVs, and the *ex vivo* manufactured Nb-CAR.BiTE-T cells. Seven days post COLO 205-luc cells (1 × 10^6^ cells) implantation, mice (*n* = 3) were infused with huPBMCs (5 × 10^6^). The next day, mice were injected with CD3ε-Nb LVs or CD3ε-Nb EVs encapsulated with or without Nb-CAR.BiTE transgene (1 mg/kg/mouse), or Nb-CAR.BiTE-T cells (2 × 10^6^) once a week for four weeks (**F**). One week after the last infusion, mouse splenocytes were collected and assessed for Nb-CAR expression level on CD3^+^ and CD3^-^ populations by flow cytometry. Data are mean ± SD, **p* < 0.05, ***p* < 0.01, ***p* < 0.001; Student’s t-test and paired Student’s t-test.


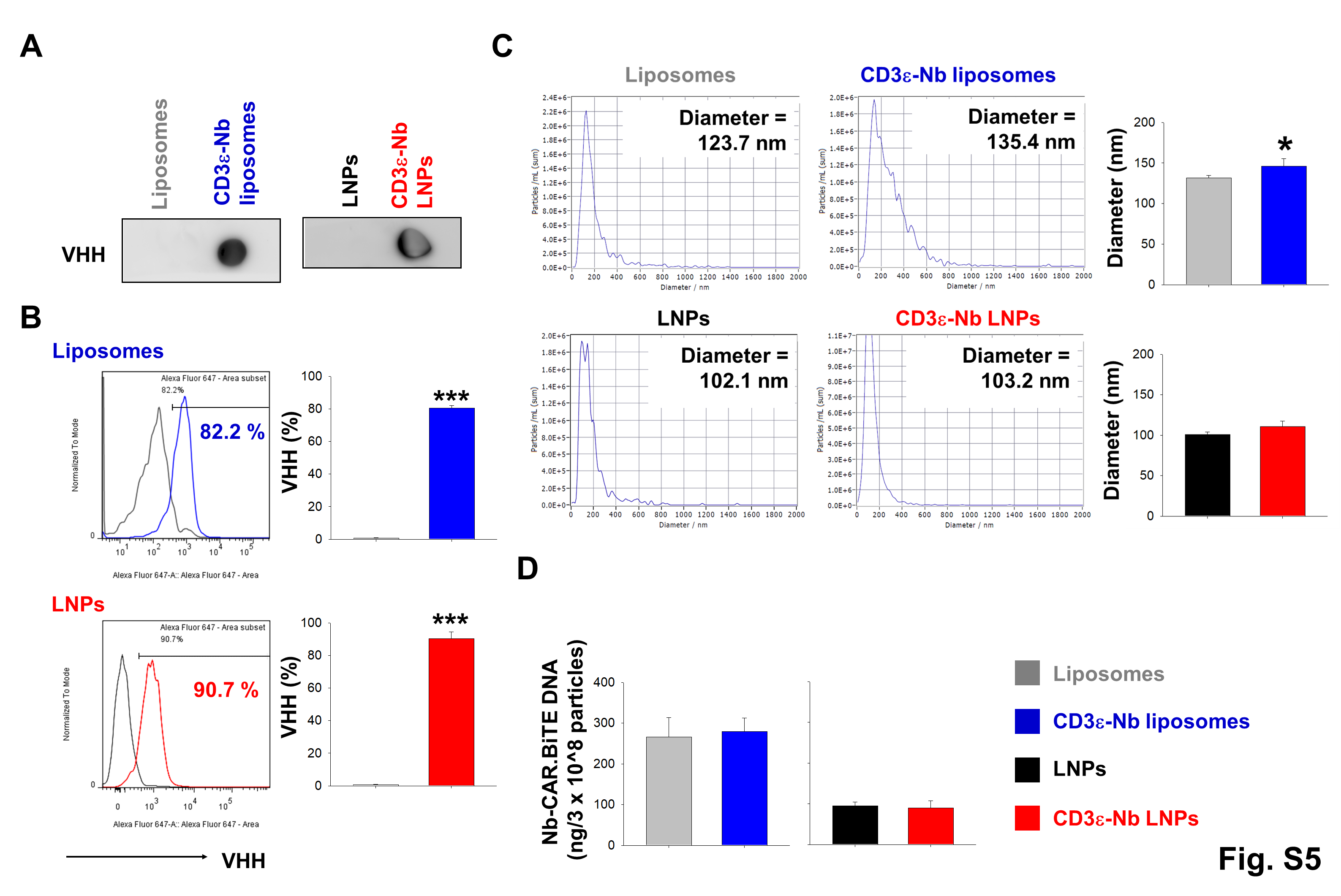


**Fig. S5** The characterization of Nb-CAR.BiTE transgene-encapsulated CD3ε-Nb liposomes and CD3ε-Nb LNPs. The Nb-CAR.BiTE transgene-encapsulated liposomes and LNPs were formulated by NanoAssemblr™ Spark™ nanoparticle formulation system using lipofectamine 3000, subsequently conjugated with recombinant CD3ε Nb through cupper-free azide-alkyne cycloaddition (AAC) click chemistry reaction. **A, B** The CD3ε Nb levels of the CD3ε Nb-conjugated liposomes and LNPs. The levels of CD3ε-Nb moiety on the liposomal (left) and LNPs (right) surface were determined by dot plot **(A)** and nano-flow cytometry (NFC) analysis **(B)** using a VHH-specific antibody. **C** The particle size of CD3ε Nb-conjugated liposomal (left) and LNPs (right) was measured by NTA analysis. **D** The encapsulated Nb-CAR.BiTE transgene in CD3ε Nb-conjugated liposomal (left) and LNPs (right) was quantified by qPCR analysis using specific primers. Results are representative of three independent experiments. Data are mean ± SD, **p* < 0.05; ****p* < 0.001.


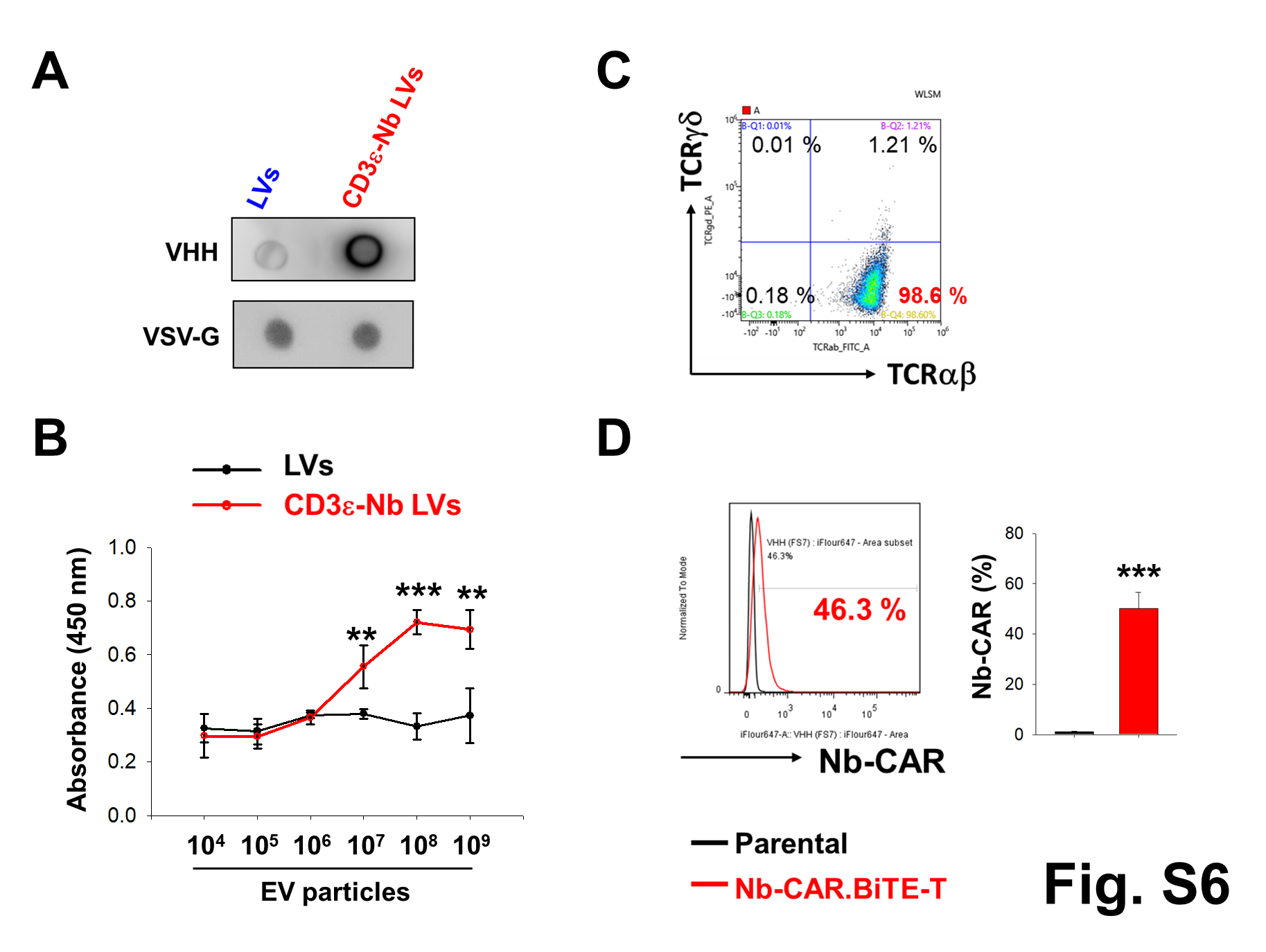


**Fig. S6** The characterization of Nb-CAR.BiTE transgene-carried CD3ε Nb-LVs and conventional *ex vivo* Nb-CAR.BiTE-T cells. The Nb-CAR.BiTE transgene-carried LV particles were harvested from the supernatants of HEK-293T cells after 48 h of co-transfection with pLV plasmids encoding Nb-CAR.BiTE, psPAX2, and CD3ε Nb-VSV-G (or VSV-G). **A** The expression levels of CD3ε-Nb moiety on these LVs were determined by dot plot. **B** The binding affinity of parental LVs and CD3ε-Nb LVs was measured by an ELISA-based assay. **C, D** The generation of *ex vivo* Nb-CAR.BiTE-T cells using LVs. αβT cells were isolated and expanded from PBMCs (**C**), and the expression level of Nb-CAR after 72 h infection with LVs was determined by flow cytometry using a VHH-specific antibody. Results are representative of three independent experiments. Data are mean ± SD, **p* < 0.05; ****p* < 0.001.


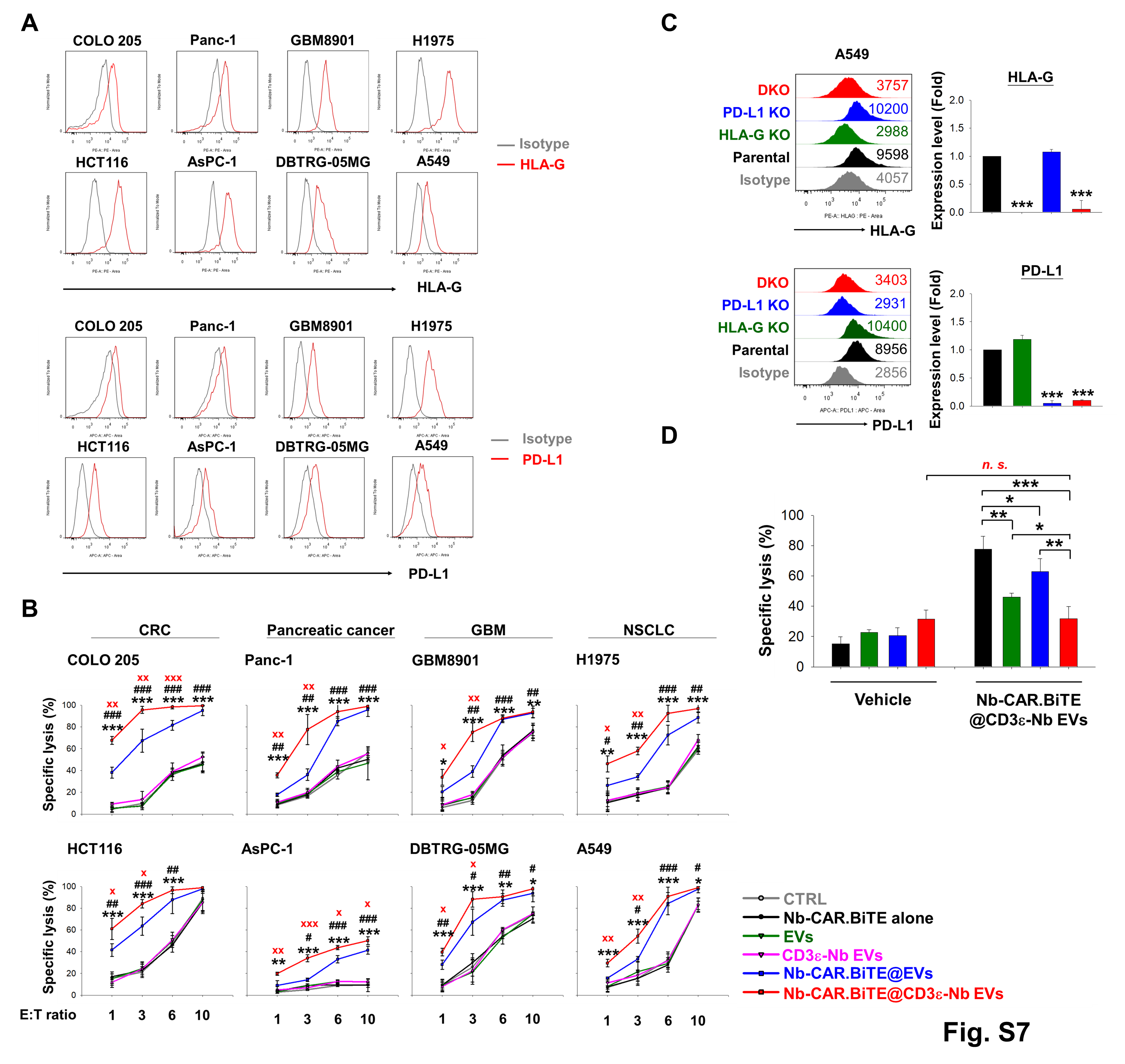


**Fig.** **S7** Nb-CAR.BiTE@CD3ε-Nb EVs enhanced HLA-G and PD-L1-dependent cytotoxicity in PMBCs against various types of solid tumor cells *in vitro*. **A** The expression levels of HLA-G and PD-L1 on tumor cell lines*.* Expression levels of HLA-G and PD-L1 on COLO 205, HCT116, Panc-1, AsPC-1, GBM8901, DBTRG-05MG, and H1975 cells were determined by flow cytometry analysis using specific antibodies. **B** Nb-CAR.BiTE@CD3ε-Nb EVs-transfected PBMCs induced superior cytotoxicity to various solid tumor cell lines. PBMCs were treated with or without unmodified or CD3ε-Nb EVs loaded with or without Nb-CAR.BiTE transgene for 3 days. Then, these PBMCs were cocultured with COLO 205, HCT116, Panc-1, AsPC-1, GBM8901, DBTRG-05MG, H1975, and A549 cells (1 × 10^5^ cells), respectively, at E:T ratios of 1:1, 3:1, 6:1, and 10:1 for 72 h. **C,D** Nb-CAR.BiTE@ CD3ε-Nb EVs enhanced HLA-G and PD-L1-dependent cytotoxic killing of tumor cells by PBMCs. After treatment with or without Nb-CAR.BiTE@CD3ε-Nb EVs, the PBMCs were co-cultured with parental, HLA-G KO, PD-L1 KO, or HLA-G/PD-L1 DKO A549 cells at an E:T ratio of 3:1 for 72 h. After incubation, the PBMCs were co-cultured with H1975 cells at an E: T ratio of 3:1 for 72 h. Subsequently, the induced cytotoxicity in tumor cells was determined using a LIVE/DEAD Cell-Mediated Cytotoxicity Assay by flow cytometry. The results of the *in vitro* experiments are representative of four independent experiments. Data are mean ± SD, *,^#^,^X^*p* < 0.05; **,^##^,^XX^ *p* < 0.01; ***,^###^,^XXX^ *p* < 0.001; asterisk represent significant differences between Nb-CAR.BiTE@CD3ε-Nb EVs and Ctrl; number sign represents significant differences between Nb-CAR.BiTE@unmodified EVs and Ctrl; and capital letter X represent significant differences between Nb-CAR.BiTE@CD3ε-Nb EVs and Nb-CAR.BiTE@unmodified EVs based on paired Student’s t-tests.


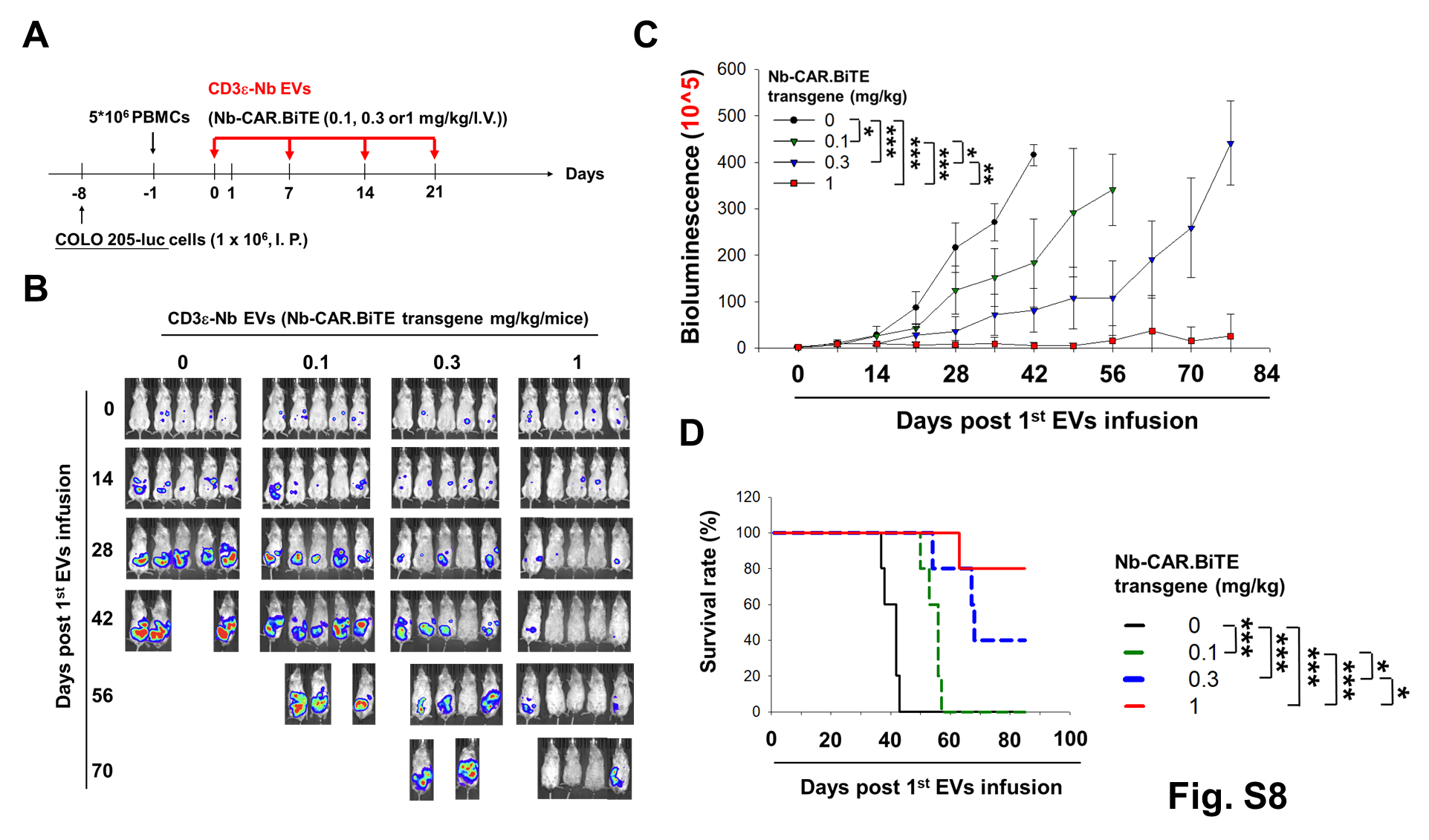


**Fig S8. The dose-dependent anti-tumor activity of Nb-CAR.BiTE@CD3ε-Nb EVs *in vivo*. A.** Schematic representation of the protocol for an *in vivo* dose-dependent anti-tumor efficacy study of Nb-CAR.BiTE@CD3ε-Nb EVs. On day 7 after intraperitoneal implantation of luciferase-expressing COLO 205 cells (1 × 10^6^ cells), mice (*n* = 5) were tail vein injected with hPBMCs (5 × 10^6^). The next day, mice were tail-vein infused with CD3ε-Nb EVs encapsulating 0.1, 0.3, or 1 mg of Nb-CAR.BiTE transgene once a week for four weeks. Tumor growth was monitored weekly using the IVIS system by detecting the bioluminescent signals **(B** and **C)**, and the survival rate of these mice was recorded **(D)**. The *in vivo* tumor growth data were presented as mean ± SEM and analyzed using one-way ANOVA to compare means across multiple groups. Survival rates were analyzed using the Kaplan-Meier method and the log-rank test. **p* < 0.05; ***p* < 0.01; ****p* < 0.001.


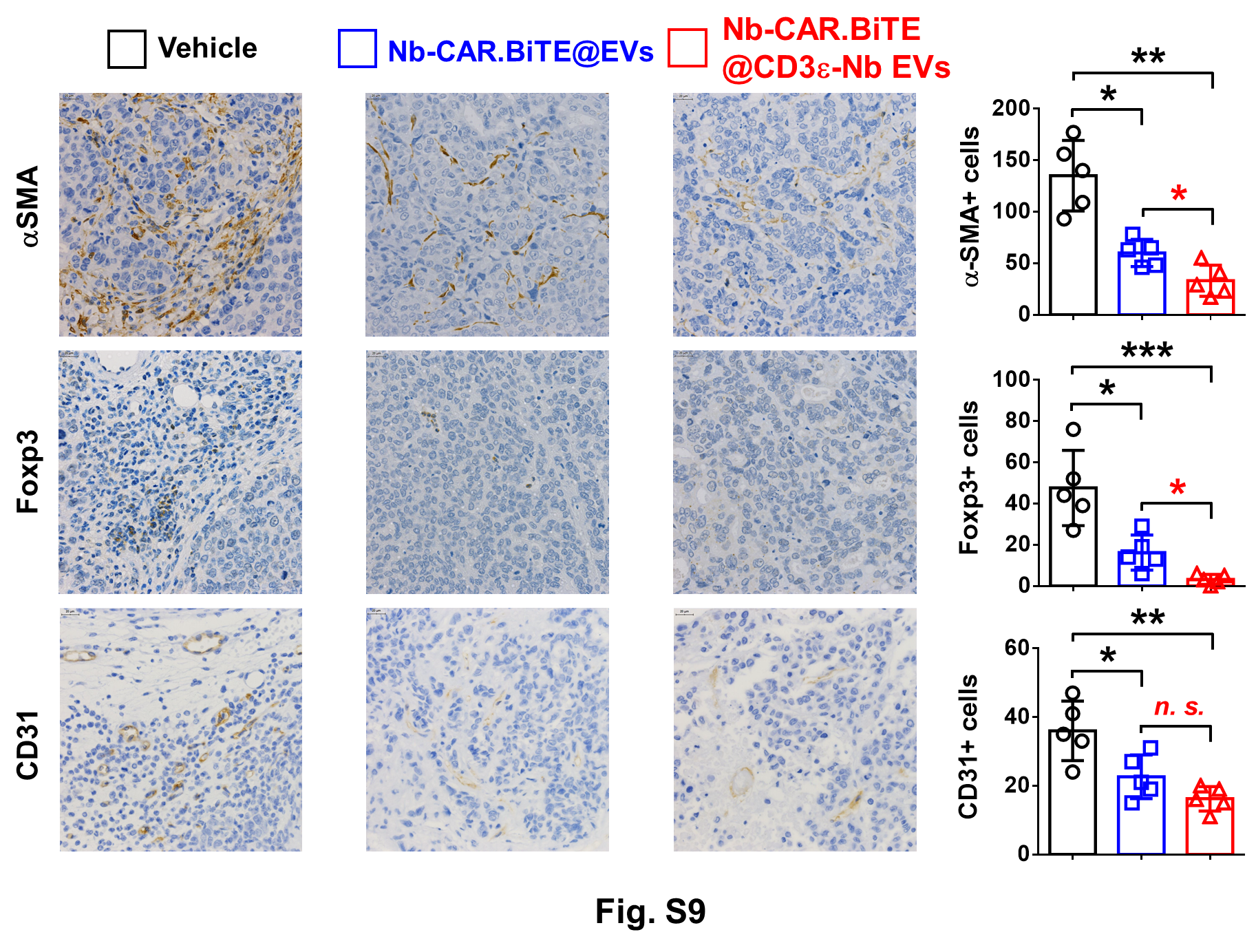


**Fig. S9** Nb-CAR.BiTE@CD3ε-Nb EVs modulate TME. Seven days after intraperitoneal implantation of COLO 205 cells (1 × 10^6^ cells), mice (n = 5) were infused with huPBMCs (5 × 10^6^) through the tail vein. The next day, mice were treated with unmodified or CD3ε-Nb EVs loaded with 1 mg/kg of Nb-CAR.BiTE transgene via tail vein injection weekly for four weeks. On the indicated day, α-SMA, Foxp3, and CD31-expressing cells were detected by IHC staining with specific antibodies. Data are mean ± SD. Statistical significance is set at p-value < 0.05. *p < 0.05, **p < 0.01, ***p < 0.001.


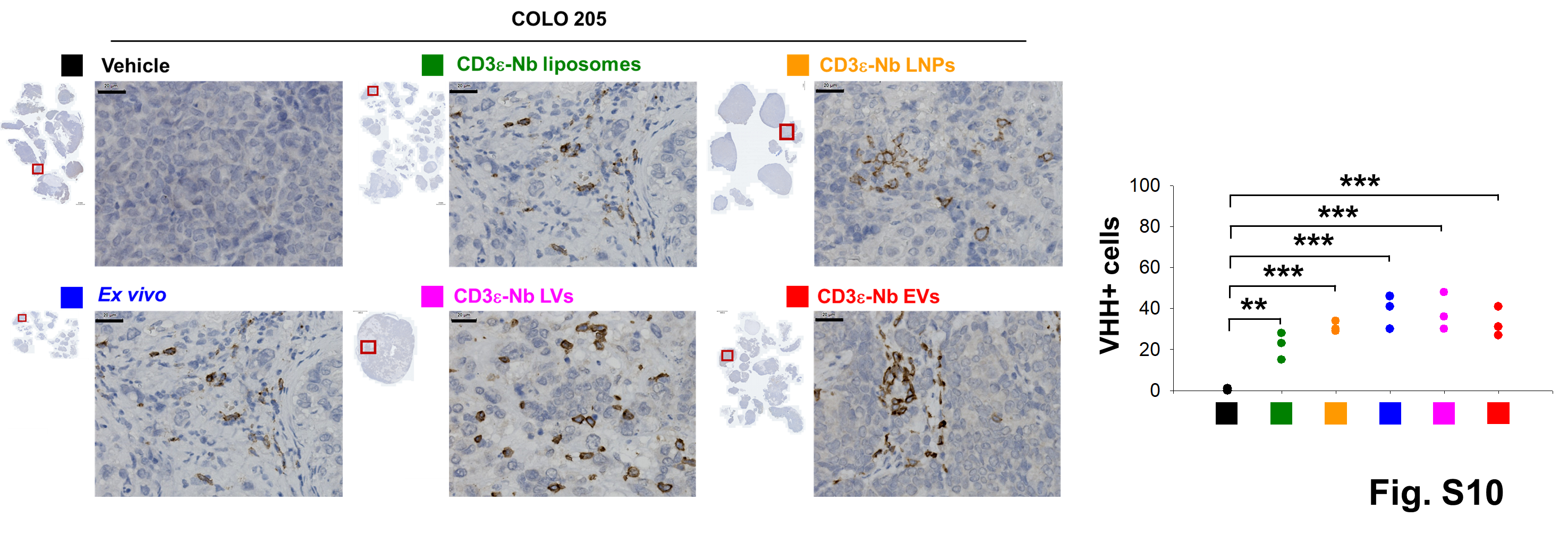


**Fig. S10** Comparable tumor infiltration of Nb-CAR-expressing cells after treatment with conventional *ex vivo* CAR-T cells, and with CD3ε-Nb liposome-, CD3ε-Nb LNP-, CD3ε-Nb LV-, and CD3ε-Nb EV-mediated *in vivo* CAR-T therapies. Seven days after intraperitoneal implantation of COLO 205-luc cells (1 × 10^6^ cells), NSG mice (*n* = 5) were injected with huPBMCs (5 × 10^6^) via the tail vein. The next day, mice were infused with CD3ε-Nb liposomes, CD3ε-Nb LNPs, CD3ε-Nb LVs, or CD3ε-Nb EVs encapsulated with Nb-CAR.BiTE transgene (1 mg/kg), or 2 × 10^6^ *ex vivo* cultivated Nb-CAR.BiTE-T cells weekly for four weeks. On day 28 post the first infusion, all mice were sacrificed, and the presence of Nb-CAR-expressing cells in COLO 205 tumor sections was detected by IHC using a VHH-specific antibody. Data are mean ± SD. Statistical significance was set at *p*-value < 0.05. ***p* < 0.01, ****p* < 0.001.


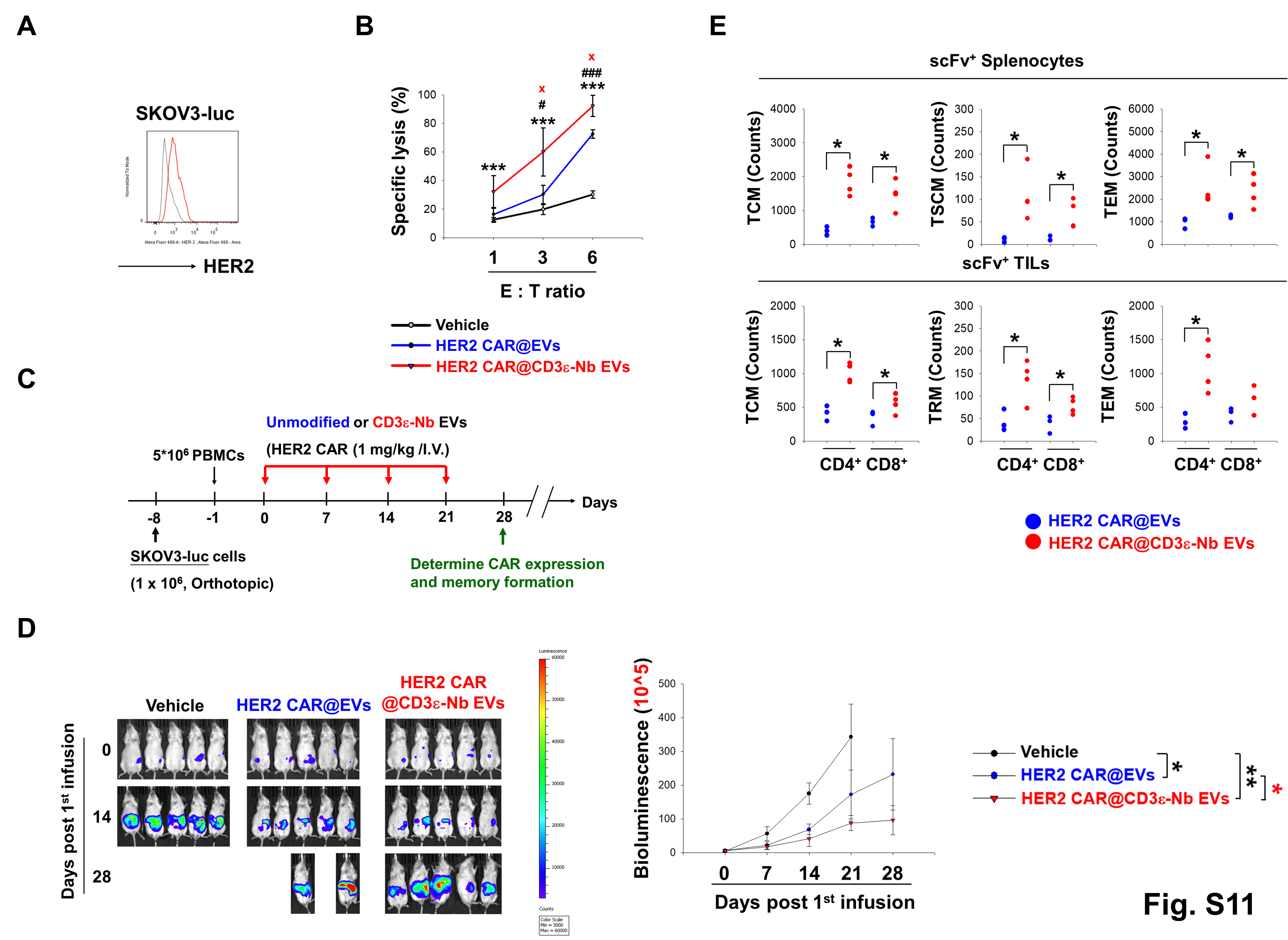


**Fig. S11** The anti-tumor activity of HER2-CAR@CD3ε-Nb EVs. **A** The expression of HER2 on luciferase-expressing SKOV3 cells was measured by flow cytometry. **B** After coculturing with PBMCs, HER2-CAR@EVs, or HER2-CAR@CD3ε-Nb EVs for 72 h, the induced cytotoxicity was measured using the LIVE/DEAD Cell-Mediated Cytotoxicity Assay Kit and analyzed through flow cytometry. **C** Schematic representation of protocol for *in vivo* anti-tumor efficacy study of HER2-CAR@CD3ε-Nb EVs. On day 7 after orthotopic implantation of SKOV3-luc cells (1 × 10^6^ cells), mice (*n* = 5) were infused with huPBMCs (5 × 10^6^) through the tail vein. The next day, mice were injected with unmodified or CD3ε-Nb EVs encapsulated with 1 mg/kg of the HER2-CAR transgene via the tail vein once a week for 4 weeks. Tumor growth rate was monitored weekly by the IVIS system through the bioluminescence channel **(D)**. All mice were sacrificed on day 28 post the first infusion, and their memory status of HER2-CAR-expressing splenocytes and TILs was determined by flow cytometry analysis using specific antibodies against scFv, CD4, CD8, CD27, CD45RA, CD95, and CD103. The *in vivo* tumor growth data were presented as mean ± SEM and analyzed using one-way ANOVA to compare means across multiple groups. Data are mean ± SD. Statistical significance was set at *p*-value < 0.05. **p* < 0.05, ***p* < 0.01, ****p* < 0.001.


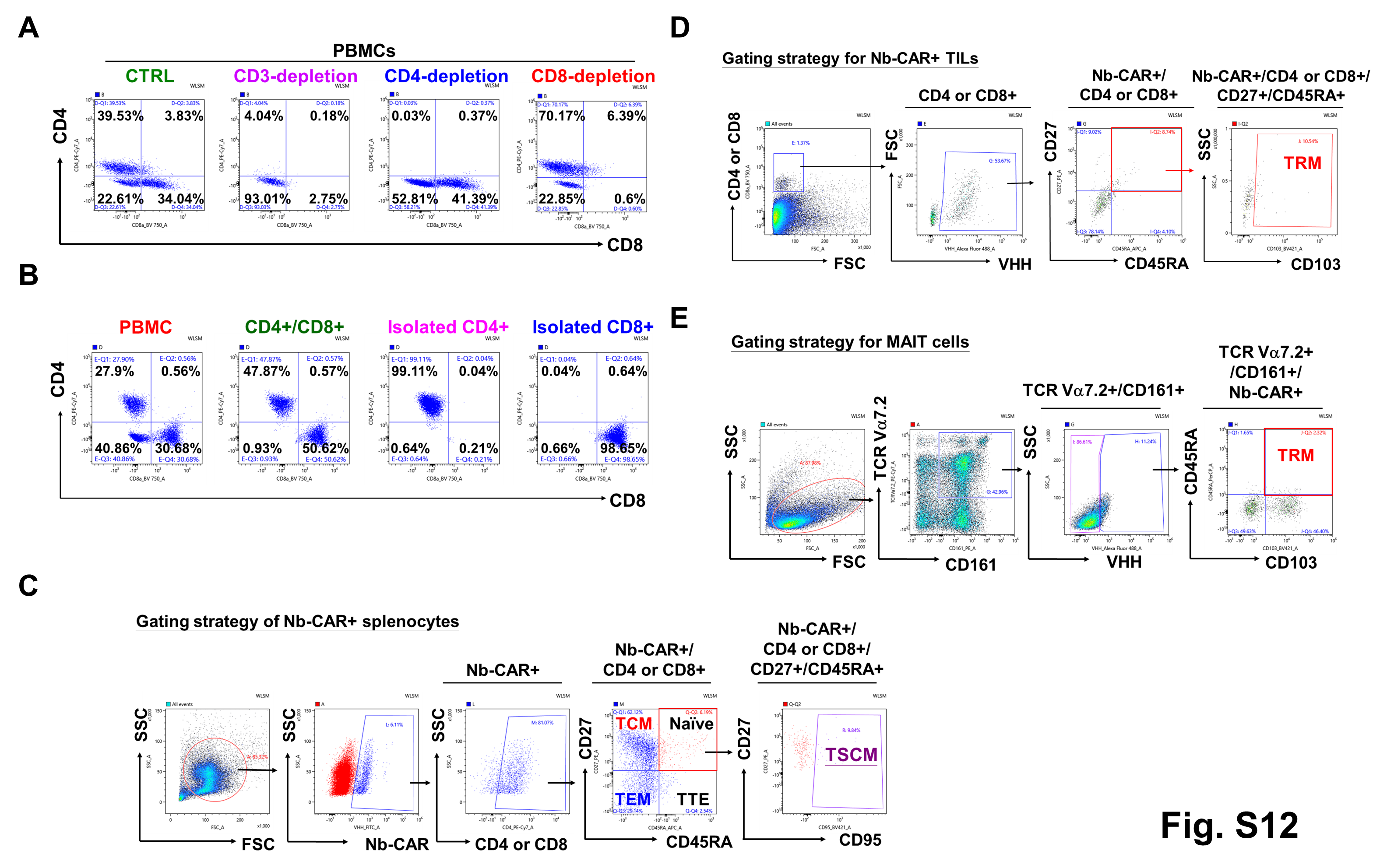


**Fig. S12** Gating strategies for PBMC cell frequencies, and the memory status in splenocytes, TILs, CD4^+^CD8^+^ DP, and MAIT cells. **A, B** The cell frequencies of PBMCs, CD3-depleted, CD4-depleted, and CD8-depleted PBMCs **(A)**; and the isolated CD4^+^ and/or CD8^+^ cells **(B)** were analyzed by flow cytometry using specific antibodies. **C-E** The memory phenotypic markers, including CD4, CD8, CD27, CD45RA, CD95, and CD103, on Nb-CAR-expressing CD4^+^ and/or CD8^+^ splenocytes **(C)**, TILs **(D)**, and CD161^+^TCR Vα2^+^ MAIT cells (**E**) were analyzed by flow cytometry.


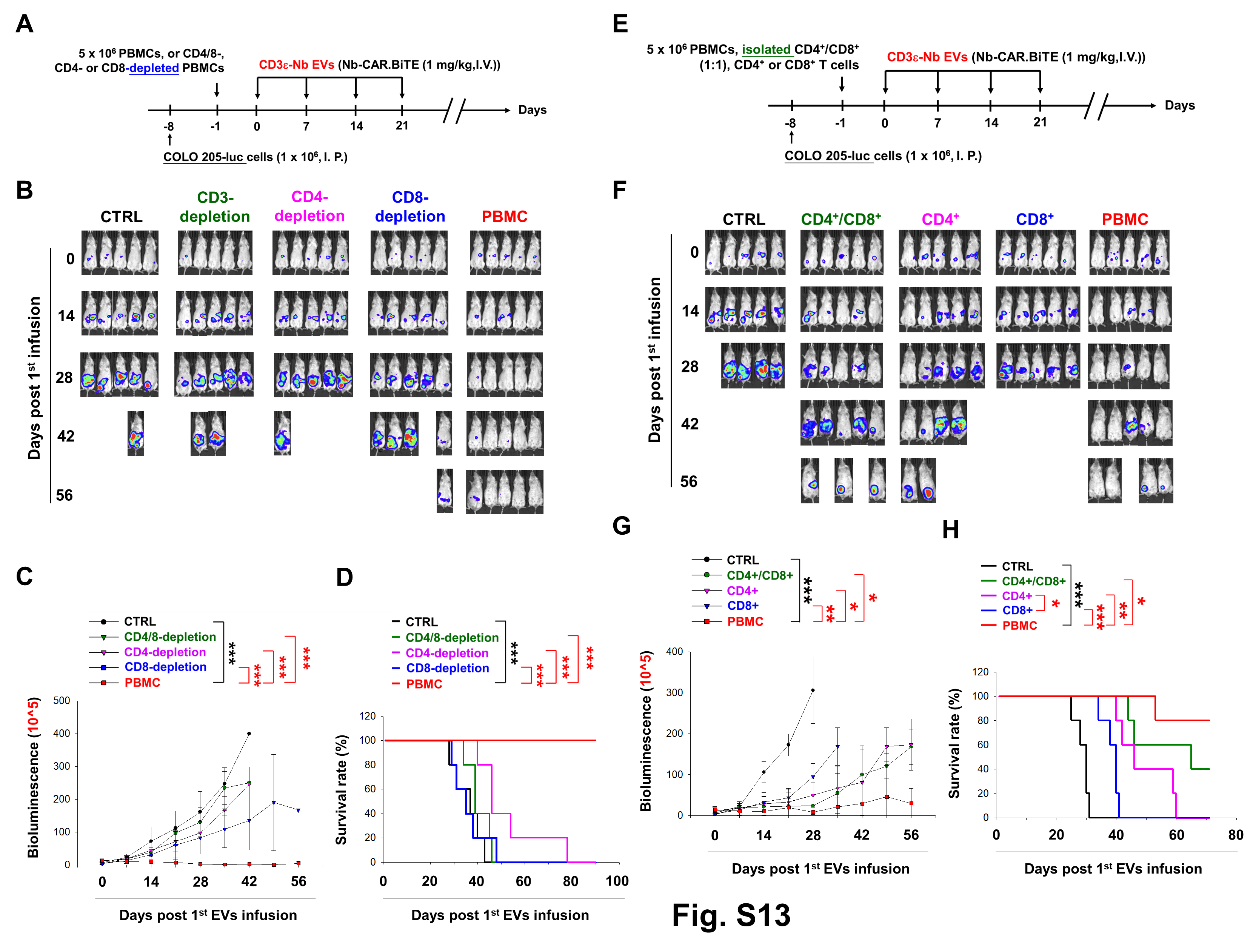


**Fig. S13** CD4^+^ and CD8^+^ T cells were dominant in anti-tumor activity triggered by Nb-CAR.BiTE@CD3ε-Nb EVs *in vivo*. **A** Schematic diagram of the animal model for evaluating the contribution of CD4^+^ and CD8^+^ cells within PBMCs in Nb-CAR.BiTE@CD3ε-Nb EV-induced anti-tumor effect. Seven days after intraperitoneal implantation with 1 × 10^6^ luciferase-expressing COLO 205 cells, mice were infused with 5 × 10^6^ of intact PBMCs, CD4/8- double depleted, CD4-depleted, or CD8-depleted PBMCs through tail vein injection. The next day, mice were treated with CD3ε-Nb EVs encapsulated with Nb-CAR.BiTE transgene (1 mg/kg) once a week for four weeks. The tumor growth rate was monitored by the IVIS system using bioluminescence channels **(B, C)**, and the survival rate was recorded (*n* = 5) **(D)**. **E-H.** The concurrent presence of CD4^+^ and CD8^+^ T cells contributed to Nb-CAR.BiTE@CD3ε-Nb EV-enhanced anti-tumor effect *in vivo*. **E** Schematic protocol for evaluating the contribution of CD4^+^ and CD8^+^ cells in Nb-CAR.BiTE@CD3ε-Nb EV-induced anti-tumor effect. On day 7 after intraperitoneal implantation with 1 × 10^6^ COLO 205-Luc cells, mice were tail vein injected with 5 × 10^6^ PBMCs, CD4^+^, CD8^+,^ or both CD4^+^ and CD8^+^ cells (1:1). On the following day, mice were treated with CD3ε-Nb EVs containing 1 mg/kg encapsulated Nb-CAR.BiTE transgene once a week for four weeks. **F, G** The tumor growth rate was monitored by the IVIS system through the bioluminescence channel, and the survival rate was recorded (*n* = 5) **(H)**. The in vivo tumor growth data are presented as mean ± SEM and analyzed using one-way ANOVA to compare means across multiple groups. The The survival rate was analyzed using the Kaplan–Meier method and log-rank test. Statistical significance was set at *p-value* < 0.05. **p* < 0.05, ***p* < 0.01, ****p* < 0.001.


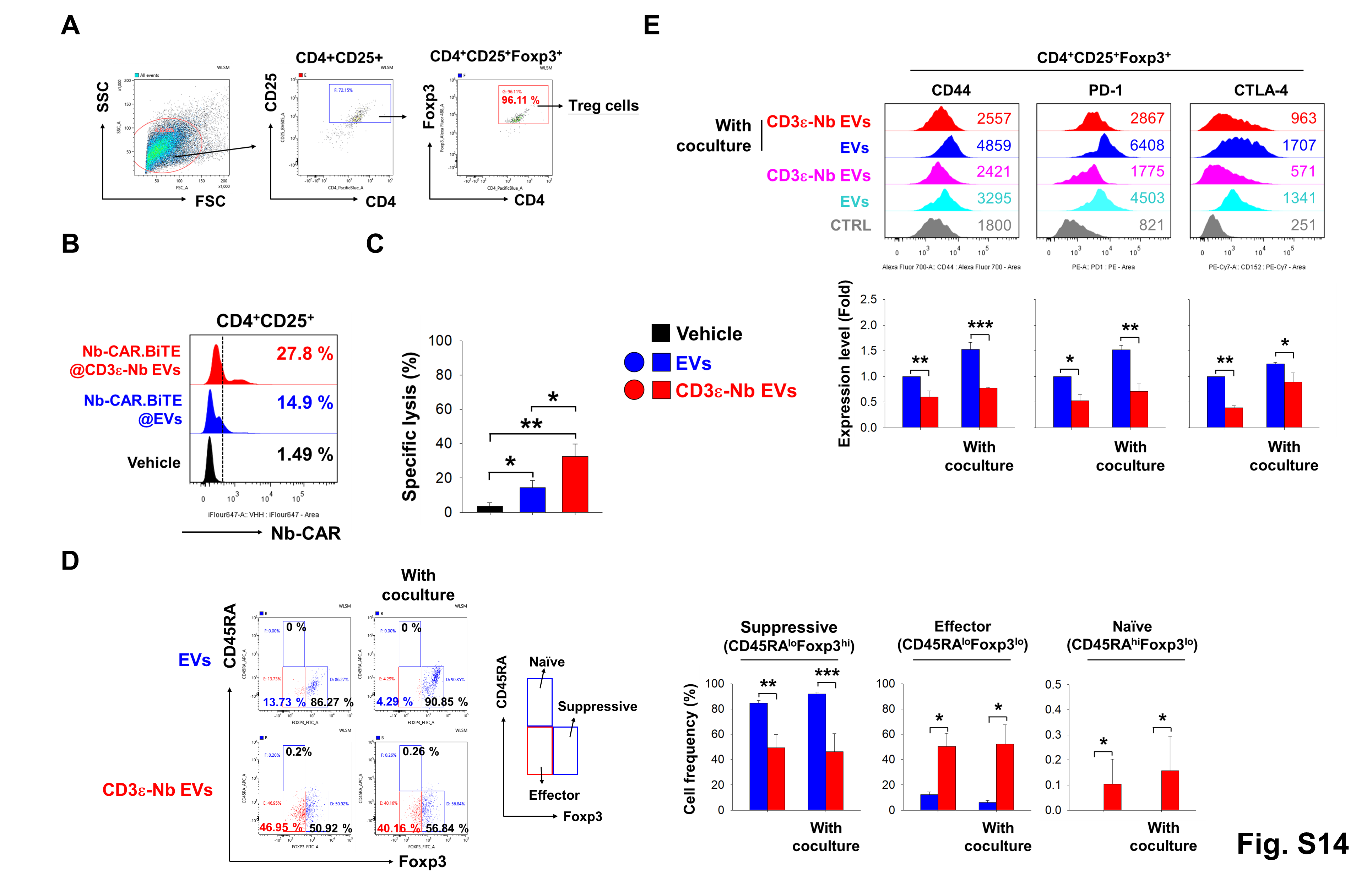


**Fig. S14** Nb-CAR.BiTE@CD3ε-Nb EVs converted Treg cells into effector cells against tumor cells. **A** Treg cell population was enriched from PBMCs using the Cloudz Human Treg Expansion Kit, according to the user instructions. The frequency of Treg cells was determined by flow cytometry using CD4, CD25, and Foxp3 antibodies. After 72 h of transfection with Nb-CAR.BiTE@EVs and Nb-CAR.BiTE@CD3ε-Nb EVs, the expression levels of Nb-CAR on Treg cells were determined (**B**), or subsequently cocultured with or without COLO 205 cells at an E:T ratio of 3:1 for 72 h, the induced cytotoxic killing capacity was determined by LIVE/DEAD Cell-Mediated Cytotoxicity Assay (**C**). Their phenotypic (**D**) and activating (**E**) markers were detected by flow cytometry using specific antibodies against CD4, CD25, Foxp3, CD45RA, CD44, PD-1, and CTLA-4. The results of the *in vitro* experiments are representative of four independent experiments. Data are mean ± SD, **p* < 0.05; ** *p* < 0.01; *** *p* < 0.001.


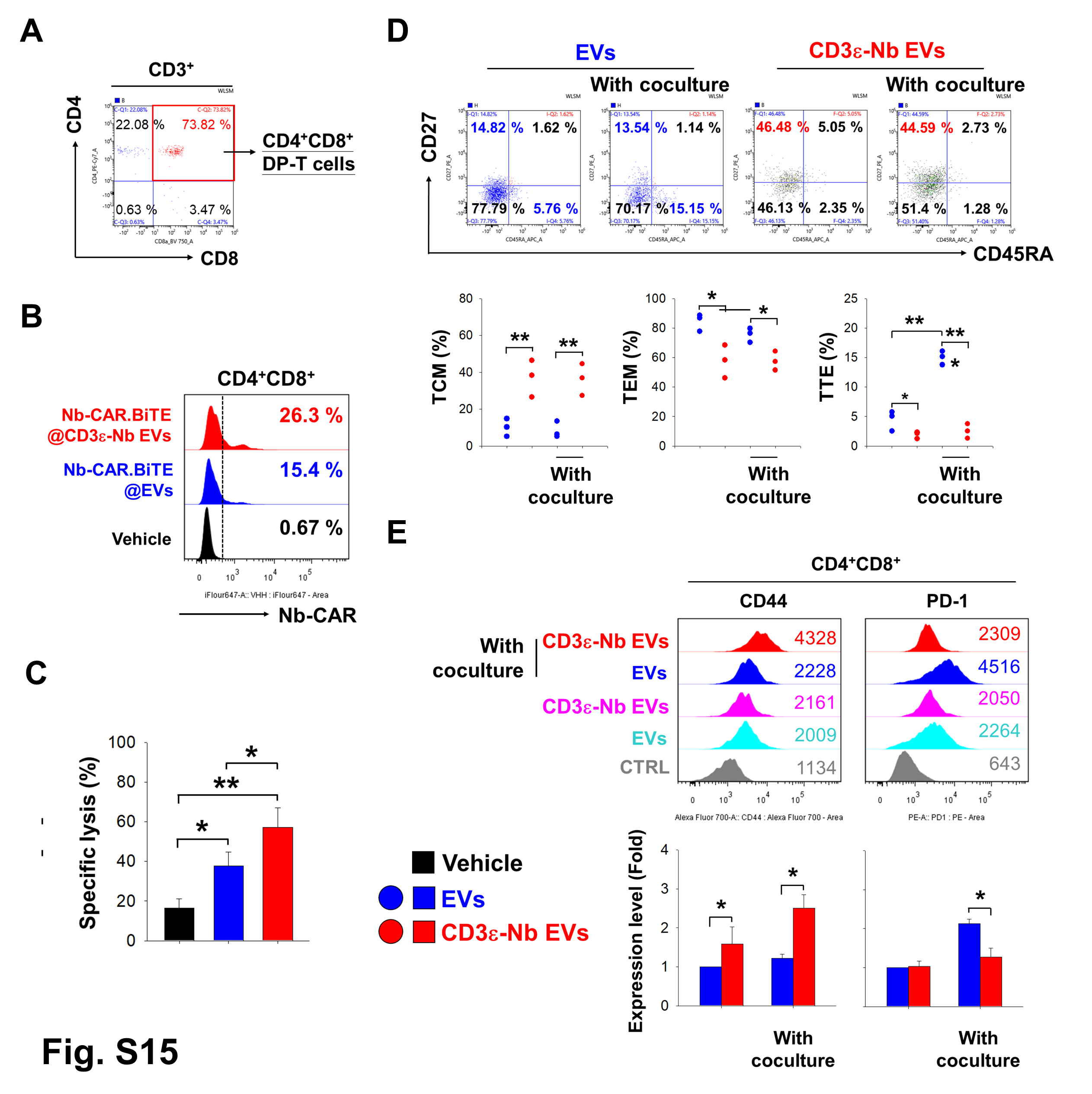


**Fig. S15** Nb-CAR.BiTE@CD3ε-Nb EVs enhanced antitumor activity of CD4^+^CD8^+^ DP cells associated with retained T-cell memory and reduced exhaustion *in vitro*. **A** The purity of PBMC-derived CD4^+^CD8^+^ DP cells was determined by flow cytometry analysis using CD4 and CD8 antibodies. After treatment with Nb-CAR.BiTE@EVs or Nb-CAR.BiTE@CD3ε-Nb EVs for 72 h, the expression levels of Nb-CAR were measured (**B**), or subsequently cocultured with COLO 205 cells at an E:T ratio of 3:1 for 72 h. The induced cytotoxicity to COLO 205 cells was determined by LIVE/DEAD Cell-Mediated Cytotoxicity Assay (**C**). The memory status(**D**) and the exhaustion markers (**E**) of CD4^+^CD8^+^ DP cells after coculturing with COLO 205 cells were determined by flow cytometry using specific antibodies against CD4, CD8, CD45RA, CD44, PD-1, CTLA-4, and TIM-3. The results of the *in vitro* experiments are representative of four independent experiments. Data are mean ± SD, **p* < 0.05; ** *p* < 0.01; *** *p* < 0.001.


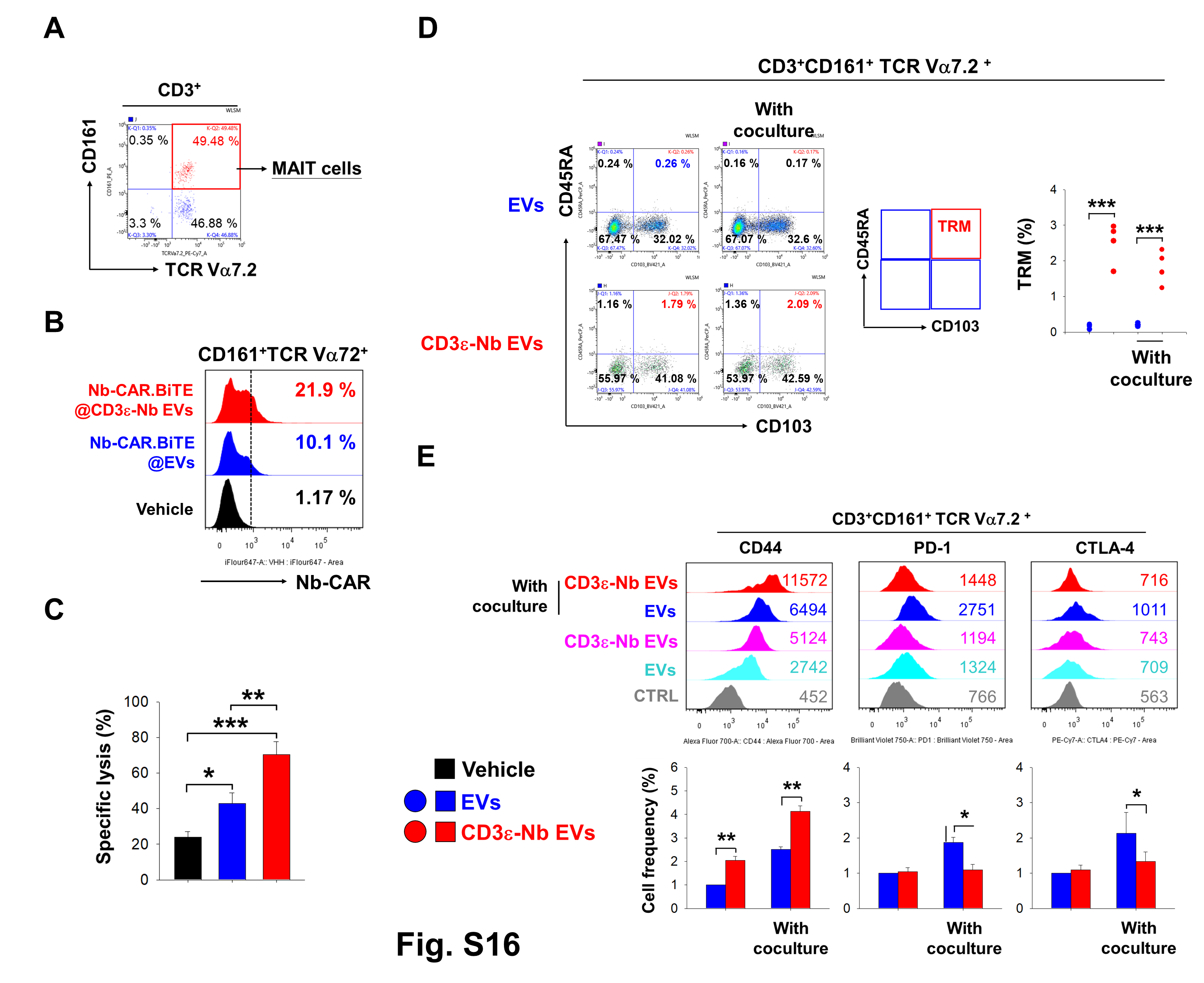


**Fig. S16** Nb-CAR.BiTE@CD3ε-Nb EVs enhanced antitumor efficiency, promoted memory, and reduced exhaustion in MAIT cells *in vitro*. **A** The purity of PBMC-derived MAIT cells was determined by flow cytometry analysis using CD3, CD161, and TCR Vα7.2 antibodies. After 72 h of treatment with Nb-CAR.BiTE@EVs and Nb-CAR.BiTE@CD3ε-Nb EVs, the Nb-CAR expression was detected (**B**). These cells were cocultured with or without COLO 205 cells at an E:T of 3:1 for 72 h. The induced cytolysis was determined by LIVE/DEAD Cell-Mediated Cytotoxicity Assay (**C**). The TRM proportions (**D**) and the exhaustion markers (**E**) on MAIT cells were determined by flow cytometry using specific antibodies against CD161, TCR Vα7.2, CD45RA, CD103, CD44, PD-1, and CTLA-4. The results of the *in vitro* experiments are representative of four independent experiments. Data are mean ± SD, **p* < 0.05; ** *p* < 0.01; *** *p* < 0.001.


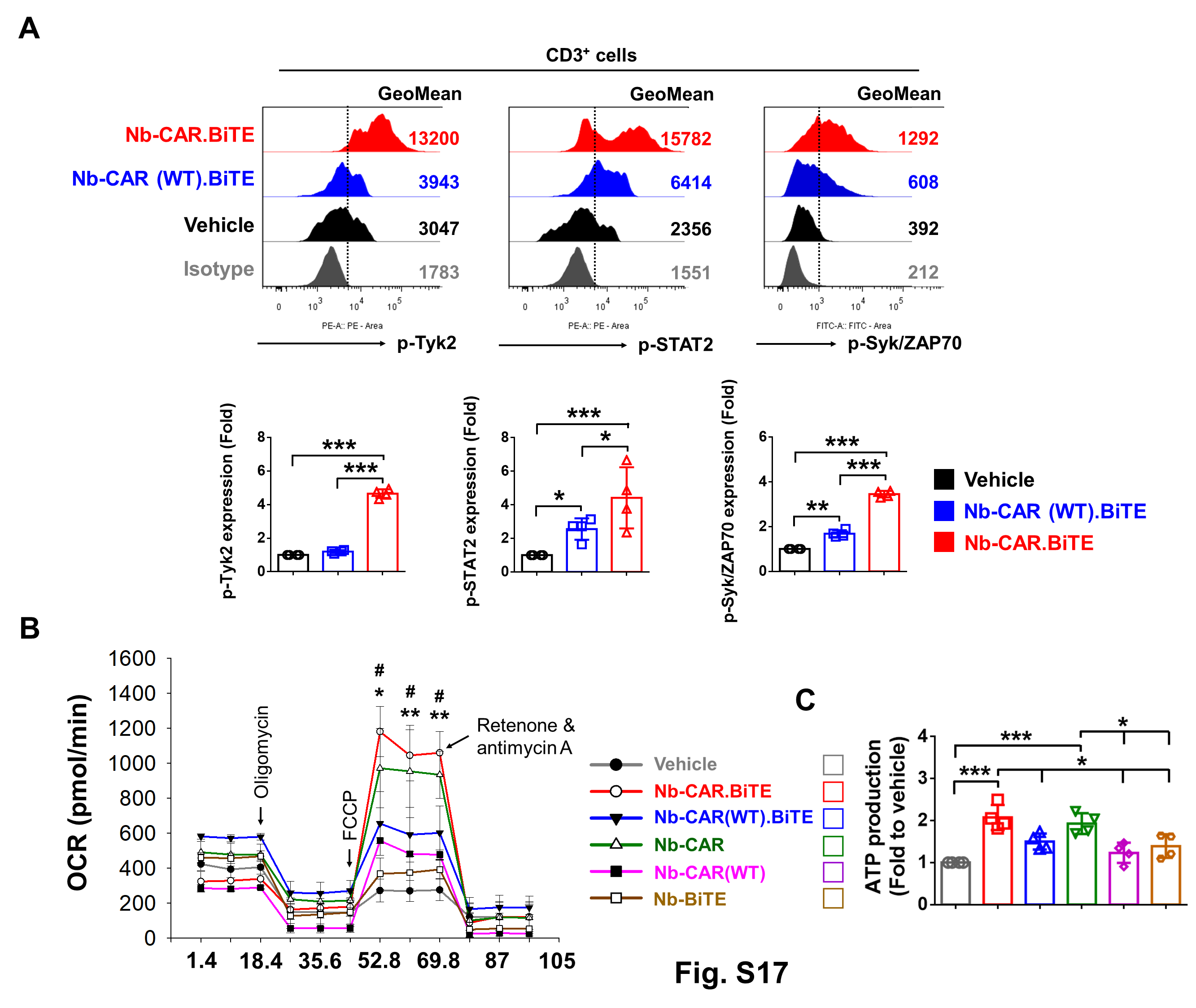


**Fig. S17** The enhanced downstream signaling and mitochondria respiration by the incorporation of modified CAR ICD in Nb-CAR.BiTE construct. After 3 days of treatment with CD3ε-Nb EVs (3 × 10^8^ particles) loading with Nb-CAR.BiTE, NCAR(WT).BiTE, Nb-CAR or Nb-CAR(WT) constructs, the PBMCs (1 × 10^8^ cells) were subjected to detect the expression levels of p-Tyk2, p-STAT2, and p-Syk/ZAP70 by flow cytometry using specific antibodies (**A**). Their oxygen consumption rate (OCR) (**B**) and ATP production capacity (**C**) were determined by Seahorse XFe24 Extracellular Flux Analyzer (Agilent Technologies). The results of the *in vitro* experiments are representative of three independent experiments. Data are presented as mean ± SD. Statistical significance was set at *p*-value < 0.05. *^,#^*p* < 0.05, *^*,##^*p* < 0.01, ****p* < 0.001; asterisk represent significant differences between Nb-CAR.BiTE and Nb-CAR(WT).BiTE; number sign represents significant differences between Nb-CAR and Nb-CAR(WT) based on paired Student’s t-tests.


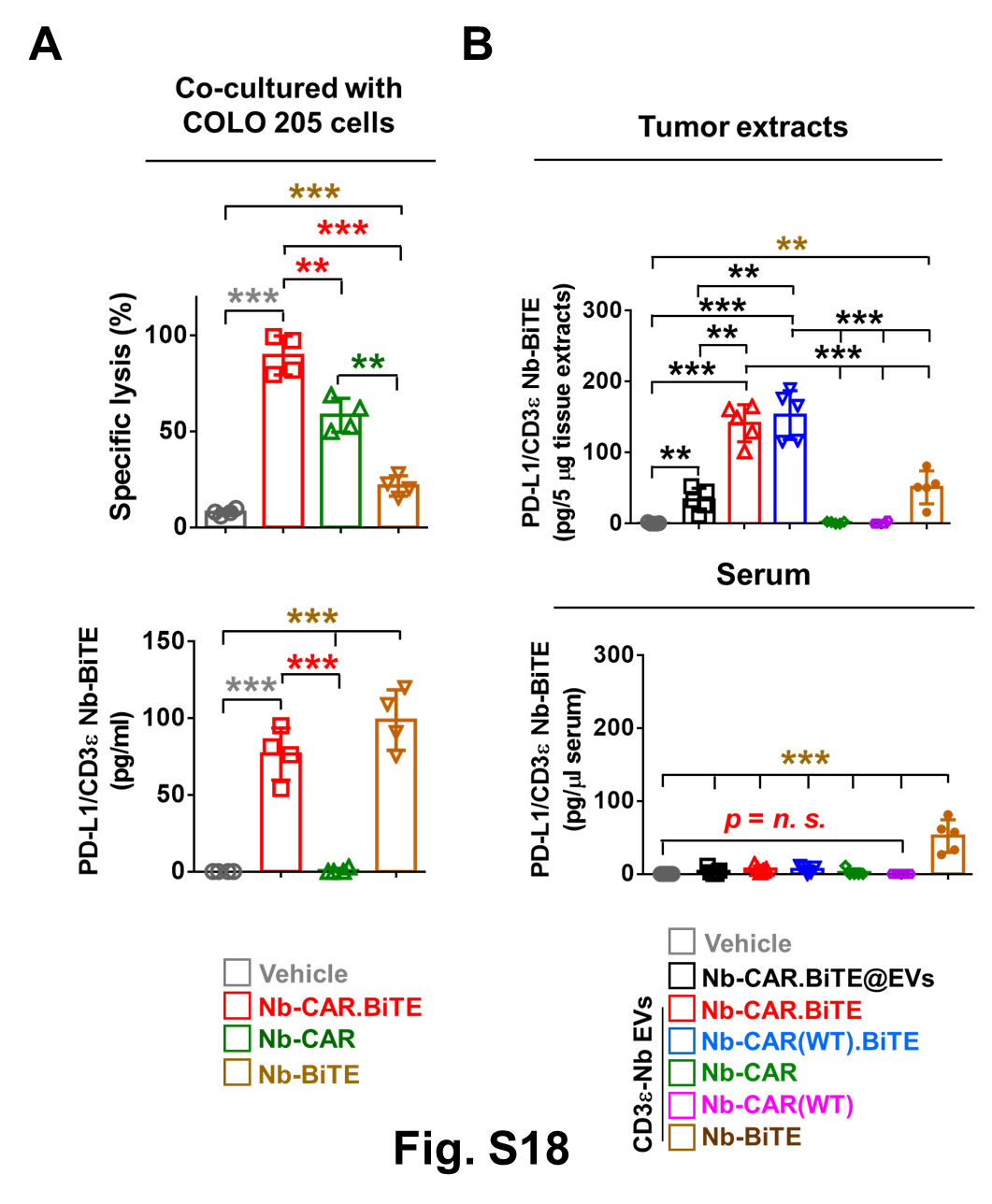


**Fig. S18** The incorporation of Nb-BiTE component contributes to the increased antitumor activity. **A** PBMCs (1 × 10^6^ cells) were treated with CD3ε-Nb EVs (3 × 10^8^ particles) loaded with Nb-CAR.BiTE, Nb-CAR, or Nb-BiTE constructs for 3 days, then cocultured with COLO 205 cells at an E:T ratio of 3:1. The induced cytotoxicity (upper panel) and secreted Nb-BiTE (bottom panel) were determined by LIVE/DEAD Cell-Mediated Cytotoxicity Assay and ELISA-based assay, respectively. Data are presented as mean ± SD, **p* < 0.05; ***p* < 0.05; ****p* < 0.05. Student’s t-test and paired Student’s t-test. **B** Schematic representation of different Nb-CAR constructs and the animal study protocol is shown in **Fig. 5A-C** in the main text. Fourteen days after the last infusion, mice were sacrificed, and the presence of Nb-BiTE in tumor extract and serum was measured by an ELISA-based assay **(B)**. The *in vivo* tumor growth data are presented as mean ± SEM and analyzed using one-way ANOVA for comparing means across multiple groups.


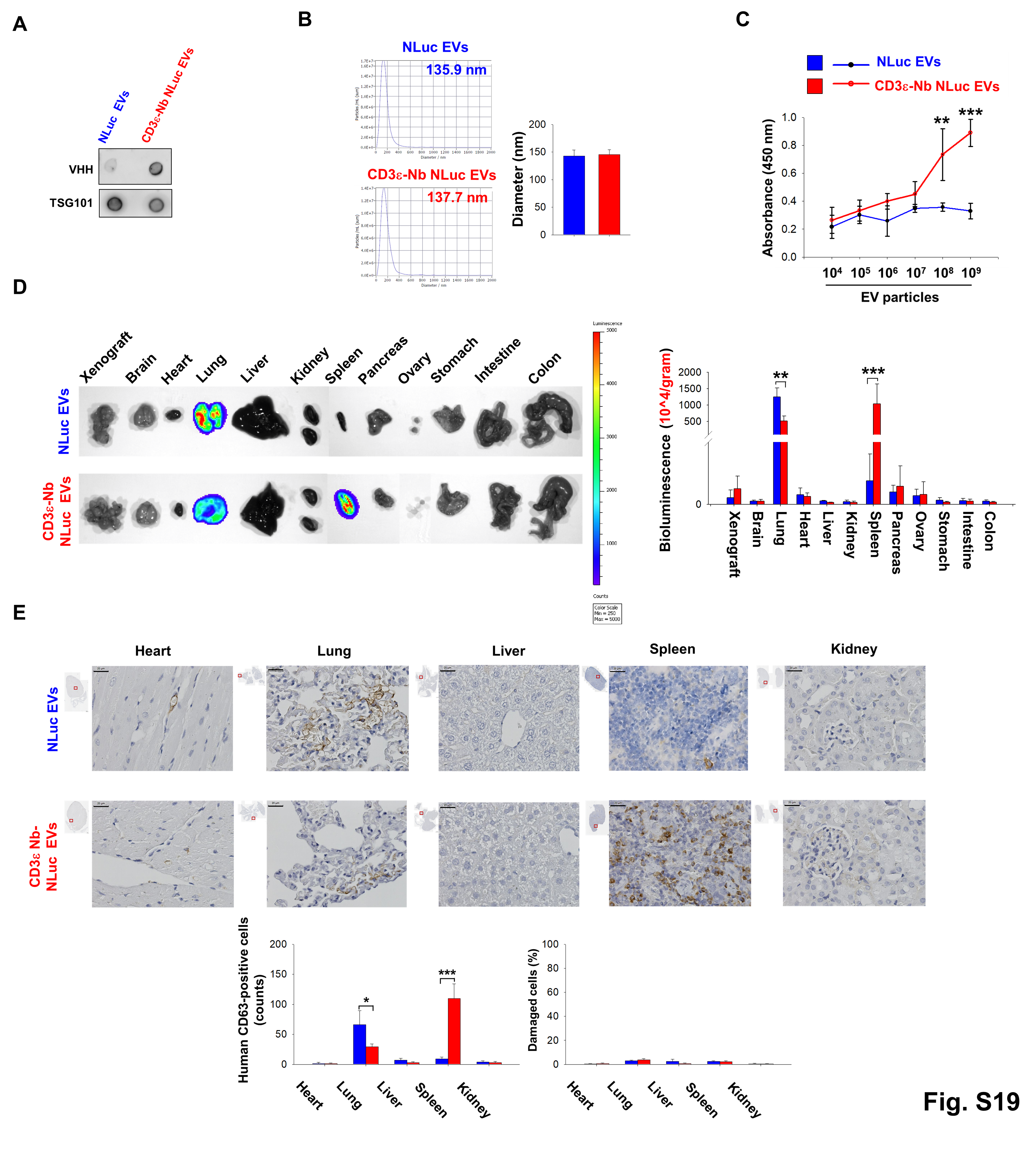


**Fig. S19** Biodistribution of CD3ε Nb- expressing EVs in COLO205 CDX-huNSG mouse model. The CD63-nanoluciferase (NLuc) and CD3ε Nb-CD63-nanoluciferase (CD3ε-Nb NLuc) chimeras were constructed withCD3ε Nb, which was connected using a GGGGS linker. This linker was inserted into the loop between the third and fourth transmembrane domains of human CD63, followed by the nanoluciferase reporter protein. **B** The VHH-expressing level of CD3ε-Nb NLuc EVs. The CD3ε-Nb NLuc EVs were harvested and isolated from the supernatants of CD3ε Nb-CD63-NLuc stably expressing HEK-293T cells using VHH-affinity resin. The presence of the CD3ε Nb components on engineered EVs was assessed by dot plot to detect the expression of CD3ε Nb moiety (VHH) and TSG101. **C** NTA analysis of HER-293T-derived NLuc EVs and CD3ε-Nb NLuc EVs. **D** Biodistribution of CD3ε Nb-expressing EVs in xenografted tumor-bearing PBMC humanized mice*.* On day 7 after intraperitoneal implantation of COLO 205-luc cells (1 × 10^6^ cells), mice (*n* = 4) were tail vein infused with huPBMCs (5 × 10^6^). Seven days later, mice were tail vein infused with NLuc EVs or CD3ε-Nb NLuc EVs (5 × 10^9^ particles). Following 5 min, these mice were injected with 100 μl of Nano-Glo®Fluorofurimazine In Vivo Substrate (Promega) through the tail vein. After 15 min of substrate infusion, the mice were sacrificed, and the presence of infused EVs in xenografted COLO 205-luc tumors, brain, lung, heart, liver, kidney, spleen, pancreas, ovary, stomach, intestine, and colon was detected by IVIS system through detecting bioluminescent signals. The bioluminescence in each organ was normalized to its weight with one gram. **E** The presence of human CD63-positive cells (reflects human CD63, which may contain NLuc constructs) in heart, lung, liver, spleen, and kidney harvested from NLuc EVs and CD3ε-Nb NLuc EV-treated COLO 205-luc tumor-bearing huNSG mice (*n* = 4) was detected by IHC staining using human CD63-specific antibody. The presence of human CD63-positive cells and the cells with damage signs (apoptosis, necrosis, fatty change, cell swelling, and surface blebbing were considered damaged cells) in each high power field (HPF) was recorded. Data are presented as mean ± SD and analyzed by Student’s t-tests and Paired t-tests. Statistical significance is set at *p-value* < 0.05. ***p* < 0.01, ****p* < 0.001.


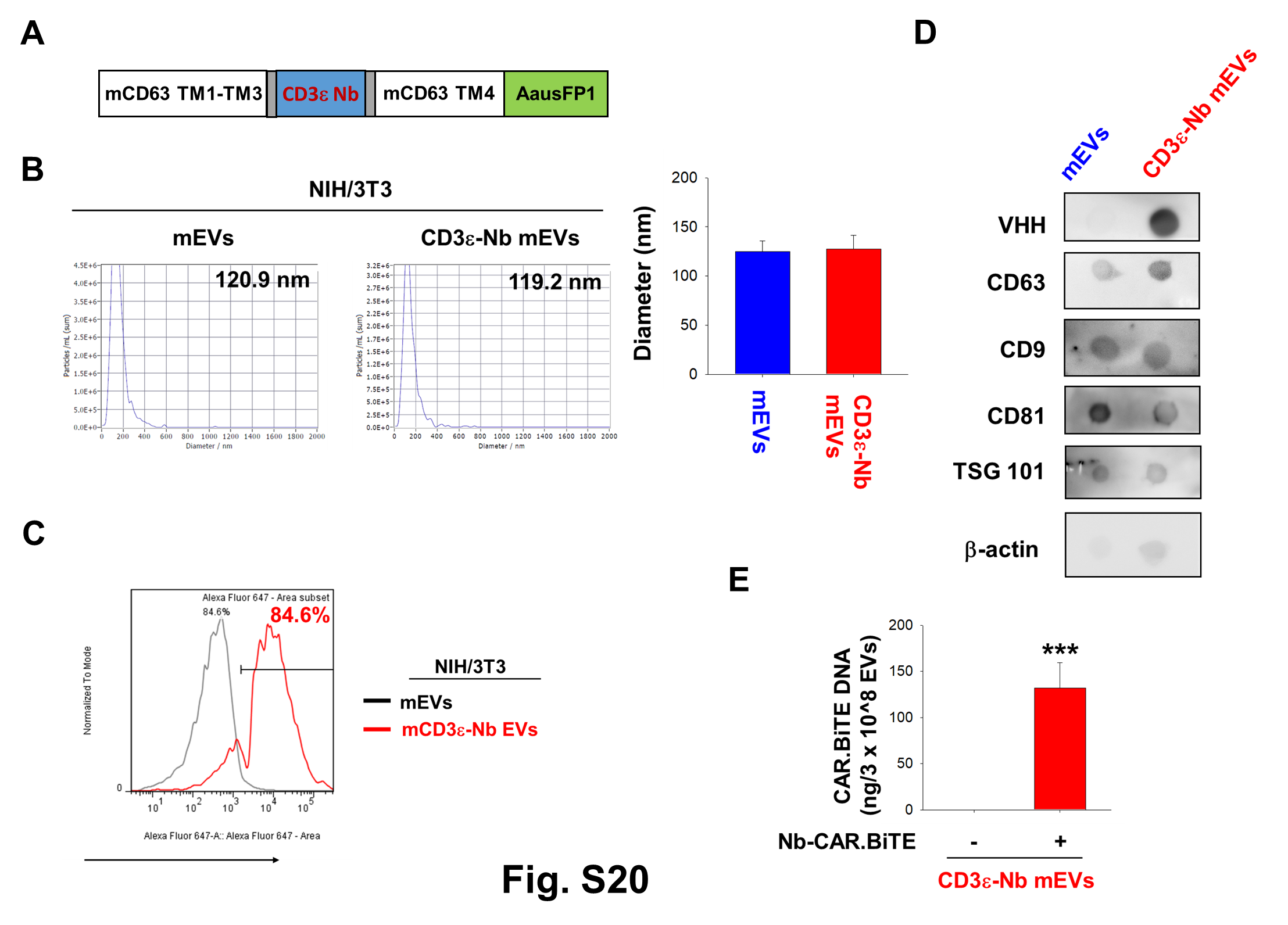


**Fig. S20** The generation of CD3ε Nb-mouse CD63-expressing EVs (CD3ε-Nb mEVs) from the mouse fibroblast cell line NIH/3T3. **A** The diagram shows the structure of the CD3ε Nb-mouse CD63 chimeric protein. Both ends of CD3ε Nb were connected with a GGGGS linker, which is inserted into the loop between the third and fourth transmembrane domains of mouse CD63, followed by the AausFP1 reporter protein. **B** NTA analysis of CD3ε-Nb mEVs and CD3ε-Nb mEVs. **C** The VHH-expressing level of CD3ε-Nb mEVs. The CD3ε-Nb mEVs were harvested from the supernatants of CD3ε Nb-mouse CD63 stably expressing NIH/3T3 cells by VHH-affinity resin. Then, the expression levels of CD3ε Nb moiety on these EVs were determined by flow cytometry using a specific antibody against VHH. **D** The presence of the CD3ε Nb components on engineered EVs was assessed by dot plot to detect the expression of CD3ε Nb moiety (VHH), along with the expressions of mouse CD63 (mCD63), mCD9, mCD81, mTSG101, and β-actin. **E** Encapsulation of Nb-CAR.BiTE transgene into NIH/3T3 cell-derived CD3ε-Nb mEVs through electroporation. CD3ε-Nb mEVs were electroporated with Nb-CAR.BiTE-linear transgene (30 μg DNA/2 × 10^9^ EVs) using LONZA 4D-Nucleofector. After incubation with DNase (1000 IU) for 15 min, the encapsulated Nb-CAR.BiTE transgene was quantified by qPCR analysis using specific primers. Results are representative of four independent experiments. Data are mean ± SD, ****p* < 0.001.


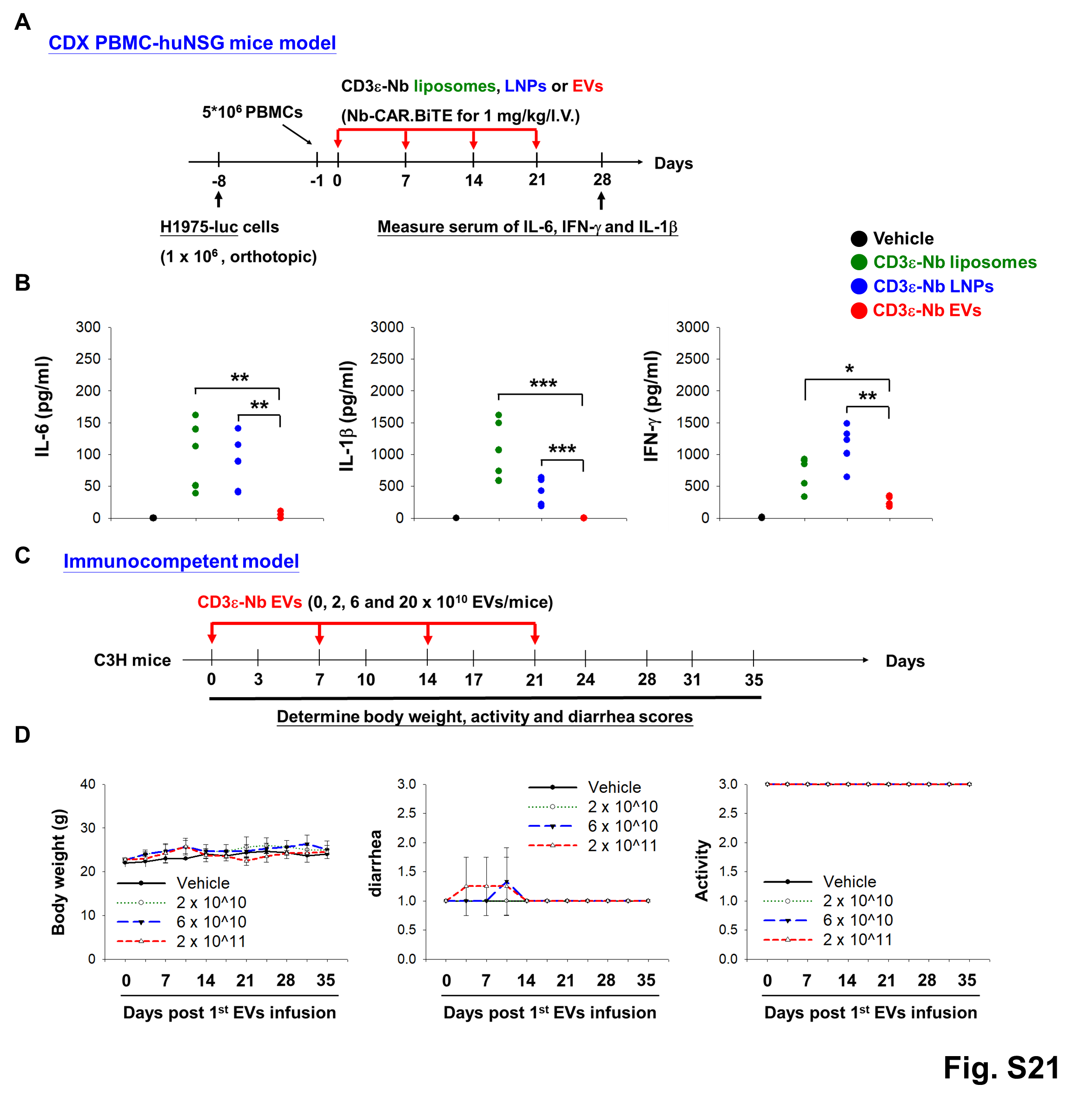


**Fig. S21** CD3ε-Nb EVs exhibited reduced immunological risks compared to lipid carrier-based approaches with minimal toxicity. CD3ε Nb-conjugated liposomes and LNPs triggered significant inflammatory cytokine responses in tumor-bearing PBMC-humanized mice compared to CD3ε Nb-expressing EVs. **A** After 7 days of orthotopic implantation with H1975-luc cells (1 × 10^6^ cells), mice (*n* = 5) were infused with human PBMCs (5 × 10^6^) via tail vein injection. The next day, mice were infused with CD3ε-Nb liposomes, CD3ε-Nb LNPs, and CD3ε-Nb EVs encapsulated with Nb-CAR.BiTE transgene (1 mg/kg) once a week for four weeks. The blood was collected on day 22 post the first EV infusion, and **B** the serum contents of IL-6, IL-1β, and IFN-γ were detected by ELISA kits according to the user instructions. **C, D** Assessment of toxicity responses induced by elevated dosages of CD3ε-Nb EVs in a mouse model. **C** C3H mice were treated with HEK-293T-derived CD3ε-Nb EVs (2 × 10^10^, 6× 10^10^, 2 × 10^11^ EVs/mouse, respectively) through tail vein injection once weekly for four weeks (*n* = 4). **D** Body weight **(left panel)**, stool morphology **(middle panel)**, and activity signs **(right panel)** were measured twice a week until 35 days post the first infusion. The results of the experiments were analyzed using one-way ANOVA to compare means across multiple groups. Statistical significance is set at *p-value* < 0.05. Data are mean ± SD, **p* < 0.05, ***p* < 0.01, ***p* < 0.001.
